# The matricellular protein Fibulin-5 regulates β-cell proliferation in an autocrine/paracrine manner

**DOI:** 10.1016/j.isci.2025.111856

**Published:** 2025-01-21

**Authors:** Tomoko Okuyama, Takahiro Tsuno, Ryota Inoue, Setsuko Fukushima, Mayu Kyohara, Anzu Matsumura, Daisuke Miyashita, Kuniyuki Nishiyama, Yusuke Takano, Yu Togashi, Makiko Meguro-Horike, Shin-ichi Horike, Tatsuya Kin, A.M. James Shapiro, Hiromi Yanagisawa, Yasuo Terauchi, Jun Shirakawa

**Affiliations:** 1 Department of Endocrinology and Metabolism, Graduate School of Medicine, Yokohama City University, Yokohama, Japan; 2 Laboratory of Diabetes and Metabolic Disorders, Institute for Molecular and Cellular Regulation (IMCR), Gunma University, Maebashi, Japan; 3 Research Center for Experimental Modeling of Human Disease, Kanazawa University, Kanazawa, Japan; 4 Clinical Islet Laboratory and Clinical Islet Transplant Program, University of Alberta, Edmonton, AB, Canada; 5 Life Science Center for Survival Dynamics, Tsukuba Advanced Research Alliance (TARA), University of Tsukuba, Tsukuba, Japan

**Keywords:** Cell biology, Functional aspects of cell biology

## Abstract

The matricellular protein Fibulin-5 (Fbln5) is a secreted protein that is essential for elastic fiber formation, and pancreatic islets are usually surrounded by the extracellular matrix (ECM), which includes elastic fibers. However, much uncertainty remains regarding the function of the ECM and its components in β-cells. Here, we describe the role of Fbln5 in β-cell replication. Fbln5 expression was increased upon glucose stimulation in β-cells of mouse and human islets. β-Cell-specific *Fbln5*-knockout (β*Fbln5*KO) mice exhibit significantly reduced β-cell proliferation *in vivo* but not *in vitro*. Secreted extracellular Fbln5 enhances β-cell replication. *Fbln5*-deficient β-cells exhibit the downregulated expression of the gene encoding Polo-like kinase 1 (PLK1), which is accompanied by ERK-mediated FoxM1 nuclear export. These data suggest that Fbln5 is secreted from β-cells in response to glucose and plays important roles in the appropriate maintenance of β-cell functions in an autocrine or paracrine manner.

## Highlights

• Fbln5 expression is upregulated by glucose in pancreatic β-cells
• β-cell-specific *Fbln5* knockout reduces β-cell proliferation *in vivo* but not *in vitro*
• Secreted Fbln5 enhances β-cell replication via an interaction with the extracellular matrix
• ERK contributes to Fbln5-mediated β-cell proliferation via FoxM1 and PLK1

## Graphical abstract

**Figure:**
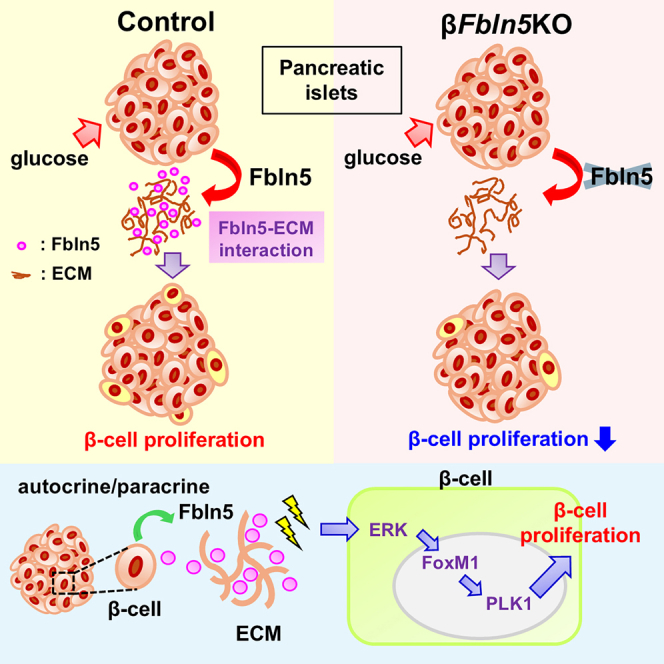

## Introduction

Various extracellular matrix (ECM) proteins are present in the basement membrane of pancreatic islets that provide structural support, promote vascularity, and facilitate cellular functions. While the vital functions of key ECM proteins such as collagen, laminin, fibronectin, and heparan sulfate proteoglycans in β-cell proliferation and survival are widely recognized, the precise interactions between the ECM and islet cells remain ambiguous.^1^^,^^2^^,^^3^^,^^4^

We previously reported that the expression of the matricellular protein Fibulin-5 (Fbln5) was significantly upregulated in islets following stimulation with glucokinase and glucose through the Ca^2+^/calcineurin/NFAT signaling pathway.^5^ Glucose metabolism mediated by glucokinase, a key component of the glycolytic pathway, has been shown to be involved in the proliferation and survival of β-cells not only in mouse islets but also in human islets.^6^^,^^7^^,^^8^^,^^9^^,^^10^ However, the detailed molecular mechanisms underlying this phenomenon remain largely unknown.^11^ Fbln5 (also known as EVEC or DANCE), a secretory glycoprotein, is essential for elastic fiber development.^12^^,^^13^ *Fbln5*-deficient mice exhibit systemic elastic fiber defects, including loose skin, a tortuous aorta, emphysematous lungs, and genital prolapse.^13^^,^^14^ Fbln5 has nonelastogenic functions, such as the regulation of protease activity, cell adhesion, and intracellular signaling via its integrin-binding RGD domain.^15^^,^^16^^,^^17^^,^^18^^,^^19^ Therefore, Fbln5 plays critical roles in the proliferation, migration, and invasion of certain tumors and smooth muscle cells.^20^^,^^21^ Under normal chow-fed conditions, systemic *Fbln5*-knockout (*Fbln5*KO) mice presented normal glucose tolerance, comparable insulin secretion, and similar rates of β-cell proliferation to their wild-type littermates.^5^ In INS-1 β-cells, *Fbln5* overexpression increased glucose-stimulated insulin secretion but potentially decreased β-cell proliferation, and the expression of the secretory protein Fbln5 in β-cells may vary with age and the cell line used.^5^ Therefore, the expression and roles of Fbln5 in the function of β-cells remain unclear.

In this study, we generated β-cell-specific *Fbln5* knockout (β*Fbln5*KO) mice to investigate the expression and functions of Fbln5 in β-cells.

## Results

### Generation of mice with a tissue-specific deletion of Fbln5 in pancreatic β-cells

**Figure 1:**
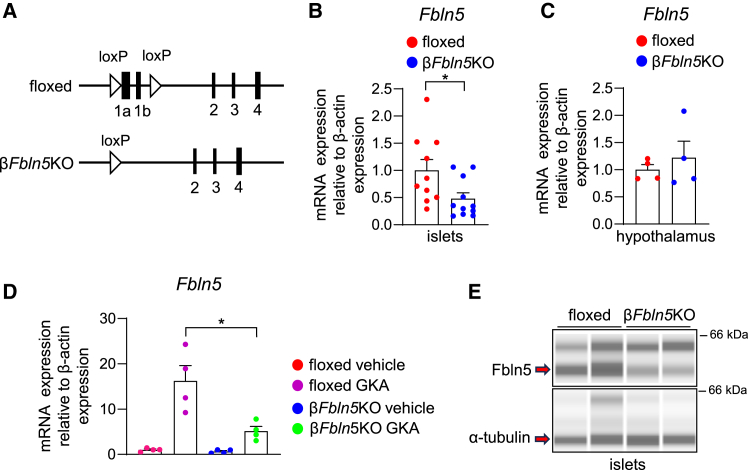
Generation of β-cell-specific *Fbln5*-knockout (β*Fbln5*KO) mice (A) Gene-targeting strategy and structure of the Fbln5-recombined alleles after Cre-dependent recombination. (B) *Fbln5* mRNA expression levels in islets isolated from 11-week-old β*Fbln5*KO and control mice (*n* = 11 mice in β*Fbln5*KO group, *n* = 10 mice in the control group). The data are presented as the means ± SEMs. ∗*p* < 0.05, Student’s t test. (C) *Fbln5* mRNA expression levels in the hypothalamus of β*Fbln5*KO and control mice (*n* = 4 mice per group). The data are presented as the means ± SEMs. (D) *Fbln5* mRNA expression levels in islets from 11-week-old β*Fbln5*KO mice and their control littermates after an incubation with 30 μM GKA CpdA for 24 h (*n* = 4 samples per group). The data are presented as the means ± SEMs. ∗*p* < 0.05, one-way ANOVA followed by the Tukey HSD post-hoc test. (E) Immunoblotting showing Fbln5 in islets isolated from 11-week-old β*Fbln5*KO mice and their control littermates.

Using *Ins1*-Cre mice crossed with *Fbln5*-floxed mice, we generated β-cell-specific *Fbln5*-conditional knockout mice (Figure 1A). Compared with control (Fbln5-floxed) islets, islets from β*Fbln5*KO mice presented an approximately 50% reduction in *Fbln5* mRNA expression (Figure 1B), indicating that *Fbln5* is expressed in the β-cells of islets. Compared with that in control mice, *Fbln5* expression in the hypothalamus was not affected in β*Fbln5*KO mice (Figure 1C). Compared with islets isolated from global *Fbln5*KO mice, β*Fbln5*KO islets presented higher expression of *Fbln5*, suggesting that Fbln5 is expressed not only in β-cells but also in nonβ-cells in islets (Figure S1A). The extracellular tissue attached to the islets might account for higher *Fbln5* mRNA expression in β*Fbln5*KO islets than in systemic *Fbln5*KO islets, as we previously reported Fbln5 expression in interstitial endothelial cells or small vessels in the islets.^5^ The induction of *Fbln5* expression by GKA stimulation was also significantly attenuated in β*Fbln5*KO islets (Figure 1D). The expression of the Fbln5 protein was reduced in the islets from β*Fbln5*KO mice compared with those from control mice (Figures 1E and S1B).

### Localization of Fbln5 in the endocrine pancreas

**Figure 2:**
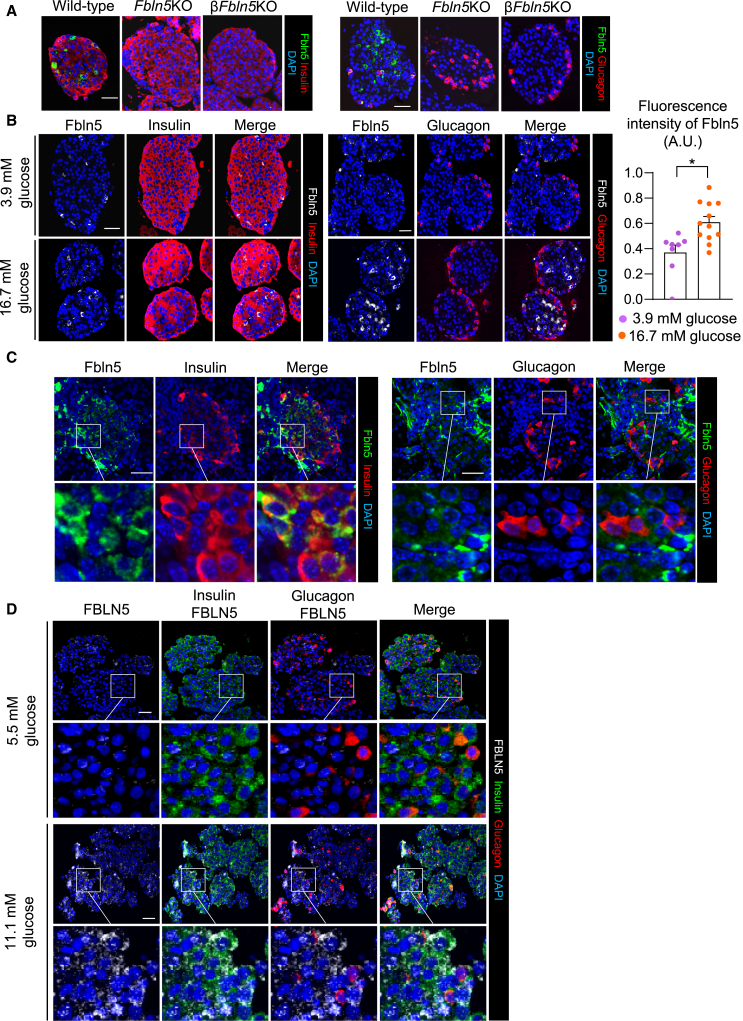
Fbln5 expression in mouse and human islets under low-glucose or high-glucose conditions (A) Representative pancreatic islet sections from 11- to 12-week-old wild-type, *Fbln5*KO, and β*Fbln5*KO mice stained with antibodies against Fbln5 (green), insulin (red in the left panels) and glucagon (red in the right panels) are shown. Nuclei are stained blue with 4′, 6-diamidino-2-phenylindole [DAPI]. The scale bar represents 30 μm. Islets were incubated for 24 h with 4.4 mM glucose before being embedded in agarose. (B) Isolated mouse islets were treated with 3.9 mM glucose or 16.7 mM glucose for 24 h. Representative islet sections from 14- to 16-week-old wild-type mice stained with antibodies against Fbln5 (gray), insulin (red in the left panels) and glucagon (red in the right panels) are shown. Nuclei are stained blue with DAPI. The scale bar represents 30 μm. The fluorescence intensity of Fbln5 normalized to that of insulin was calculated at 5 randomly selected areas per islet using densitometry (4–6 islets per mouse, 4 mice per group). The data are presented as the means ± SEMs. ∗*p* < 0.05, Student’s t test. (C) Representative pancreatic sections from postnatal Day 6 of wild-type mice stained with antibodies against Fbln5 (green) and insulin (red in the left panels) or glucagon (red in the right panels) are shown. Nuclei are stained blue with DAPI. The scale bar represents 20 μm. (D) Representative human islet sections stained with antibodies against FBLN5 (gray), insulin (green) and glucagon (red) are shown. Nuclei are stained blue with DAPI. The scale bar represents 50 μm. Islets were treated for 24 h with 5.5 mM glucose or 11.1 mM glucose.

We detected the localization of Fbln5 predominantly in insulin-positive β-cells but not in glucagon-positive α-cells in the islets of control mice (Figures 2A and 2B). Fbln5 fluorescence signals were not evident in the islets of systemic *Fbln5*-deficient mice (*Fbln5*KO) or β*Fbln5*KO mice (Figure 2A). In addition, Fbln5 expression was increased by treatment with a high level of glucose compared with treatment with a low level of glucose, in mouse islets (Figure 2B). As we previously revealed that Fbln5 expression decreases with age in mouse islets,^5^ immunofluorescence staining for Fbln5 seemed to be higher in postnatal Day 6 mouse β-cells (Figure 2C) than in adult mouse β-cells, as shown in Figure 2A. We further elucidated the localization of FBLN5 in human islets from a nondiabetic donor. FBLN5 expression was also more dominant in β-cells than in α-cells in human islets, and the signal was enhanced by a high level of glucose (Figures 2D and S2). Although Fbln5 is a secreted extracellular matrix protein and we previously reported its localization around interstitial tissue,^5^ we did not observe Fbln5 expression in the interstitium or extracellular matrix of isolated islets, possibly because extracellular Fbln5 was washed out by the process of culture and deparaffinization. We examined the costaining of Fbln5 with Lamin β1, integrin β1, and CD31 in mouse pancreas sections and revealed that Fbln5 did not localize with those ECM proteins in islets (Figure S3). We also confirmed that the expression of those ECM proteins was not inhibited in β*Fbln5*KO islets (Figure S3). On the other hand, in the analysis of single-cell RNA sequencing datasets for pancreatic islets from normal chow-fed and high-fat diet-fed mice,^22^ we detected the coexpression of Fbln5 in CD31 (Pecam1)- or CD34-positive cells (Figure S4). These results indicate that Fbln5 is expressed in endothelial cells, although it is not dominantly expressed in islets.

### Detection of Fbln5 expression in islet β and nonβ cells by single-cell RNA sequencing

**Figure 3:**
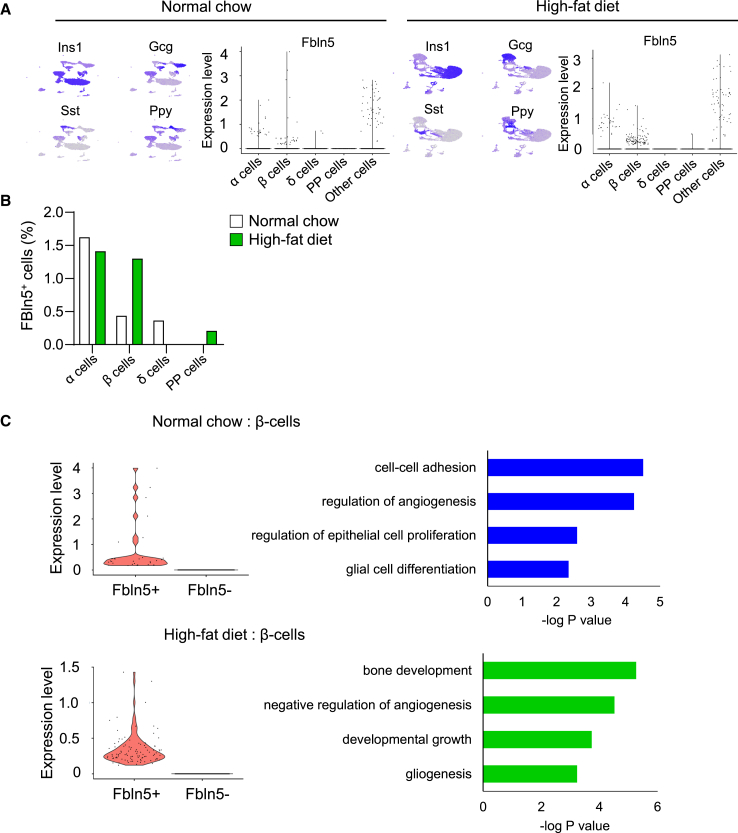
Single-cell RNA sequencing analysis of islets from normal chow-fed mice and high-fat diet-fed mice (A) Feature plot of endocrine hormones and violin plot of Fbln5 expression in each cell type in islets from normal chow-fed mice (left panel) and high-fat diet-fed mice (right panel). (B) The ratio of Fbln5-positive β-cells in normal chow-fed and high-fat diet-fed mice. (C) Enriched pathways of genes whose expression levels exhibited a fold change >2.5 and adjusted *p* value >0.05 between Fbln5-positive and Fbln5-negative β-cells from normal chow-fed (upper panel) and high-fat diet-fed (lower panel) mice.

We reanalyzed existing islet single-cell RNA sequencing datasets from normal chow-fed mice and 8-week of high-fat diet-fed mice, as well as from wild-type mice and db/db mice, to explore Fbln5 expression in pancreatic islet β-cells and nonβ cells.^22^^,^^23^ Fbln5-positive cells were detected not only in β-cells but also in α-cells and nonβ islet endocrine cells (Figures 3A and 3B). Importantly, high-fat diet feeding increased the expression of Fbln5 in β-cells but not in α-, δ-, or PP-cells, and Fbln5 expression in β-cells was decreased in db/db mice^23^ (Figures S5A‒S5C). These results support the expression of Fbln5 in β-cells and its β-cell-specific induction by high-fat diet feeding, in which β-cell proliferation is reportedly promoted via glucose signaling. In addition, a pathway analysis of differentially expressed genes in Fbln5-positive β-cells from both normal chow-fed and high-fat diet-fed mice revealed pathways related to cell proliferation, such as cell-cell adhesion, epithelial cell proliferation, and developmental growth (Figure 3C). Next, we evaluated the expression of proliferative markers and integrin-related molecules in Fbln5-positive and Fbln5-negative β-cells in the islets of high-fat diet-fed mice or db/db mice (Figures S6A and S6B). The expression of proliferative markers, such as Ki67 and cyclin D1, and integrin-related molecules, such as integrin β1, ILK, and FAK, was not specifically altered in Fbln5-positive β-cells.

### Impact of the β-cell-specific deficiency of Fbln5 on glucose intolerance

**Figure 4:**
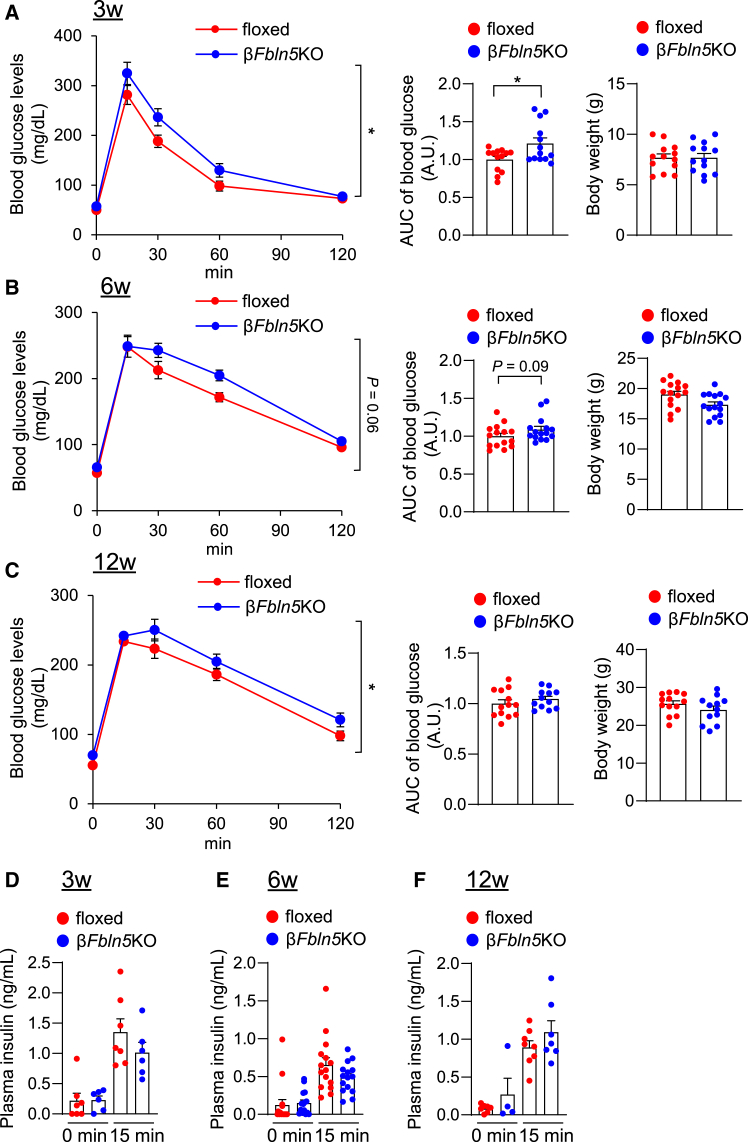
The loss of Fbln5 in pancreatic β-cells was associated with slight glucose intolerance (A–C) Blood glucose levels during an oral glucose tolerance test (OGTT) and body weight gains in 3-, 6-, and 12-week-old β*Fbln5*KO and control mice (*n* = 13 mice per group in Figure 4A; *n* = 15 mice per group in Figure 4B; *n* = 12–13 mice per group in Figure 4C). The AUC of blood glucose is shown in the middle graph, and the plasma insulin levels are shown in the right graph. The data are presented as the means ± SEMs. ∗*p* < 0.05, two-way ANOVA in the left graph and Student’s t test in the middle graph. (D–F) Plasma insulin levels in the OGTT at 0 and15 min. The data are presented as the means ± SEMs.

We performed an OGTT in 3-, 6-, and 12-week-old β*Fbln5*KO mice. *Fbln5* deletion in β-cells caused slight glucose intolerance in β*Fbln5*KO mice at 3 and 12 weeks of age (Figures 4A–4C) without impaired insulin secretion (Figures 4D–4F). No apparent difference in plasma insulin levels was observed between the 8- to 12-week-old β*Fbln5*KO mice and the control mice during the first and second phases of insulin secretion in response to glucose stimulation (Figures S7A–S7C). The degree of body weight gain did not differ significantly between the β*Fbln5*KO and control mice at 3 and 12 weeks of age (Figures 4A–4C). No changes were observed in glucose tolerance or insulin secretion during the glucose tolerance test in β*Fbln5*KO mice after 12 weeks of high-fat diet feeding (Figures S7D and S7E). Since some β-cell-specific Cre mice have altered glucose tolerance caused by the *hGH* gene,^24^ we evaluated the impact of *Ins1*-Cre on glycemic control in this study. However, no significant difference in glucose tolerance was observed between *Ins1*-Cre mice and control mice at 6–7 weeks of age (Figures S8A and S8B).

### Fbln5 deletion in β-cells allowed comparable insulin secretion in isolated islets

**Figure 5:**
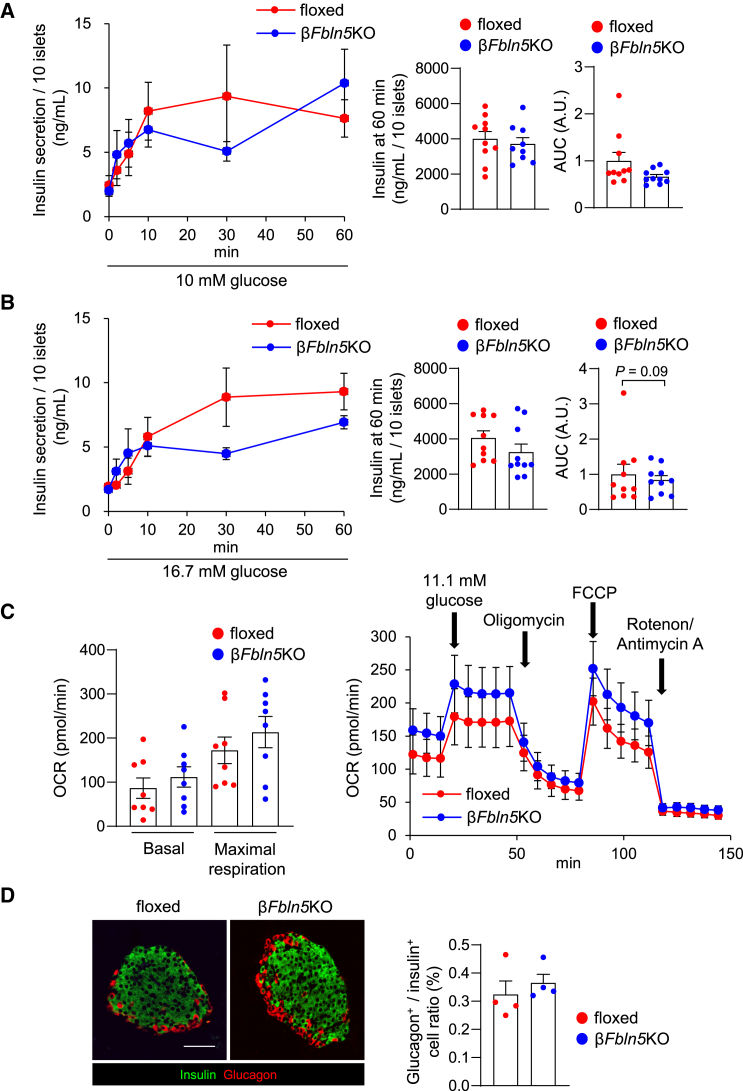
Time- and dose-dependent glucose-stimulated insulin secretion in isolated islets was not significantly attenuated in the islets from β*Fbln5*KO mice (A) Left panel: Glucose-stimulated insulin secretion (GSIS) in 10 isolated islets from 12- to 13-week-old β*Fbln5*KO mice and control mice (*n* = 10 per group). Insulin levels in the medium were measured at 0, 2, 5, 10, 30, and 60 min after 10 mM glucose stimulation. Middle panel: Insulin content after an incubation with 10 mM glucose for 1 h in 10 isolated islets from 12- to 13-week-old β*Fbln5*KO mice and control mice (*n* = 10 per group). Right panel: The AUC of the left graph is shown. The data are presented as the means ± SEMs. (B) Left panel: Glucose-stimulated insulin secretion (GSIS) in 10 isolated islets from 12- to 13-week-old β*Fbln5*KO mice and control mice (*n* = 10 per group). Insulin levels in the medium were measured at 0, 2, 5, 10, 30, and 60 min after stimulation with 16.7 mM glucose. Middle: Insulin content after an incubation with 16.7 mM glucose for 1 h in 10 isolated islets from 11-week-old β*Fbln5*KO mice and control mice (*n* = 10 per group). Right panel: The AUC of the left graph is shown. The data are presented as the means ± SEMs. (C) Islet respirometry. Twenty islets isolated from 11-week-old β*Fbln5*KO mice and control mice were seeded onto an XF96 spheroid plate, and maximal respiration (followed by 11.1 mM glucose, 4 μM oligomycin and 1 μM FCCP) was measured (*n* = 8 per group). The data are presented as the means ± SEMs. (D) Left panel: Immunohistochemical staining for insulin and glucagon. Paraffin-embedded pancreas sections from 11-week-old mice were stained with antibodies against insulin (green) and glucagon (red). The scale bar represents 50 μm. Right panel: The ratio of α-to β-cells in islets from 11-week-old control mice and β*Fbln5*KO mice (*n* = 4 per group). The data are presented as the means ± SEMs.

Next, we examined glucose-stimulated insulin secretion in islets isolated from β*Fbln5*KO and control mice after treatment with 10 mM and 16.7 mM glucose, sequentially (Figures 5A and 5B). Compared with control islets, islets isolated from β*Fbln5*KO mice presented no significant changes in insulin secretion or the insulin content (Figures 5A and 5B). GLP-1 receptor signaling and downstream cyclic AMP production play important roles in glucose-dependent insulin secretion.^25^^,^^26^ Since *Fbln5* expression in islets is induced by glucose stimulation, we next investigated the interaction between Fbln5 and GLP-1 signaling using liraglutide, a GLP-1 receptor agonist, and forskolin, an adenylate cyclase activator. No significant difference in the insulin secretory response to liraglutide stimulation was observed between the islets isolated from the β*Fbln5*KO mice and those isolated from the control mice (Figure S9A). The insulin content tended to be reduced in the islets isolated from the β*Fbln5*KO mice (Figure S9B). In addition, *Fbln5* mRNA expression in islets was significantly induced by treatment with forskolin (Figure S10A). Furthermore, the induction of *Fbln5* expression in islets by forskolin was reversed by treatment with a PKA inhibitor or an Epac2 inhibitor (Figure S10B), indicating that cyclic AMP possibly increases Fbln5 expression levels in islets.

No significant difference in the maximal respiration measured through respirometry using an extracellular flux analyzer was observed between islets from β*Fbln5*KO mice and those from control mice (Figure 5C). In addition, the mRNA expression levels of genes related to mitochondrial function, such as *Drp1*, *Tfam*, and *Opa1*, were not altered by the deletion of *Fbln5* in β-cells (Figure S11). These results suggest that normal mitochondrial functions are maintained in β*Fbln5*KO islets. No significant differences in the islet morphology, distribution, or in the proportion of α- or β-cells in the islets were observed between the control and β*Fbln5*KO mice (Figure 5D).

### Potential signaling pathways involved in the function of Fbln5 in β-cells

**Figure 6:**
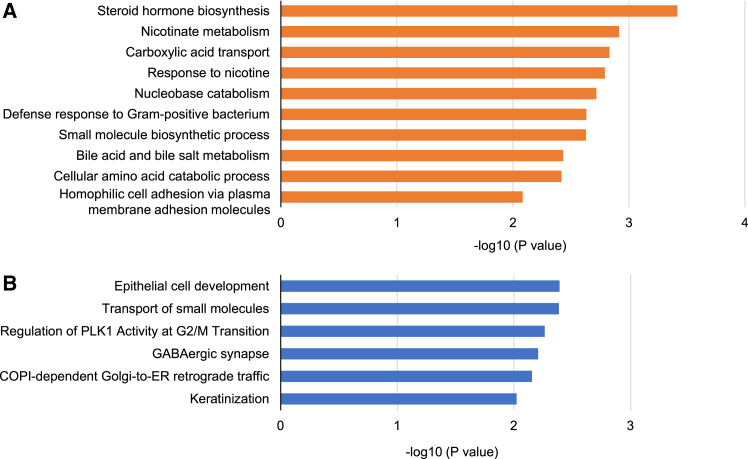
Gene Ontology (GO) analysis of islets isolated from β*Fbln5*KO and control mice (A and B) Microarray-based Gene Ontology analysis. A summary of the biological process classification of significantly upregulated (A) and downregulated (B) genes in β*Fbln5*KO islets compared with control islets is shown (*n* = 4 per group). Analyses were performed on 312 and 171 genes whose expression was significantly (*p* < 0.05) upregulated or downregulated, respectively. Bars represent -log_10_ (*p* value).

We analyzed a gene expression microarray of isolated islets and compared the results between β*Fbln5*KO and control mice to identify the molecules involved in the Fbln5-mediated regulation of β-cell functions. The results revealed the significant upregulation of 312 genes and significant downregulation of 171 genes in β*Fbln5*KO islets (*p* < 0.05). The 20 genes whose expression was most upregulated or downregulated in the β*Fbln5*KO islets are shown in Tables 1 and 2. Notably, the expression of solute carrier family transporters was altered by the deletion of *Fbln5* in β-cells. In addition, 6 genes encoding olfactory receptors and netrin 5 were among the 20 genes with the most upregulated expression, whereas the genes encoding *Sema3d*, *Gpr33*, and adenylate cyclase 10 were among the 20 genes with the most downregulated expression in β*Fbln5*KO islets. Gene Ontology (GO) analysis revealed that several metabolic pathways, including steroid metabolism via cytochrome P450, nicotinate metabolism, and carboxylic acid transport, were related to the genes whose expression was upregulated in β*Fbln5*KO islets (Figure 6A). GO analysis revealed biological functions such as epithelial cell development, the transport of small molecules, and the regulation of Polo-like kinase 1 (PLK1) activity during the G2/M transition among the activities of the downregulated genes (Figure 6B).

**Table 1:** Top 20 upregulated genes in β*Fbln5*KO islets revealed by the microarray analysis

FC	Gene Symbol	Gene Name
58.55	Slc38a1	Solute carrier family 38, member 1
17.66	Esp36	exocrine gland secreted peptide 36
15.38	Sft2d1	SFT2 domain containing 1
15.22	Olfr390	olfactory receptor 390
15.06	Jakmip2	janus kinase and microtubule interacting protein 2
14.75	Olfr630	olfactory receptor 630
14.28	Ccdc141	coiled-coil domain containing 141
13.75	Olfr938	olfactory receptor 938
11.19	Olfr491	olfactory receptor 491
9.66	Slco1b2	solute carrier organic anion transporter family, member 1b2
9.66	Cyp4a12a	cytochrome P450, family 4, subfamily a, polypeptide 12a
9.27	Tnfrsf22	tumor necrosis factor receptor superfamily, member 22
8.19	Olfr707	olfactory receptor 707
7.67	Ugt1a10	UDP glycosyltransferase 1 family, polypeptide A10
7.49	Olfr1240	olfactory receptor 1240
7.29	Ntn5	netrin 5
6.65	Cxcl9	chemokine (C-X-C motif) ligand 9
6.53	Chrnb4	cholinergic receptor, nicotinic, beta polypeptide 4
6.44	Uroc1	urocanase domain containing 1
6.26	Vmn1r223	vomeronasal 1 receptor 223

The top 20 upregulated genes according to the gene expression microarray analysis of islets isolated from β*Fbln5*KO and control mice are shown. FC, fold change.

**Table 2:** Top 20 downregulated genes in β*Fbln5*KO islets revealed by the microarray analysis

FC	Gene Symbol	Gene Name
19.04	Sema3d	sema domain, immunoglobulin domain (Ig), short basic domain, secreted, (semaphorin) 3D
16.87	Krt13	keratin 13
11.90	Gpr33	G protein-coupled receptor 33
5.22	Slc5a2	solute carrier family 5 (sodium/glucose cotransporter), member 2
4.75	Rbbp8nl	RBBP8 N-terminal like
4.67	Wdsub1	WD repeat, SAM and U-box domain containing 1
4.32	Slc23a2	solute carrier family 23 (nucleobase transporters), member 2
4.21	Slc43a1	solute carrier family 43, member 1
4.10	Onecut1	one cut domain, family member 1
3.79	Pgbd1	piggyBac transposable element derived 1
3.74	Adgb	androglobin
3.61	Zcwpw1	zinc finger, CW type with PWWP domain 1
3.48	Olfr1294	olfactory receptor 1294
3.46	Adamts10	A disintegrin and metalloproteinase with thrombospondin motifs 10
3.38	Zfp12	zinc finger protein 12
3.30	Ppp1r12b	protein phosphatase 1, regulatory (inhibitor) subunit 12B
3.08	Adcy10	adenylate cyclase 10
3.06	Naip6	NLR family, apoptosis inhibitory protein 6
2.98	Krt2	keratin 2
2.93	Nup98	nucleoporin 98

The top 20 downregulated genes from the gene expression microarray analysis of islets isolated from β*Fbln5*KO and control mice are shown. FC, fold change.

### Fbln5 deletion in β-cells attenuated β-cell proliferation

**Figure 7:**
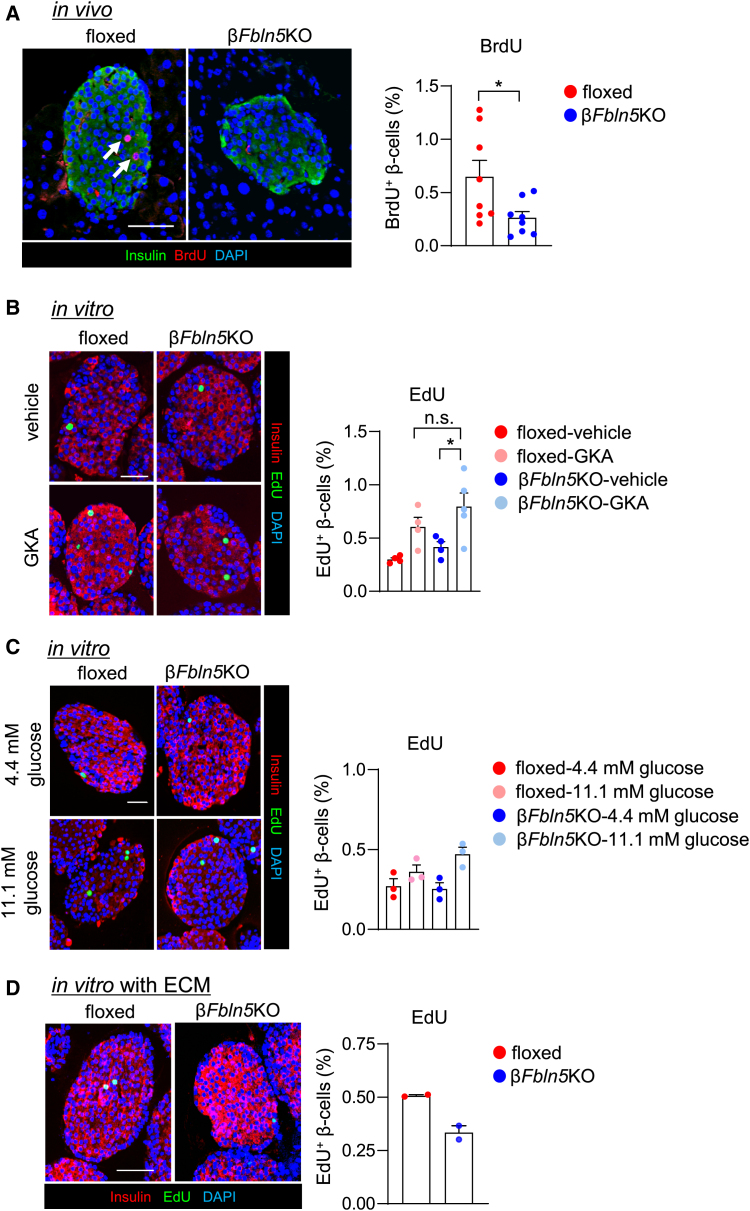
Fbln5 deletion in β-cells reduced β-cell proliferation (A) BrdU incorporation in pancreas sections from 11- to 12-week-old β*Fbln5*KO mice and control mice. BrdU was administered 6 h before the animals were sacrificed. Representative images of pancreatic islets (left panel) and the percentage of BrdU-positive β-cells among the insulin-positive β-cells (right panel) are shown (*n* = 8 per group). Insulin is stained green, the cell nuclei are stained blue with DAPI, and the BrdU-positive nuclei are stained red. The white arrows indicate BrdU-positive β-cells. The scale bar represents 50 μm. The data are presented as the means ± SEMs. ∗*p* < 0.05, Student’s t test. (B and C) EdU incorporation in islets isolated from 11- to 12-week-old β*Fbln5*KO mice and control mice. Representative images of pancreatic islets after 24 to 48 h of incubation with EdU (left panel) and the percentage of EdU-positive β-cells among the insulin-positive β-cells (right panel) are shown (*n* = 4 in the floxed-vehicle group, floxed-GKA group, and β*Fbln5*KO-vehicle group in B; *n* = 5 in the β*Fbln5*KO-GKA group in B; *n* = 3 per group in C). Insulin is stained red, the cell nuclei are stained blue with DAPI, and the EdU-positive nuclei are stained green. The scale bar represents 30 μm. The data are presented as the means ± SEMs. ∗*p* < 0.05, one-way ANOVA followed by the Tukey HSD post hoc test. (D) Islets isolated from β*Fbln5*KO and control mice were incubated on Matrigel with EdU for 24 h in the presence of 4.5 mM glucose. The percentages of EdU-positive β-cells among the insulin-positive β-cells (right panel) are shown (*n* = 2 per group). The data are presented as the means ± SEMs.

Because Fbln5 was predicted to affect the PLK1-mediated cell replication pathway in the above analysis, we evaluated the impact of β-cell-specific *Fbln5* deletion on β-cell proliferation. The proportion of BrdU-positive proliferative β-cells was markedly reduced in β*Fbln5*KO mice *in vivo* (Figure 7A). The reduced cell proliferation in β*Fbln5*KO mice was also supported by the decreased proportion of Ki67-positive β-cells or pHH3-positive β-cells (Figures S12A and S12B). In high-fat diet-fed mice, the increase in β-cell proliferation was more significantly attenuated by the deletion of *Fbln5* in β-cells (Figure S12C), although the β-cell mass was comparable between β*Fbln5*KO mice and control mice under both normal chow-fed (data not shown) and high-fat diet-fed conditions (Figure S12D). On the other hand, the incorporation of EdU into β-cells of cultured islets *in vitro* was comparable between cells originating from control and β*Fbln5*KO mice under both basal conditions and after GKA or high-glucose stimulation (Figures 7B and 7C). When the islets were incubated with Matrigel to mimic *in vivo* conditions with a surrounding extracellular matrix, the incorporation of EdU into β-cells was decreased by the deletion of *Fbln5* in β-cells, suggesting the importance of the extracellular matrix in Fbln5-mediated β-cell proliferation (Figure 7D). Thus, the effect of β-cell-derived Fbln5 on β-cell proliferation might depend on its circumstances with or without an extracellular matrix.

### Pathway and mechanism of Fbln5-mediated β-cell proliferation

**Figure 8:**
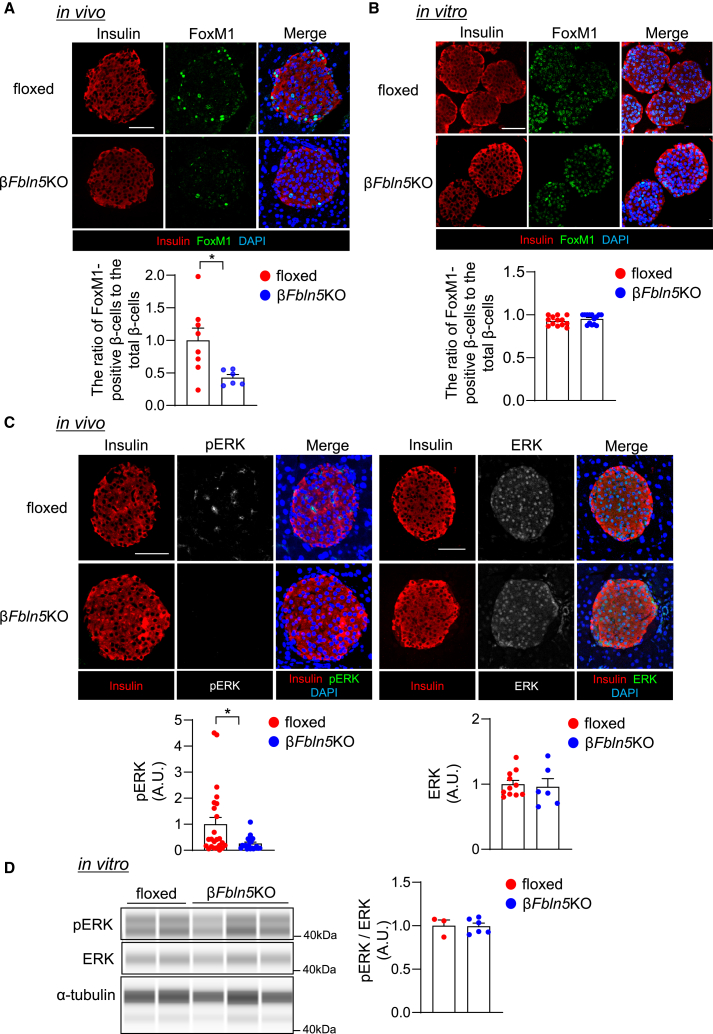
Effects of β-cell-derived Fbln5 on FoxM1 and ERK signaling (A) Upper panel: Representative images of pancreas sections from 11-week-old β*Fbln5*KO and control mice stained with antibodies against FoxM1 (green) and insulin (red) are shown. Nuclei are stained blue with DAPI. The scale bar represents 50 μm. Lower panel: The ratio of FoxM1-positive nuclei in β-cells relative to total β-cells per islet was calculated (n = 6–8 islets per group). The data are presented as the means ± SEMs. ∗*p* < 0.05, Student’s t test. (B) Upper panel: Representative images of islets isolated from 11-week-old β*Fbln5*KO and control mice stained with antibodies against FoxM1 (green) and insulin (red) are shown. Nuclei are stained blue with DAPI. The scale bar represents 50 μm. Lower panel: The ratio of FoxM1-positive nuclei in β-cells relative to total β-cells per islet was calculated (*n* = 20 islets in the floxed group and 14 islets in the β*Fbln5*KO group). The data are presented as the means ± SEMs. (C) Upper panel: Representative image of pancreatic sections from 11-week-old *Fbln5*-floxed and β*Fbln5*KO mice stained with antibodies against phospho-ERK (pERK, gray in the middle panels and green in the right panels), ERK (gray in the middle panels and green in the right panels) and insulin (red) are shown. Nuclei are stained blue with DAPI. The scale bar represents 50 μm. Lower panel: The intensity of pERK (*n* = 24 in the *Fbln5*-floxed group and *n* = 21 in the β*Fbln5*KO group) or ERK (*n* = 11 in the *Fbln5*-floxed group and *n* = 6 in the β*Fbln5*KO group) in the islets of *Fbln5*-floxed and β*Fbln5*KO mice. The data are presented as the means ± SEMs. ∗*p* < 0.05, Student’s t test. (D) Immunoblots showing the levels of phospho-ERK (pERK), ERK and α-tubulin in islets isolated from 11 to 13-week-old β*Fbln5*KO mice and their control littermates. Densitometry data are plotted in the lower graphs. The data are presented as the means ± SEMs.

The transcription factor FoxM1, the transcriptional activity of which is regulated by Chk2 and Cdk1/2 in the nucleus, is thought to activate CENP-A and PLK-1 expression. Since the FoxM1/PLK1/CENP-A pathway is crucial for β-cell proliferation,^27^^,^^28^ we further evaluated the localization of FoxM1 in the β-cells of β*Fbln5*KO and control mice. The fluorescence intensity of FoxM1 in the nucleus of β-cells was decreased by the loss of *Fbln5 in vivo* (Figure 8A). This finding may explain the mechanism underlying the decrease in β-cell proliferation that was observed in the β*Fbln5*KO mice. In contrast, *in vitro* culture of isolated islets revealed similar levels of nuclear FoxM1 between β*Fbln5*KO and control islets (Figure 8B). Since phosphorylated ERK mediates nuclear export of FoxM1,^27^^,^^29^ we examined ERK phosphorylation in the islets of β*Fbln5*KO and control mice (Figures 8C and 8D). The fluorescence intensity of phosphorylated ERK in the islets was attenuated in the β*Fbln5*KO mice *in vivo*, although the ratio of phosphorylated ERK relative to the total ERK protein level was not different between islets isolated from β*Fbln5*KO mice and islets isolated from control mice. These results were consistent with the findings for the effect of β*-*cell-specific *Fbln5* deletion on β-cell proliferation *in vivo* and *in vitro*, indicating the role of the secreted Fbln5 protein in β-cell proliferation.

**Figure 9:**
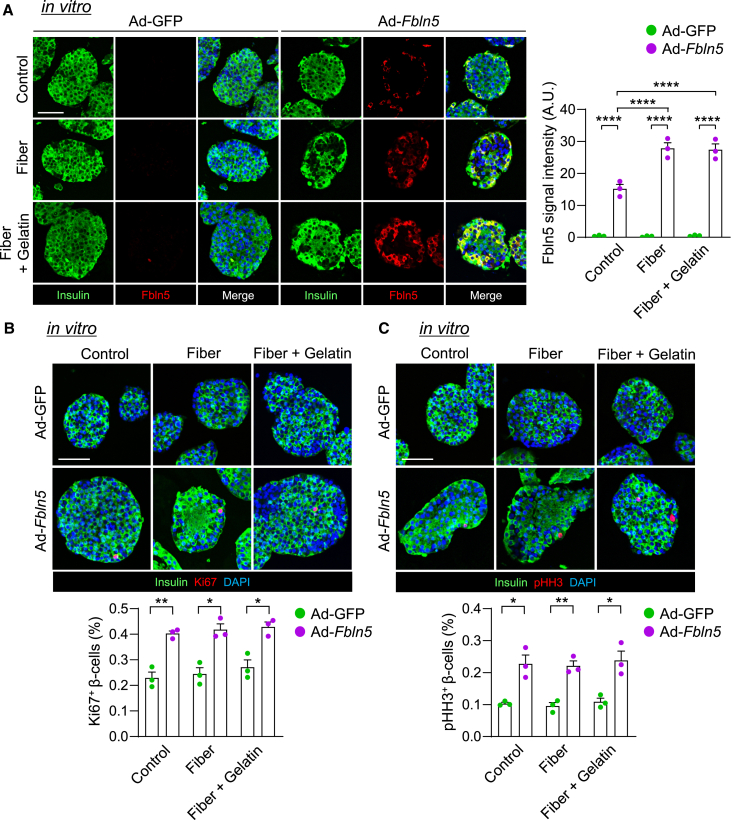
Adenovirus-mediated Fbln5 overexpression in islets increased β-cell proliferation (A) Islets isolated from C57B6/J mice were encapsulated in alginate fibers or fibers plus gelatin 48 h after Ad-*Fbln5* or Ad-GFP (control) infection for 24 h and embedded in agarose gel. Then, the islets were stained with Fbln5 (red) and insulin (green). The signal intensity of Fbln5 is shown in the right graph (*n* = 3 per group). The scale bar represents 50 μm. The data are presented as the means ± SEMs. ∗∗∗∗*p* < 0.0001, one-way ANOVA followed by the Tukey HSD post hoc test. (B) Ki67-positive cells in Ad-*Fbln5*-or Ad-GFP-infected pancreatic islets of C57BL6/J mice. Representative images of pancreatic islets (upper panel) and the percentage of Ki67-positive β-cells among insulin-positive β-cells (lower panel) are shown (*n* = 3 per group). Insulin is stained green, the cell nuclei are stained blue with DAPI, and the Ki67-positive nuclei are stained red. The scale bar represents 50 μm. The data are presented as the means ± SEMs. ∗*p* < 0.05 and ∗∗*p* < 0.01, one-way ANOVA followed by the Tukey HSD post hoc test. (C) pHH3-positive cells in Ad-*Fbln5*-or Ad-GFP-infected pancreatic islets of C57BL6/J mice. Representative images of pancreatic islets (upper panel) and the percentage of Ki67pHH3-positive β-cells among insulin-positive β-cells (lower panel) are shown (*n* = 3 per group). Insulin is stained green, the cell nuclei are stained blue with DAPI, and the pHH3-positive nuclei are stained red. The scale bar represents 50 μm. The data are presented as the means ± SEMs. ∗*p* < 0.05 and ∗∗*p* < 0.01, one-way ANOVA followed by the Tukey HSD post hoc test.

**Figure 10:**
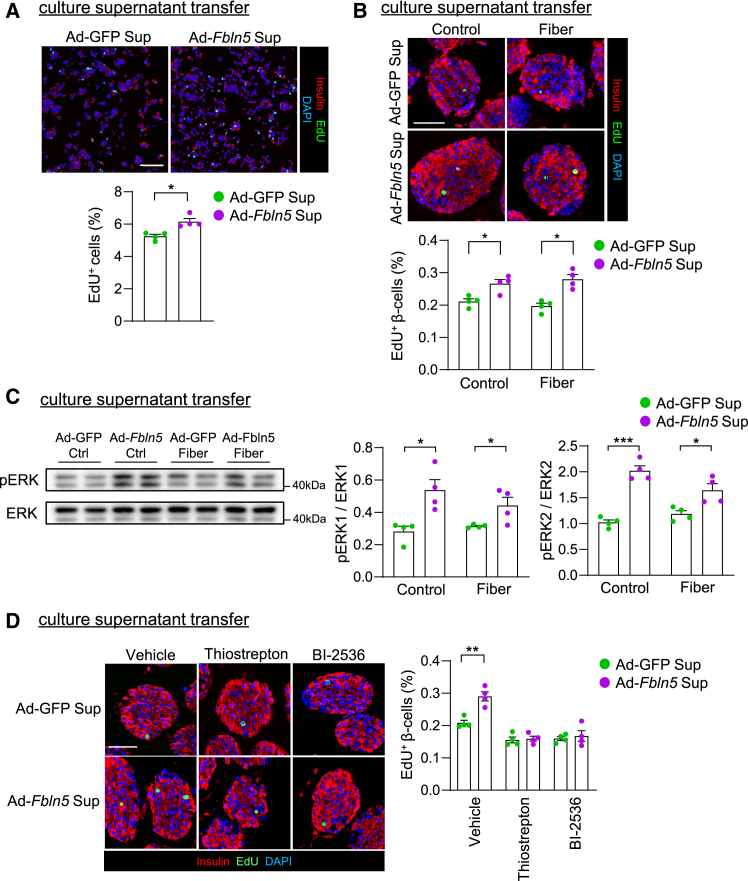
Treatment with culture supernatant containing secreted Fbln5 increased β-cell proliferation and the activity of the FoxM1/PLK1 pathway (A) EdU incorporation in INS-1 cells treated with culture supernatant (Sup) collected from Ad-GFP- or Ad-*Fbln5*-infected INS-1 cells for 72 h. The cells were incubated for 4 h with 10 μM EdU after 48 h of treatment with the supernatant (*n* = 4 per group). The scale bar represents 100 μm. The data are presented as the means ± SEMs. ∗*p* < 0.05, Student’s t test. (B) EdU incorporation in islets treated with culture supernatant collected from Ad-GFP- or Ad-*Fbln5*-infected islets for 48 h. Islets were incubated for 4 h with 10 μM EdU after 48 h of treatment with the supernatant with or without fibers (*n* = 4 per group). The scale bar represents 50 μm. The data are presented as the means ± SEMs. ∗*p* < 0.05, one-way ANOVA followed by the Tukey HSD post hoc test. (C) Immunoblots showing the levels of phospho-ERK (pERK) and ERK in isolated islets treated with culture supernatant collected from Ad-GFP- or Ad-*Fbln5*-infected islets for 48 h, with or without fibers (*n* = 4 per group). Densitometry data are plotted in the right graphs. The data are presented as the means ± SEMs. ∗*p* < 0.05, ∗∗∗*p* < 0.001, one-way ANOVA followed by the Tukey HSD post hoc test. (D) Mouse islets were treated with supernatant containing 10 μM thiostrepton or 50 nM BI-2536. Left panel: Representative images of immunofluorescence staining of mouse islets treated with the supernatant of islets transduced with Ad-GFP or Ad-*Fbln5* containing vehicle (DMSO), 10 μM thiostrepton, or 50 nM BI-2536 for 48 h. Right panel: Proportion of EdU-positive β-cells (*n* = 4 per group). Scale bar: 50 μm. The data are presented as the means ± SEMs. ∗∗*p* < 0.01, one-way ANOVA followed by the Tukey HSD post hoc test.

Next, we investigated β-cell proliferation in *Fbln5*-overexpressing islets with an *Fbln5*-expressing adenovirus (Ad-*Fbln5*). Although *Fbln5* mRNA expression was significantly elevated in Ad-*Fbln5*-infected islets compared with Ad-GFP-infected control islets under normal culture conditions, encapsulation in alginate fibers or fibers with gelatin powder, which resembled the reconstituted extracellular matrix, further increased *Fbln5* expression in the islets (Figure 9A). The proportion of Ki67-positive or pHH3-positive proliferating β-cells was increased in the *Fbln5*-overexpressing islets under each condition (Figures 9B and 9C). We treated INS-1 cells with culture media from *Fbln5*-expressing INS-1 cells to evaluate the effect of secreted Fbln5 on β-cell proliferation. Compared with Ad-*GFP*-infected INS-1 cells, the proliferation of INS-1 cells treated with the culture supernatant from Ad-*Fbln5*-infected INS-1 cells was increased (Figure 10A). Similarly, the treatment of islets with culture supernatant from Ad-*Fbln5*-infected islets significantly increased β-cell proliferation, and increased ERK phosphorylation was observed in these islets (Figures 10B and 10C). We evaluated the levels of the secreted V5 protein in the culture supernatant of islets infected with V5-tagged Fbln5-expressing adenovirus via western blotting to confirm the secretion of Fbln5 into culture media (Figure S13A). Integrin-linked kinase (ILK) and focal adhesion kinase (FAK) are known to mediate survival, differentiation, proliferation, apoptosis, and gene expression in integrin signaling.^30^^,^^31^^,^^32^ Therefore, we evaluated the effects of Fbln5 on the ILK and FAK pathways via integrin signaling. Treatment with the culture supernatant from Ad-*Fbln5*-infected INS-1 cells did not influence the phosphorylation of FAK or the expression of ILK1 (Figure S13B). BI-2536, a PLK-1 inhibitor, attenuated the proliferation and viability of INS-1 cells treated with culture supernatant from *Fbln5*-overexpressing INS-1 cells (Figures S13C and S13D). Increased β-cell proliferation in islets treated with media containing secreted Fbln5 was also attenuated by the presence of BI-2536 or thiostrepton, a FoxM1 inhibitor (Figure 10D). These results support our hypothesis that secreted Fbln5 induces β-cell proliferation through the FoxM1/PLK1/CENP-A pathway.

## Discussion

The ECM is essential for cell–matrix signaling and cell–cell signaling, and the importance of cell–matrix contact in cell survival and cell proliferation has been established.^33^ Collagen IV, laminin, fibronectin, and heparan sulfate, but not Fbln5, are common ECM components in the islet vascular basement membrane. These common ECM components modulate cell adhesion, migration, and insulin secretion in islets, partly through interactions with β1 integrin.^1^ Few reports have described the roles of ECM proteins in the regulation of β-cell function in response to glucose. Most previous studies have assessed the effects of the ECM on β-cells *in vitro*^34^ but not sufficiently *in vivo*. These findings prompted us to assess the role of Fbln5 in the regulation of β-cell proliferation *in vivo*.

*Ins1*-Cre-mediated *Fbln5* deletion in β-cells resulted in a significant reduction in *Fbln5* expression in islets. Single-cell RNA-Seq also suggested that the proportion of Fbln5-expressing β-cells was increased when the mice were fed a high-fat diet. Another study revealed that the ablation of the β1 integrin receptor in pancreatic β-cells inhibited the expression of cell cycle-regulating genes and β-cell expansion without causing glucose intolerance or reducing insulin secretion in response to glucose.^35^ The phenotypes of β*Fbln5*KO mice closely resemble those of mice with genetic β1 integrin ablation in β-cells. Thus, ECM proteins are essential for β-cell expansion, and these results imply that β-cell-derived Fbln5 plays essential roles in the appropriate maintenance of β-cell proliferation.

Our results raise the possibility that the interaction of Fbln5 with surrounding tissues is required for the regulation of β-cell proliferation. In fact, treatment with culture media from *Fbln5*-overexpressing INS-1 cells or islets significantly increased β-cell proliferation. Fbln5 may behave differently depending on where it is located in the context of cell proliferation. Thus, our data suggest that Fbln5 secreted from β-cells in response to ambient glucose increases β-cell proliferation via autocrine or paracrine actions with the ECM.

Fbln5 has an integrin-binding RGD motif to bind integrin family proteins and integrins that regulate intracellular signaling, including ERK and focal adhesion kinase (FAK), through cell adhesion to the ECM.^19^^,^^36^ Fbln5 has also been shown to be induced by transforming growth factor β (TGF-β) and to affect TGF-β-mediated ERK and p38 MAPK activation.^21^ Indeed, in this study, decreased ERK phosphorylation in the islets of β*Fbln5*KO mice was observed compared with that in the control islets *in vivo*. This difference in ERK phosphorylation between β*Fbln5*KO mice and control mice was abolished by the isolation of islets *in vitro*. These findings further substantiate the notion that Fbln5, when secreted from β-cells, may stimulate cell proliferation through interactions at the extracellular–cell interface or through cell adhesion. In addition, because Fbln5 regulates integrin-induced ROS production by binding to β1 integrin, it might be associated with angiogenesis and cell proliferation.^37^

In the analysis of gene expression using a microarray, genes encoding members of the solute carrier family were differentially expressed between the β*Fbln5*KO islets and the control islets. *Fbln5* deletion in β-cells upregulated *slc38a1* but downregulated *slc5a2, slc23a2*, and *slc43a1*. Members of the solute-carrier protein family reportedly play various roles in β-cell function or dysfunction during the development of diabetes.^38^^,^^39^^,^^40^^,^^41^^,^^42^ We also identified 6 genes encoding olfactory receptors among the 20 genes with the most upregulated expression in β*Fbln5*KO islets. Recently, the expression of olfactory receptors in pancreatic β-cells and their roles in the promotion of glucose-induced insulin secretion have been reported.^43^ Hence, these molecules are possibly associated with β-cell proliferation via Fbln5.

A functional GO analysis of the downregulated genes in β*Fbln5*KO islets suggested the association of Fbln5 with cell proliferation, such as epithelial cell development and the regulation of PLK1 activity at the G2/M transition. Indeed, incubation with a PLK1 inhibitor decreased β-cell proliferation induced by β-cell-secreted Fbln5. PLK1 is reportedly necessary for adaptive β-cell proliferation through the FoxM1/PLK1/CENP-A pathway in mitotic cell cycle progression.^27^ We observed a decrease in the nuclear localization of FoxM1 in *Fbln5*-deficient β-cells compared with that in control cells and an attenuated intensity of phosphorylated ERK in β*Fbln5*KO islets. Furthermore, treatment with a FoxM1 inhibitor attenuated β-cell proliferation induced by secreted Fbln5. Previous studies have shown that ERK is activated by Fbln5^21^^,^^44^; additionally, the inhibition of ERK phosphorylation by Fbln5 has also been demonstrated.^18^^,^^45^ Thus, our data suggest that Fbln5 may activate ERK to enhance FoxM1-mediated proliferative signaling in β-cells *in vivo.* Taken together, these findings indicate that β-cell-derived Fbln5 likely plays a pivotal role in β-cell proliferation through multiple pathways, including cell attachment and/or regulation of the cell cycle.

**Figure 11:**
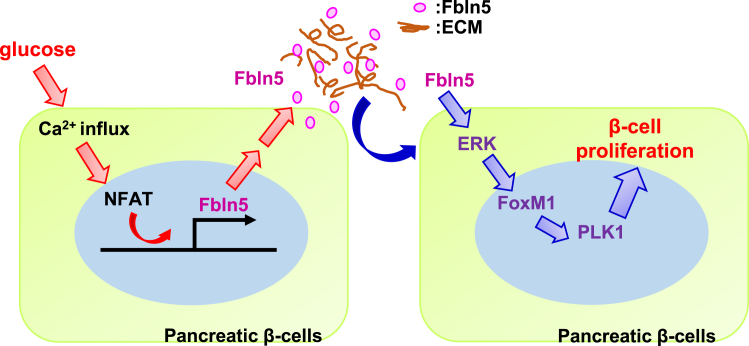
Schematic representation of the role of Fbln5 in the regulation of β-cell proliferation Fbln5 is secreted from β-cells in response to glucose and plays important roles in the appropriate maintenance of β-cell proliferation through an interaction with the extracellular matrix.

In summary, we documented the expression and induction of Fbln5 in β-cells in response to glucose and its important role in appropriate β-cell proliferation in cooperation with the extracellular surroundings *in vivo* (Figure 11). Since Fbln5 is a promising mediator of glucose signaling, the regulation of Fbln5 might be a novel approach for protecting β-cell functions.

### Limitations of the study

The current study has several limitations. Although we evaluated the effects of β-cell-specific *Fbln5* deletion on mice fed normal chow and a high-fat diet for 12 weeks, the use of longer periods of high-fat diet feeding or other diabetic or obese models may be appropriate for assessing the function of β-cell-derived Fbln5. We previously reported that Fbln5 expression in islets was downregulated in an age-dependent manner.^5^ Since we performed all experiments with male mice, the influence of sex is not confirmed. Studies comparing the effects of Fbln5 at different ages are warranted. Although we performed *in vitro* islet experiments, one must also consider the potential of the intraislet ECM or nonβ islet cells to impact β-cell functionality. Because some humoral factors exert dose-dependent effects, experiments with multiple degrees of adenoviral overexpression would be informative. A comprehensive analysis using diabetic and nondiabetic human islets, including studies of the β-cell proliferative pathway coupled with adenoviral Fbln5 overexpression, might reveal the molecular mechanisms underlying Fbln5-mediated human β-cell proliferation.

## Resource availability

### Lead contact

Further information and requests for resources and reagents should be directed to and will be fulfilled by the Lead Contact, Jun Shirakawa MD, PhD (jshira@gunma-u.ac.jp).

### Material availability

This study did not generate new unique reagents.

### Data and code availability

• Microarray data for β*Fbln5*KO islets have been deposited in GEO. The accession number is listed in the key resources table. All data reported in this article will be shared by the lead contact upon request.
• All codes used for analyzing the single-cell RNA-seq data are reposited at GitHub (https://github.com/ShirakawaLab/Fbln5_2025).
• Any additional *information* required to reanalyze the data reported in this article is available from the lead contact upon request.

## Acknowledgments

We thank Mitsuyo Kaji and Eri Sakamoto (Yokohama City University) for their technical assistance and Fuyumi Murai (Gunma University) and Misa Katayama (Yokohama City University) for their secretarial assistance. J.S. acknowledges support from the JST FOREST Program, 10.13039/501100001700MEXT of Japan (a Grant-in-Aid for Scientific Research (B) 23H03324), the 10.13039/501100004014Japan IDDM network, the 10.13039/100007449Takeda Science Foundation, the 10.13039/100016289Taiju Life Social Welfare Foundation, the 10.13039/501100012627Japan Diabetes Foundation supported by Nippon Boehringer Ingelheim Co.,Ltd., the 10.13039/100008732Uehara Memorial Foundation, the 10.13039/100007428Naito Foundation, the 10.13039/100007434Suzuken Memorial Foundation, the 10.13039/501100008644Japan Diabetes Society Carrier Development Award supported by 10.13039/100015990Sanofi, the Daiichi Sankyo Foundation of Life Science, 10.13039/100012260Calgary Foundation for Innovative Drug Discovery Science, and 10.13039/501100007664Manpei Suzuki Diabetes Foundation. This work was also supported by a Grant-in-Aid for Scientific Research (C) 22K08629 from 10.13039/501100001700MEXT of Japan and the Foundation for Applied Enzymology (FFDR) (to T.O).

## Author contributions

J.S. designed the study. T.O. and J.S. performed the experiments, analyzed the data, and wrote and edited the article. H.Y provided the Fbln5-floxed mice and contributed to the discussion. T.K. and A.M.J.S. contributed to providing human islets. T.T., R.I., S.F., M.K., A.M., D.M., K.N., Y.Ta., Y.To., and Y.Te. assisted with the experiments and contributed to the discussion. All the authors approved the final version of the article. J.S. is the guarantor of this work and, as such, has full access to all the data in the study and takes responsibility for the integrity of the data and the accuracy of the data analysis.

## Declaration of interests

The authors have no competing financial interests to declare.

## STAR★Methods

### Key resources table

**Table:** 

REAGENT or RESOURCE	SOURCE	IDENTIFIER
**Antibodies**		

Alpha tubulin antibody [DM1A]	Abcam	Cat# ab7291; RRID: AB_2241126
Insulin (H-86) antibody	Santa Cruz Biotechnology	Cat# sc-9168; RRID: AB_2126540
Anti-insulin antibody [EPR17359]]	Abcam	Cat# ab181547; RRID:AB_2716761
Anti-glucagon antibody	Abcam	Cat# ab10988; RRID:AB_297642
rabbit polyclonal anti-fibulin-5 (BSYN 1923	Laboratory of H. Yanagisawa (Yanagisawa et al., 2002)^13^	N/A
Fibulin 5 Polyclonal antibody	Proteintech	Cat#12188-1-AP; RRID: AB_2105939
p44/42 MAPK (Erk1/2) Antibody	Cell Signaling Technology	Cat# 9102; RRID:AB_330744
phospho p44/42 MAPK (Erk1/2) Antibody	Cell Signaling Technology	Cat# 4370; RRID:AB_2315112
FOXM1 (C-20) antibody	Santa Cruz Biotechnology	Cat# sc-502; RRID:AB_631523
anti-bromodeoxyuridine, anti-BrdU antibody	Dako	Cat# M0744; RRID: AB_10013660
Purified Mouse Anti-Ki-67	BD Bioscience	Cat# 556003; RRID: AB_396287
Anti-phospho-Histone H3 (Ser10)	Millipore	Cat# 06-570; RRID:AB_310177
pFAK	Cell Signaling Technology	Cat# 3283; RRID: AB_2173659
FAK	Santa Cruz	Cat# sc-271126; RRID: AB_10614323
ILK	Cell Signaling Technology	Cat# 3856; RRID: AB_ 2233861
GAPDH	ell Signaling Technology	Cat# 5174; RRID: AB_ 10622025
Purified anti-mouse/rat CD29	BioLegend	Cat# 102201; RRID:AB_312879
Laminin β-1 (LT3)	Santa Cruz	Cat# sc-33709; RRID: AB_627868
CD31	BioLegend	Cat# 102501; RRID: AB_ 312908
Donkey Anti-Goat IgG H&L (Alexa Fluor 488)	Abcam	Cat# ab150129; RRID: AB_2687506
Rat Anti-Histone H3, phospho (Ser28) Monoclonal Antibody, Alexa Fluor 647 Conjugated	BD Bioscience	Cat# 558217; RRID: AB_397065
Donkey anti-Goat IgG (H + L) Cross-Adsorbed Secondary Antibody, Alexa Fluor 647	Invitrogen	Cat# A-21447; RRID: AB_141844
Donkey anti-Rabbit IgG (H + L) Highly Cross-Adsorbed Secondary Antibody, Alexa Fluor 488	Invitrogen	Cat# A-21206; RRID: AB_141708
Donkey anti-Rabbit IgG (H + L) Highly Cross-Adsorbed Secondary Antibody, Alexa Fluor 555	Invitrogen	Cat# A-31572; RRID: AB_162543
mouse anti-Armenian Hamster IgG-FITC	Santa Cruz	Cat# sc-2792; RRID: AB_628483

**Bacterial and virus strains**		

Ad-Fbln5	Laboratory of Y. Terauchi (Okuyama et al., 2017)^5^	N/A

**Biological samples**		

Human islets, see Table S1		

**Chemicals, peptides, and recombinant proteins**		

RPMI 1640(No Glucose) with L-Gln, liquid	Nacalai tesque, Kyoto, Japan	Cat# 09892-15
D-PBS(-) without Ca and Mg, liquid	Nacalai tesque, Kyoto, Japan	Cat# 14249-95
Agilent Seahorse XF RPMI Medium (without Phenol Red)	Agilent Technologies	Cat# 103336-100
Final Wash/Culture Medium	Mediatech	Cat# 99-785-CV
45(w/v)%-D-(+)-Glucose Solution	Nacalai tesque, Kyoto, Japan	Cat# G8769
Opti-MEM	Thermo Fisher Scientific	Cat# 31985062
100mM-Sodium Pyruvate Solution(100x)	Nacalai tesque, Kyoto, Japan	Cat# 06977-34
Penicillin-Streptomycin Mixed Solution	Nacalai tesque, Kyoto, Japan	Cat# 26253-84
200mmol/l L-Alanyl-L-glutamine Solution(100x)	Nacalai tesque, Kyoto, Japan	Cat# 04260-64
1mol/L-HEPES Buffer Solution	Nacalai tesque, Kyoto, Japan	Cat# 17557-94
2-Mercaptoethanol	Nacalai tesque, Kyoto, Japan	Cat# 21417-52
Poly-L-lysine solution	Sigma-Aldrich	Cat# 21417-52
Protease Inhibitor Cocktail (EDTA free) (100x)	Nacalai tesque, Kyoto, Japan	Cat# 03969-21
Phosphatase Inhibitor Cocktail (EDTA free) (100x)	Nacalai tesque, Kyoto, Japan	Cat# 07575-51
Insulin solution human	Sigma-Aldrich	Cat# I9278
Protein Assay BCA Kit	Nacalai tesque, Kyoto, Japan	Cat# 06385-00
SYBR Green Supermix	Bio-Rad	Cat# 1725274
Liraglutide	Novo Nordisk	N/A
forskolin	Tokyo Chemical Industry	Cat# F0855
H-89	MedChemExpress	Cat# HY-15979
ESI-09	MedChemExpress	Cat# HY-16704
RNeasy Mini Kit	QIAGEN	Cat# 74106
High-Capacity cDNA Reverse Transcription Kit	Thermo Fisher Scientific	Cat# 4368813
DAPI solution	Wako Pure Chemical Industries	Cat# 340-07971
Fluoro-KEEPER Antifade Reagent, Non-Hardening Type	Nacalai tesque, Kyoto, Japan	Cat# 12593-64
BI-2536	Santa Cruz Biotechnology	Cat# sc-364431
thiostrepton	MedChemExpress	Cat# HY-B0990

**Critical commercial assays**		

Glutest Neo Super	Sanwa Chemical Co. Kanagawa, Japan	N/A
Mouse/Rat Insulin ELISA kit	Morinaga	Cat# M1108
VECTASTAIN Elite ABC HRP Kit	Vector Laboratories	Cat# PK-6101
DAB Peroxidase (HRP) Substrate Kit	Vector Laboratories	Cat# SK-4100
Click-iT Plus EdU Alexa Fluor 488 Imaging Kit	Thermo Fisher Scientific	Cat# C10637
CellTiter 96® Non-Radioactive Cell Proliferation Assay (MTT)	Promega	Cat# G4001
Seahorse XFe96 Flux Pak	Agilent Technologies	Cat# 102416-100

**Deposited data**		

Gene expression profiling in islets from βFbln5KO mice by array	This paper	GEO: https://www.ncbi.nlm.nih.gov/geo/query/acc.cgi?acc=GSE143633
Single-cell RNA-seq data	Fu et al.^22^	GEO: https://www.ncbi.nlm.nih.gov/geo/query/acc.cgi?acc=GSE203376
Single-cell RNA-seq data	Oppenländer et al.^23^	GEO: https://www.ncbi.nlm.nih.gov/geo/query/acc.cgi?acc=GSE174194

**Experimental models: Cell lines**		

INS-1 832/13 β-cells	Laboratory of Christopher Newgard (Hohmeier et al., 2000)^46^	N/A

**Experimental models: Organisms/strains**		

*Fbln5*KO mice	Laboratory of H. Yanagisawa (Yanagisawa et al., 2002)^13^	N/A
β*Fbln5*KO mice	This paper	N/A
*Fbln5* floxed mice	Laboratory of H. Yanagisawa (Yanagisawa et al., 2016)^47^	N/A

**Oligonucleotides**		

Primers for qPCR, see Table S1		

**Software and algorithms**		

Prism 8 software (GraphPad Software)	Graph Pad Software	https://www.graphpad.com/scientific-software/prism/
ImageJ software	NIH	https://imagej.net.
Seurat (version 4.3.0)	Stuart, T. et al.	https://satijalab.org/seurat/
R (version 4.2.2)	R CRAN	https://cran.r-project.org/
Codes for single-cell RNA-seq analysis	This paper	https://github.com/ShirakawaLab/Fbln5_2025
Metascape	Zhou, Y. et al.	https://metascape.org/gp/index.html#/main/step1

### Experimental model and study participant details

#### Mice

We generated β*Fbln5*KO mice by crossing *Fbln5*-floxed mice with *Ins1*-Cre mice. The *Fbln5*-floxed mouse carrying Fbln5, in which exons 1a and 1b are flanked by two loxP sites, has been described previously.^47^ The *Ins1*-Cre mouse, which expresses Cre recombinase driven by the mouse insulin 1 promoter (BAC Ins1-cre25), was obtained from Dr. Ken-ichi Yagami, University of Tsukuba, Japan.^48^ The genomic recombination map is shown in Figure 1A. We backcrossed *Fbln5* knockout mice (*Fbln5*KO)^13^ with C57BL/6J mice more than 10 times. All the experiments were conducted on male littermates. This study was conducted with the approval of the Animal Care Committee of Yokohama City University (permit no. F-A-22–016). All the animal procedures were performed in accordance with the institutional animal care guidelines and the guidelines of the Animal Care Committee of Yokohama City University. The animal housing rooms were maintained at a constant temperature (25°C) under a 12-hour light (7:00)/dark (19:00) cycle.

#### Human islet study

Human islets were obtained from the Clinical Islet Laboratory and the Clinical Islet Transplant Program of the University of Alberta, Canada. The details of the human islets are described in Table S2. We used human islets from 3 non-diabetic donors, individually. The study and its protocol were approved by the Yokohama City University Ethics Board (B171100025) and Gunma University (HS2020-193). The islets were cultured overnight in Miami Media #1A (Cellgro). For glucose stimulation, islets were cultured in final wash/culture medium (Cellgro).

### Method details

#### Drugs

The glucokinase activator (GKA) CpdA [2-amino-5-(4-methyl-4H-(1,2,4)-triazole-3-yl-sulfanyl) N-(4-methyl-thiazole-2-yl) benzamide]^49^ was purchased from Calbiochem (San Diego, CA, USA). Liraglutide was obtained from Novo Nordisk (Bagsvaerd, Denmark). Forskolin, an adenylate cyclase activator, was purchased from Tokyo Chemical Industry (Tokyo, Japan). The PKA inhibitor H-89 and the Epac2 inhibitor ESI-09 were purchased from MedChemExpress (New Jersey, USA) and Cayman Chemical (Michigan, USA), respectively. The PLK1 inhibitor BI-2526 and FoxM1 inhibitor thiostrepton were purchased from MedChemExpress.

#### Real-time PCR

Total RNA was isolated from pancreatic islets and the hypothalamus using an RNase-free DNase and RNeasy Kit (Qiagen, Valencia, CA). cDNA was prepared using high-capacity cDNA reverse-transcription kits (Applied Biosystems). Quantitative PCR was performed using TaqMan Gene Expression Assays (7900 Real-Time PCR System; Applied Biosystems) with THUNDERBIRD SYBR qPCR Mix (TOYOBO). The sequences of primers used were as follows: F, TCGCTATGGTTACTGCCAGCA and R, TTGGCAAGACCTTCCATCGTC for *Fbln5*; F, GGCTGTATTCCCCTCCATCG and R, CCAGTTGGTAACAATGCCATGT for *β-actin*; F, TAAGCCCTGAGCCAATCCATC and R, CATTCCCGGTAAATCCACAAGT for *Drp1*; F, GAGGCAAAGGATGATTCGGCTC and R, CGAATCCTATCATCTTTAGCAAGC for *Tfam*; and F, TGGAAAATGGTTCGAGAGTCAG and R, CATTCCGTCTCTAGGTTAAAGCG for *Opa1*. The details of those primers are shown in Table S1.

#### Immunoblotting and digital capillary western blotting

For immunoblotting, isolated mouse islets or INS-1 cells were lysed in ice-cold RIPA buffer supplemented with protease and phosphatase inhibitor cocktails. The culture media were concentrated by centrifugation using Amicon Ultra2 cartridges. The islets, INS-1 cells, or culture media were subjected to immunoblotting. The primary antibodies used were anti-phospho-extracellular signal-regulated kinase (ERK) 1/2 (Thr202/Tyr204) (#4370, Cell Signaling Technology), anti-ERK1/2 (#4695, Cell Signaling Technology), V5 tag monoclonal (#R96025, Invitrogen) and α-tubulin antibodies (#ab7291, Abcam). Automated quantitative western blotting was performed by Abby (ProteinSimple, San Jose, CA) according to the manufacturer’s instructions. The following primary antibodies were used for the Abby analysis: anti-fibulin 5 polyclonal antibody (#12188-1-AP, Proteintech) and α-tubulin antibody (#ab7291, Abcam).

#### Histological analysis

Stained pancreatic islet sections from postnatal Day 6 and 11-week-old mice were analyzed after fixation and paraffin embedding. Islets isolated from mice and human islets were used after being embedded in agarose for immunostaining. The sections were immunostained with antibodies directed against insulin (#sc-377071, Santa Cruz Biotechnology; #ab181547 and #ab7842, Abcam), glucagon (#ab10988, Abcam), BrdU (#M0744, Dako, Tokyo, Japan), FoxM1 (#ab175798, Abcam), phospho-ERK1/2 (Thr202/Tyr204) (#4370, Cell Signaling Technology), ERK1/2 (#9102, Cell Signaling Technology), Ki67 (#556003, BD Biosciences), phospho-Histone H3 (#06-570, Millipore), rabbit polyclonal anti-fibulin-5 (BSYN 1923), CD31(#102501, BioLegend), integrin β1 (#102201, BioLegend), or Laminin β1 (#sc-33709, Santa-Cruz Biotechnology). The mice were administered an intraperitoneal injection of bromodeoxyuridine (BrdU) (100 mg/kg; Nacalai Tesque, Inc., Kyoto, Japan) 6 hours before they were sacrificed. More than 5 pancreatic tissue sections from each animal were examined to assess the β-cell proliferative activity (more than 30 islets and more than 1000 β-cells per mouse). The Fbln5 signal was enhanced by tyramide signal amplification using a TSA Fluorescein System (Perkin Elmer, NEL741001KT). Alexa Fluor 488-, 555-, and 647-conjugated secondary antibodies (Invitrogen, CA, USA, and Jackson ImmunoResearch, Pennsylvania, USA) were used for the fluorescence microscopy. All the images were acquired using a BZ-9000 microscope (Keyence, Osaka, Japan) or FluoView FV1000-D confocal laser scanning microscope (Olympus, Tokyo, Japan). In the BrdU staining experiment, approximately 50 islets per mouse were analyzed to assess the proportion of nuclei showing positive immunostaining for BrdU among the insulin-positive cells in each mouse.

#### Oral glucose tolerance test

Before the oral glucose tolerance test (OGTT), all the mice were not allowed access to food for 16–18 hours. The mice were subsequently orally loaded with glucose at 1.5 mg/g body weight. The blood glucose levels and the serum insulin levels were determined using Glutest Neo Super (Sanwa Chemical Co. Kanagawa, Japan) and an insulin ELISA kit (Morinaga Institute of Biological Science, Yokohama, Japan), respectively.

#### Glucose-stimulated insulin secretion from isolated islets

Islets were isolated from the mice via a collagenase injection into the common bile duct, as described in a previous report.^50^ Ten islets were isolated from each β*Fbln5*KO and control mouse and subjected to a preincubation at 37°C in Krebs–Ringer bicarbonate buffer containing 3.9 mM glucose for one hour. Then, the islets were stimulated with 10 mM or 16.7 mM glucose, and the secreted insulin levels were measured at 0, 5, 15, 30, and 60 min. In addition, insulin secretion from the islets was assessed in the presence of 3.9 mM or 11.1 mM glucose with or without 100 nM liraglutide. The islets were treated with acidified ethanol to measure their insulin contents. The insulin concentration was measured using an insulin ELISA kit (Morinaga Institute of Biological Science, Yokohama, Japan).

#### Islet respirometry

Islets from 11-week-old β*Fbln5*KO or control mice were incubated overnight with 5.6 mM glucose and then seeded onto an XF96 spheroid plate (20 islets/well) containing warm assay medium (Seahorse XF base medium supplemented with 5.6 mM glucose and 0.1% FBS). Mitochondrial respiration was measured using a Seahorse XF96 extracellular flux analyzer (Agilent Technologies, Santa Clara, CA). The basal respiration of the islets was measured in medium containing 5.6 mM glucose, and the maximal respiration was subsequently measured in medium containing 11.1 mM glucose, 4 μM oligomycin and 1 μM FCCP. The OCRs normalized to the baseline values in each group are shown.

#### Cell culture

INS-1 (832/13) cells^46^ were cultured in RPMI 1640 containing 10 mM HEPES, 11.1 mM glucose, 10% FBS, 1 mM sodium pyruvate, 2 mM L-glutamine, 50 μM 2-mercaptoethanol, 100 units/mL penicillin and 100 μg/mL streptomycin. Mycoplasma contamination was not detected by 16s rRNA-based mycoplasma group-specific PCR. The cells were maintained at 37°C in humidified air containing 5% CO_2_.

#### Encapsulation of islets into alginate fibers

Fifty mouse islets from each sample were handpicked in microcentrifuge tubes, centrifuged at 100 × g for 1 min, and washed with 1 mL of PBS. Ten microliters of 16.7 mg/mL sodium alginate (Mochida Pharmaceutical, ALG100), supplemented with or without 4 mg/mL gelatin tips (NIKKE, GCP-300010), was added to the islets. The islet suspension was injected with 100 mM barium chloride, after which the alginate fibers formed immediately.^51^

#### Adenovirus

*Fbln5*-overexpressing recombinant adenovirus (Ad-*Fbln5*)^52^ and GFP-expressing control adenovirus (Ad-GFP) were used for the experiments at a multiplicity of infection of 50 viruses per cell as previously described.^5^

#### Proliferation assay

Islets isolated from β*Fbln5*KO and control mice were incubated with 10 μM 5-ethynyl-2’-deoxyuridine (EdU; Click-iT EdU; Invitrogen Cat. No. C10637) in the presence or absence of 30 μM GKA. After treatment for 24 hours, sections of the islets were prepared after they had been fixed and embedded in 1% agarose. Images were obtained using a FluoView FV1000-D confocal laser scanning microscope, and the percentage of EdU-positive β-cells per total islet β-cell number was calculated. INS-1 cells were cultured with the supernatant from Ad-GFP- or Ad-*Fbln5*-infected INS-1 cells for 72 hours. Then, the cells were incubated for 4 hours with 10 μM EdU after 48 hours of treatment with the supernatant and fixed. EdU incorporation and detection were performed as described in the manufacturer’s protocol. The images were captured using a FluoView FV1000-D confocal laser scanning microscope. We counted the number of EdU-positive proliferating cells after adjusting the gain of fluorescence to eliminate the difference between the number of GFP-positive cells and the number of GFP-negative cells. For the MTT assay, INS-1 cells were plated in 96-well plates at 4x10^4^ cells/well and cultured with the supernatant from Ad-GFP- or Ad-*Fbln5*-infected INS-1 cells for 72 hours, with or without 50 nM BI-2637. The cell number was determined using the CellTiter 96 Non-Radioactive Cell Proliferation Assay (Promega, G4001) according to the manufacturer’s instructions.

#### Analysis of single-cell RNA sequencing data

All the analyses were performed in RStudio with R (version 4.2.2). Cells with more than 200 genes detected were included in subsequent analyses. Seurat^53^ (version 4.3.0) was used to generate a uniform manifold approximation and projection (UMAP) plot and perform the differential expression analysis. The unique molecular identifier (UMI) count matrix was used as the input, and genes expressed in at least 3 cells were retained. The UMI counts were normalized and scaled by the default parameters. The cells were filtered to exclude low-quality cells/doublets (cells with <200 or >5000 genes and a percentage of mitochondria >20% in the GSE203376 dataset,^22^ and cells with <200 or >6000 genes and a percentage of mitochondria >25% in the GSE174194 dataset^23^). The variable genes were used as inputs for principal component analysis (PCA). The Jackstraw procedure was used with 100 replicates to identify significant principal components (PCs) with strong enrichment of differences to separate the cells. Based on the results of the Jackstraw procedure and elbow plots, significant PCs were selected for UMAP and clustered through the FindClusters function. The cluster marker genes were detected using the FindAllMarkers function. A differential expression analysis was performed using the FindMarkers function. The genes with a log2 fold change > 0.1 or < -0.1 and an adjusted p value < 0.05 were defined as differentially expressed genes. Cell types were annotated based on the expression of known cell type-specific markers: α-cells (Gcg), β-cells (Ins1), δ-cells (Sst), PP-cells (Ppy), and endothelial cells (Pecam1). Pathway enrichment analyses were performed using Metascape (https://doi.org/10.1038/s41467-019-09234-6) with the default parameters. The single-cell RNA-seq data analyzed in this study are available from Gene Expression Omnibus (GEO) with the accession numbers GSE203376^22^ and GSE174194.^23^

#### Microarray analysis

Total RNA was extracted from the islets of 11-week-old β*Fbln5*KO and control mice (150 islets per extraction, n = 4) immediately after islet isolation using the RNeasy Mini Kit (Qiagen, Hilden, Germany). The cDNA microarray was analyzed using the SurePrint G3 Mouse GE Microarray 8x60K Ver. 2.0 (G4858A #74809; Agilent Technologies). The slide was scanned on the Agilent DNA Microarray Scanner (G2539A) using a single color scan setting for 8 × 60k array slides. The scanned image was analyzed using Feature Extraction Software 11.0.1.1 (Agilent), and the default parameters were used to obtain background-subtracted images and spatially detrended processed signal intensities. The data were analyzed using GeneSpring 12.1 software (Agilent). Normalization and quality control were performed in a per-chip and per-spot, intensity-dependent manner. The data files were deposited in the NCBI GEO database (GSE143633).

### Quantification and statistical analysis

The statistical analyses were performed using SPSS Statistics 19 (IBM) or Prism 8 (GraphPad Software, San Diego, CA, USA). All the data are presented as the means ± SEMs and were analyzed using Student’s t test or ANOVA. Differences between two groups were analyzed using Student’s t test. For comparisons among more than two groups, one-way ANOVA was used, followed by the Tukey HSD post hoc test. When the data had unequal variances, we used Welch’s one-way ANOVA, followed by the Games–Howell post hoc test. Differences with p values < 0.05 (∗), < 0.01 (∗∗), < 0.001 (∗∗∗) or < 0.0001 (∗∗∗∗) were considered statistically significant. We described the statistical details of experiments in figure legends.

### Additional resources

Human islet study and its protocol were approved by the Yokohama City University Ethics Board (B171100025) (https://www.yokohama-cu.ac.jp/amedrc/ethics/ethical/fuzoku_optout.html) and Gunma University (HS2020-193) (https://ciru.dept.showa.gunma-u.ac.jp/guidance/storage-sample/pdf/2020-174.pdf,:https://ciru.dept.showa.gunma-u.ac.jp/guidance/storae-sample/pdf/2020-193.pdf).
