# Supplementary material for: The matricellular protein Fibulin-5 regulates β-cell proliferation in an autocrine/paracrine manner
Source: iScience. 2025 Jan 21;28(2):111856. doi: 10.1016/j.isci.2025.111856 (PMC11848788; doi:10.1016/j.isci.2025.111856)

iScience, Volume *28*

Supplemental information

The matricellular protein Fibulin-5 regulates β-cell proliferation in an autocrine/paracrine manner

Tomoko Okuyama, Takahiro Tsuno, Ryota Inoue, Setsuko Fukushima, Mayu Kyohara, Anzu Matsumura, Daisuke Miyashita, Kuniyuki Nishiyama, Yusuke Takano, Yu Togashi, Makiko Meguro-Horike, Shin-ichi Horike, Tatsuya Kin, A.M. James Shapiro, Hiromi Yanagisawa, Yasuo Terauchi, and Jun Shirakawa

Figure S1

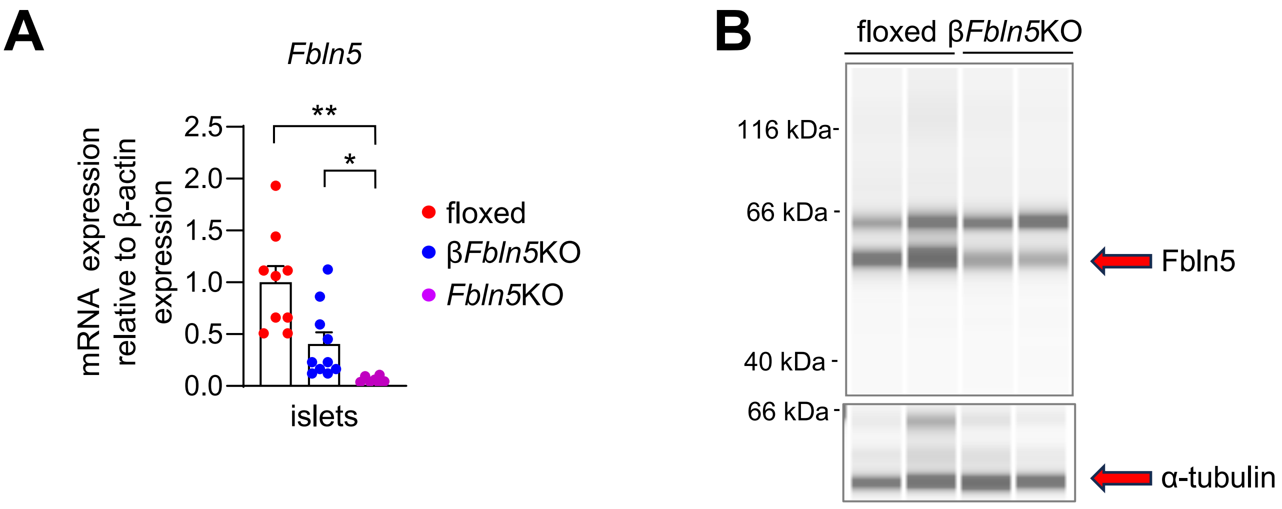

Figure S1. *Fbln5* mRNA expression in islets isolated from *Fbln5*-floxed, β*Fbln5*KO, and *Fbln5*KO mice, related to Figure 1.

(A) *Fbln5* mRNA expression levels in islets isolated from *Fbln5*-floxed (control), β*Fbln5*KO, and *Fbln5*KO mice (n = 10 mice in the control group, n = 11 mice in the β*Fbln5*KO group, and n = 7 mice in the *Fbln5*KO group). The data are presented as the means ± SEMs. *$p < 0.05$ and **$p < 0.01$; one-way ANOVA followed by the Tukey HSD post hoc test. (B) Uncropped image of the immunoblot showing Fbln5 levels in islets isolated from 11-week-old β*Fbln5*KO mice and their control littermates (related to Figure 1E).

Figure S2

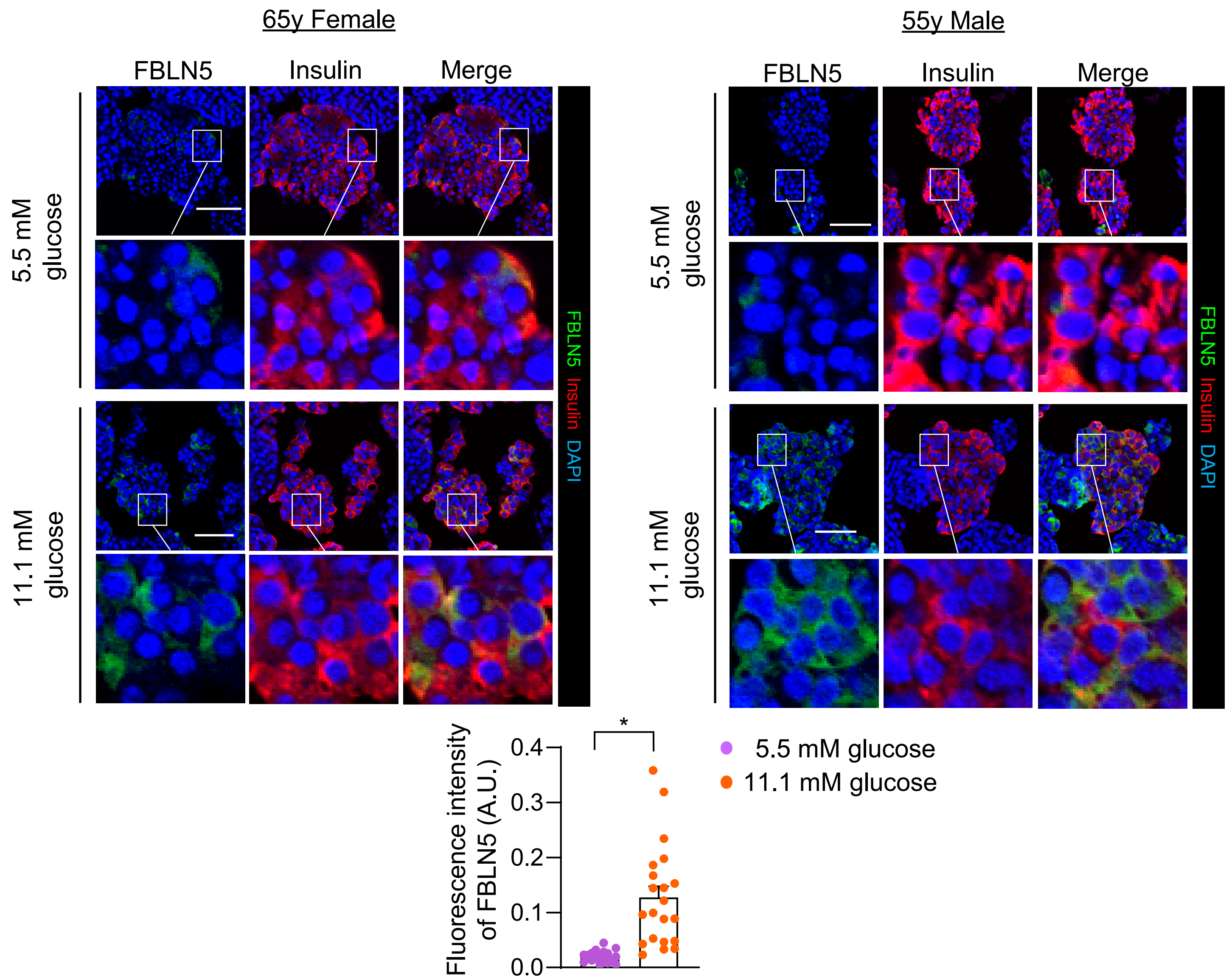

Figure S2. FBLN5 expression in human islets and Fbln5 localization in islets, related to Figure 2.
Representative images of human islet sections stained with antibodies against FBLN5 (green) and insulin (red) are shown. Nuclei are stained blue with DAPI. The scale bar represents 50 μm. The left panel and the right panel show other donors. Islets were treated for 24 hours with 5.5 mM glucose or 11.1 mM glucose. The fluorescence intensity of FBLN5 was calculated in 5 randomly selected areas per islet, and the normalized intensity of insulin is shown in the graph. The data are presented as the means ± SEMs. *$p < 0.05$, Student's t test.

Figure S3

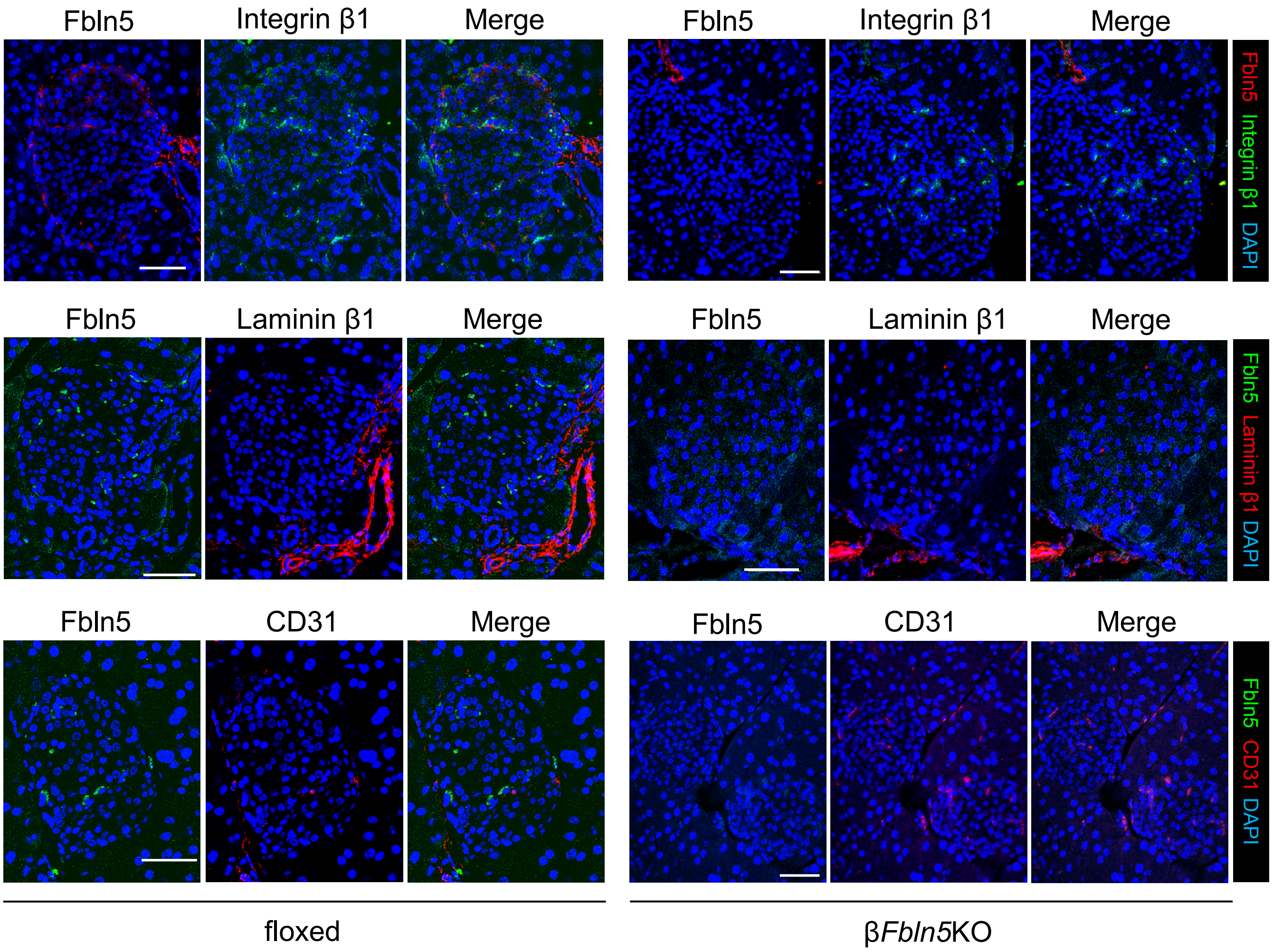

Figure S3. The expression of Fbln5 and ECM markers in the pancreas and islets in immunostaining, related to Figure 2.
Representative images of mouse pancreatic sections stained with antibodies against Fbln5 and integrin β1, Laminin β1, and CD31. Fbln5 is stained red in the upper panel and green in the middle and lower panels. Integrin β1 is stained green in the upper panel, Laminin β1 is stained red in the middle panel, and CD31 is stained red in the lower panel. The scale bar represents 50 μm.

Figure S4

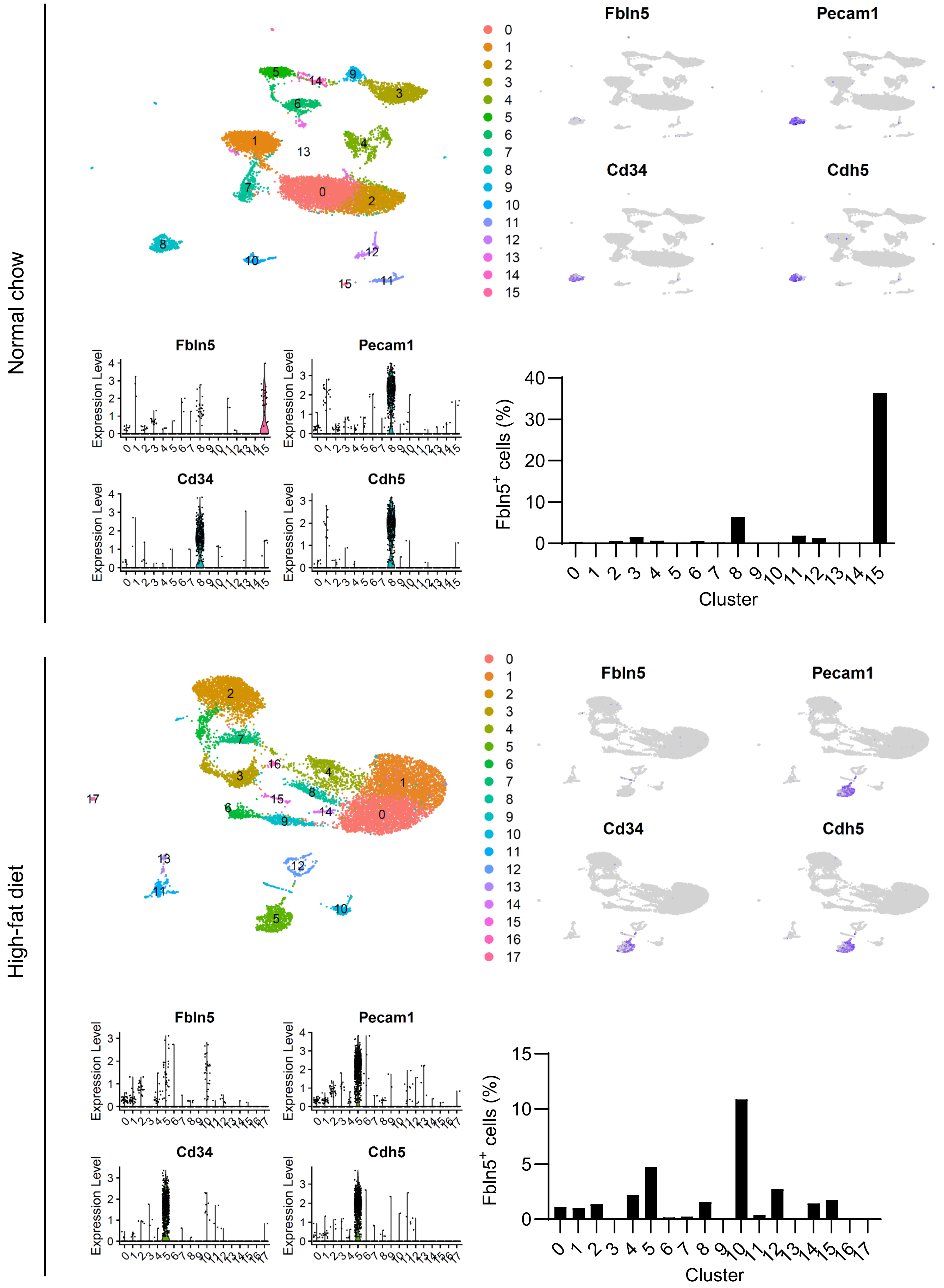

Figure S4. The expression of Fbln5 and ECM markers in the pancreas and islets in the data sets of single-cell RNA-seq, related to Figure 3.
Feature plot and violin plot of CD31 (pecam1), CD34, cadherin5 (cdh5) and Fbln5 expression in the single-cell RNA sequencing datasets of each cell type in islets from normal chow-fed mice (upper panel) and high-fat diet-fed mice (lower panel).

Figure S5

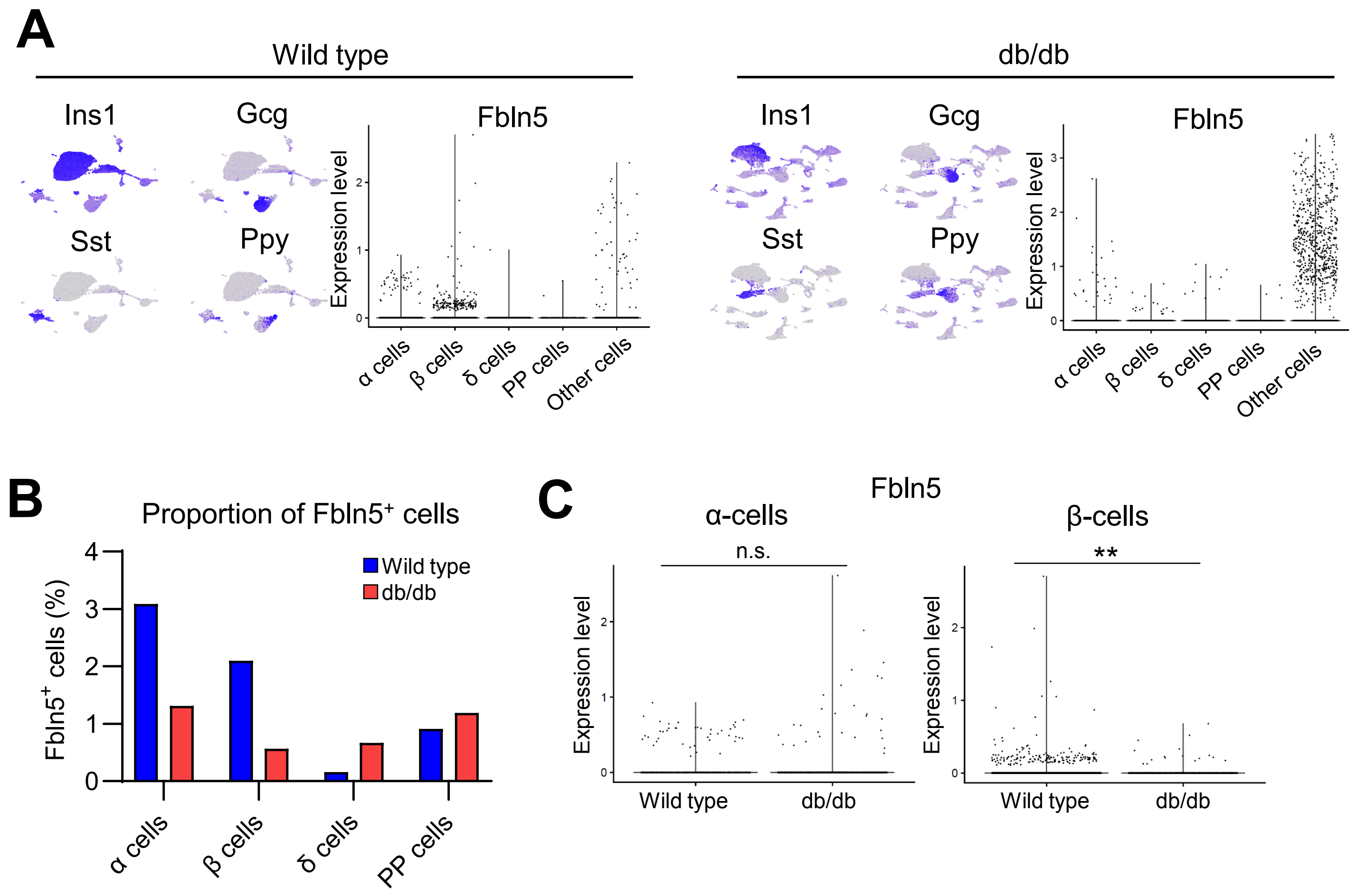

Figure S5. Fbln5 expression in islets from db/db mice, related to Figure 3.
(A) Feature plot and violin plot of Fbln5 and pancreatic endocrine marker data from the single-cell RNA sequencing analysis of islets from db/db mice and wild-type mice. (B) Proportion of Fbln5-positive cells in each pancreatic endocrine cell population. (C) Fbln5 expression levels in α-cells and β-cells. **$p < 0.01$, Student's t test.

Figure S6

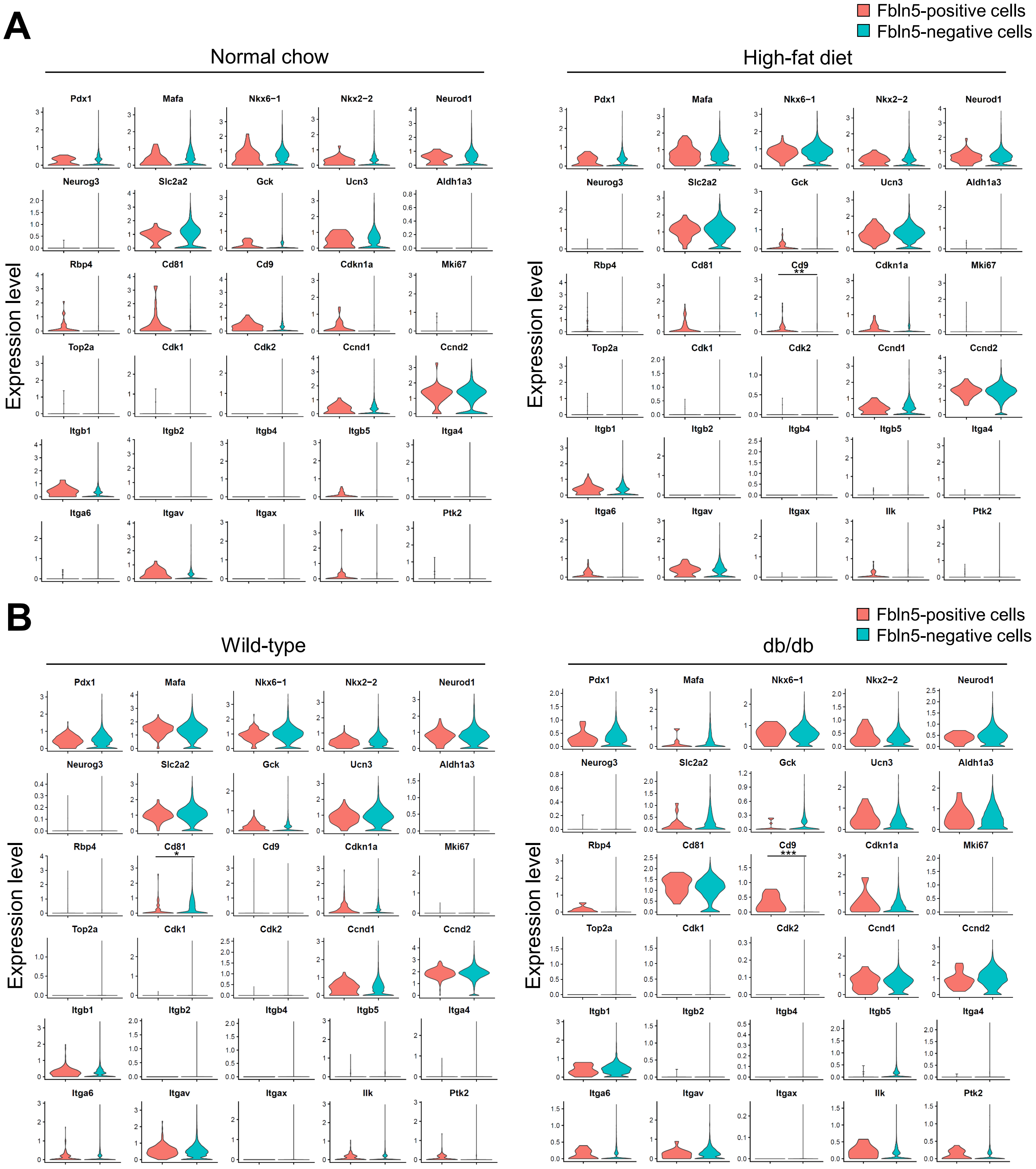

Figure S6. The expression of endocrine maturation markers and ECM markers in Fbln5-positive and Fbln5-negative cells, related to Figure 3.
(A) Violin plot of maturation markers and ECM markers between Fbln5-positive and Fbln5-negative cells from the single-cell RNA sequencing datasets of the pancreatic islets from high-fat diet-fed mice and normal chow-fed mice. (B) Violin plot of maturation markers and ECM markers between Fbln5-positive and Fbln5-negative cells from the single-cell RNA sequencing datasets of the pancreatic islets from db/db mice and wild-type mice.

Figure S7

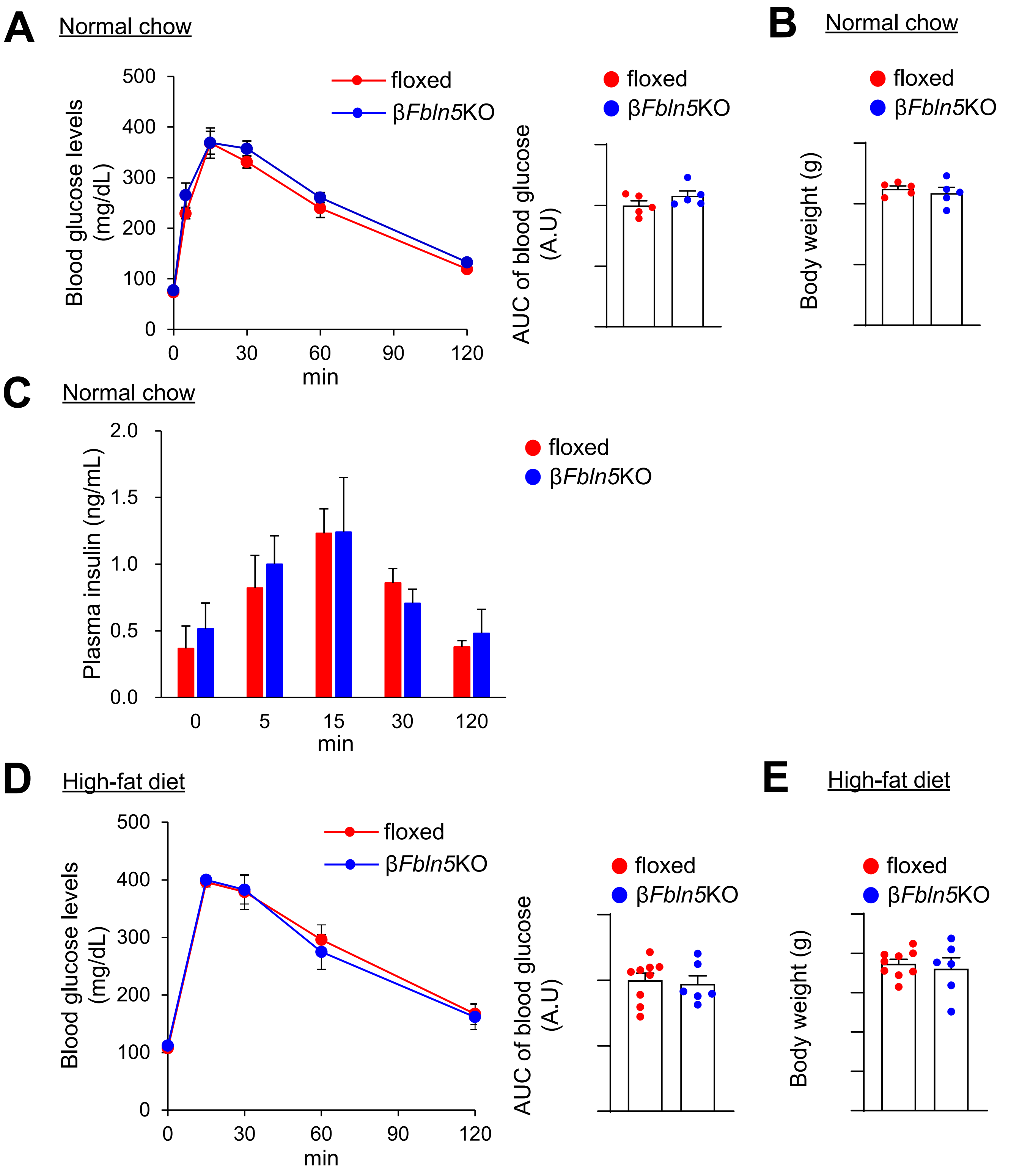

Figure S7. Compared with control mice, adult β*Fbln5*KO mice presented comparable first- and second-phase insulin secretion during an OGTT and glucose tolerance to a high-fat diet, related to Figure 4.
(A) Blood glucose levels during an oral glucose tolerance test (0, 5, 15, 30, 60, and 120 min) performed on 8- to 12-week-old β*Fbln5*KO mice and control mice (n = 5 mice per group). The AUC of blood glucose is shown in the right graph. The data are presented as the means ± SEMs. (B) Body weight gains of 8- to 12-week-old β*Fbln5*KO mice and control mice (n = 5 mice per group). The data are presented as the means ± SEMs. (C) Plasma insulin levels during the oral glucose tolerance test (n = 5 mice per group). The data are presented as the means ± SEMs. (D) Blood glucose levels during an oral glucose tolerance test (0, 15, 30, 60, and 120 min) performed at 12 weeks in high-fat diet-fed β*Fbln5*KO mice and control mice (n = 6–9 mice per group). The AUC of blood glucose is shown in the right graph. The data are presented as the means ± SEMs. (E) Body weight gains of 12-week-old high-fat diet-fed β*Fbln5*KO mice and control mice (n = 6–9 mice per group). The data are presented as the means ± SEMs.

Figure S8

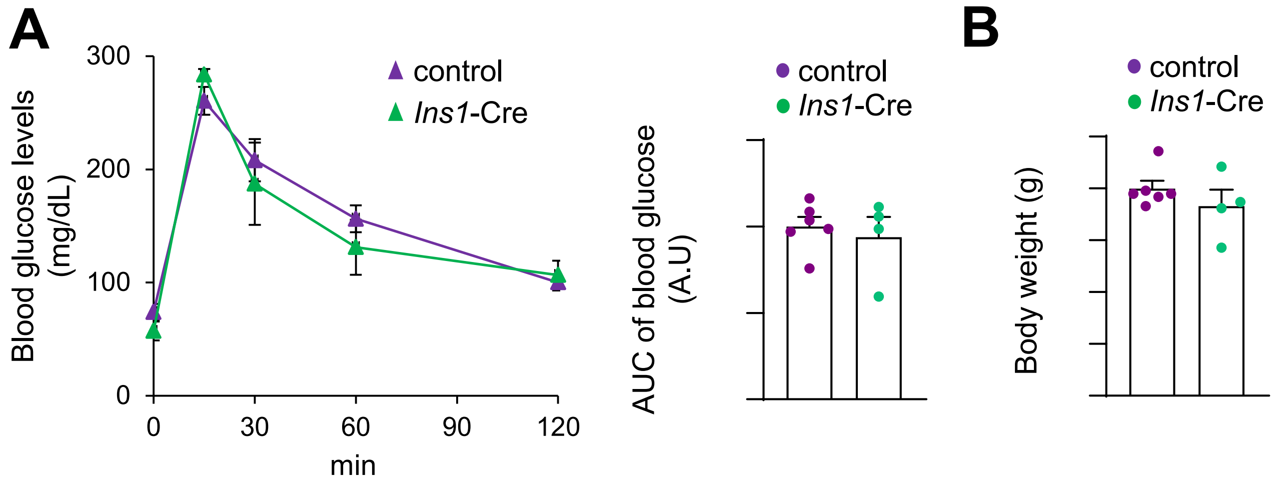

Figure S8. Compared with wild-type mice, *Ins1*-Cre mice did not exhibit glucose intolerance, related to Figure 4.

(A) Blood glucose levels during an oral glucose tolerance test performed in 6- to 7-week-old *Ins1*-Cre mice and control mice (n = 4 *Ins1*-Cre mice, n = 6 control mice). The AUC is shown in the right graph. The data are presented as the means ± SEMs. (B) Body weight gains of 6- to 7-week-old *Ins1*-Cre mice and control mice (n = 4 *Ins1*-Cre mice, n = 6 control mice). The data are presented as the means ± SEMs.

Figure S9

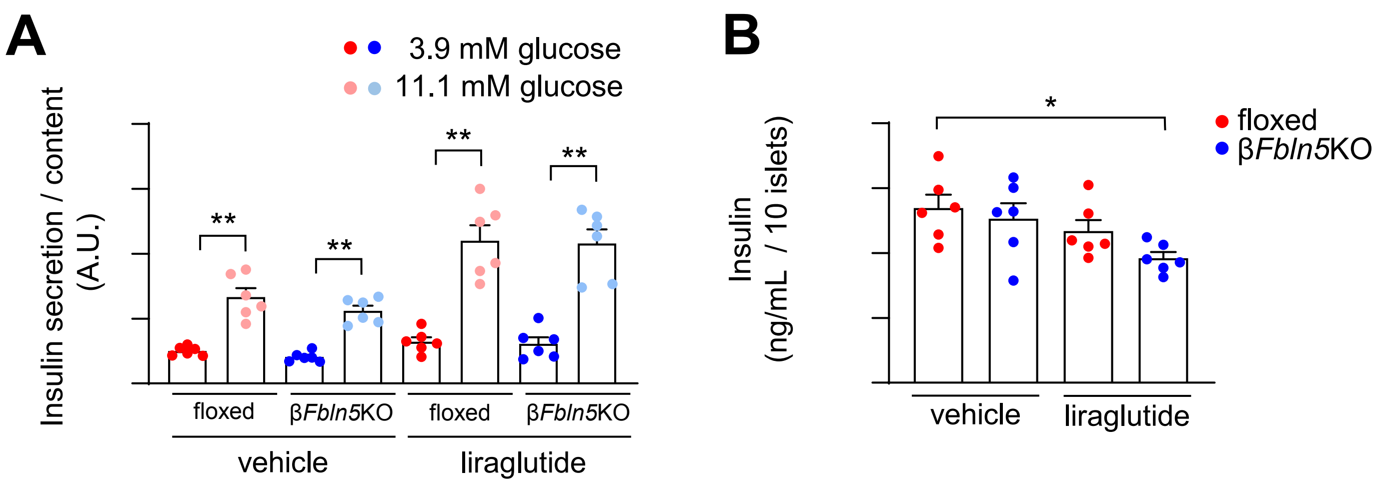

Figure S9. *Fbln5* deletion in pancreatic β-cells did not impair incretin-induced insulin secretion in isolated islets, related to Figure 5.
(A) Glucose-stimulated insulin secretion (GSIS) in 10 isolated islets from 11-week-old β*Fbln5*KO mice and control mice in the presence or absence of liraglutide (n = 6 per group). The data are presented as the means ± SEMs. and ***p* < 0.01, one-way ANOVA, followed by the Tukey HSD post hoc test. (B) Insulin content in 10 isolated islets from 11-week-old β*Fbln5*KO mice and control mice (n = 6 per group) after an incubation with or without 100 nM liraglutide in the presence of 11.1 mM glucose for one hour. The data are presented as the means ± SEMs. **p* < 0.05, one-way ANOVA, followed by the Tukey HSD post hoc test.

Figure S10

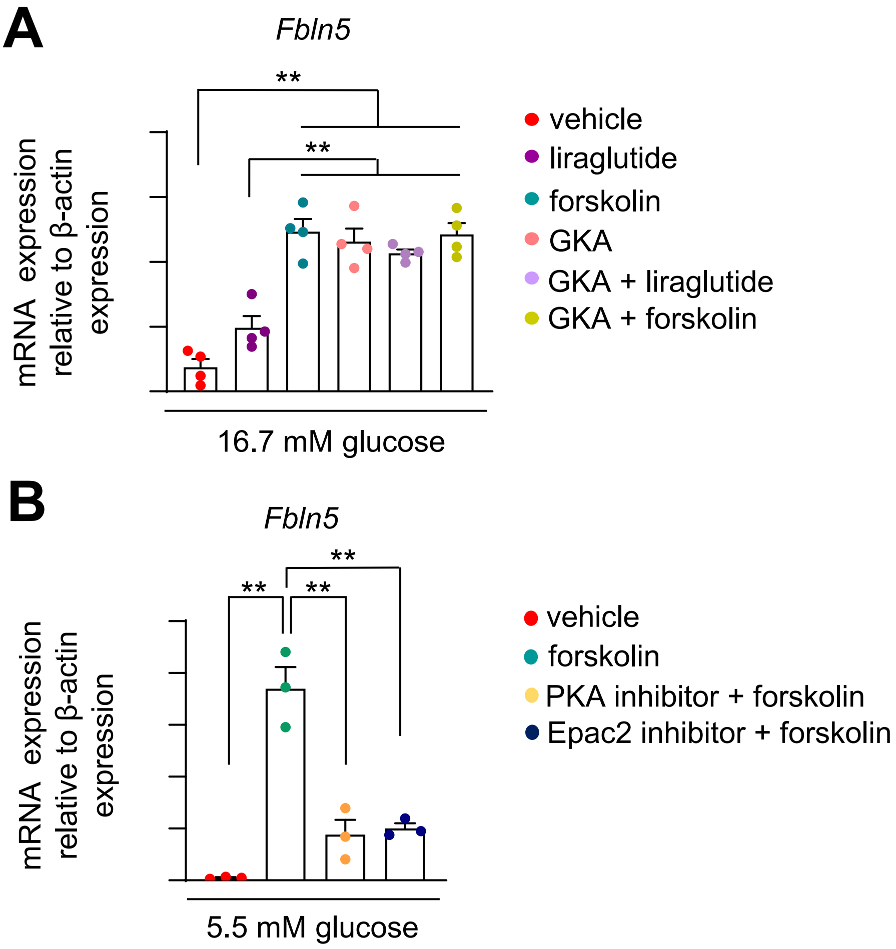

Figure S10. Fbln5 expression was induced by cyclic AMP-mediated signals in islets, related to Figure 5.
(A) *Fbln5* mRNA expression levels in islets isolated from 9-week-old wild-type mice (n = 4 per group). Islets were treated with 100 nM liraglutide or 10 μM forskolin for 24 hours in the presence or absence of 30 μM GKA CpdA and 16.7 mM glucose. The data are presented as the means ± SEMs. $^{**}p < 0.01$, one-way ANOVA, followed by the Tukey HSD post hoc test. (B) *Fbln5* mRNA expression levels in islets isolated from 9-week-old wild-type mice (n = 3 per group). Islets were treated with vehicle, 10 μM forskolin, 10 μM forskolin + 20 μM PKA inhibitor (H-89), or 10 μM forskolin + 50 μM Epac2 inhibitor (ESI-09) for 24 hours in the presence of 5.5 mM glucose. The data are presented as the means ± SEMs. $^{**}p < 0.01$, one-way ANOVA followed by the Tukey HSD post hoc test.

Figure S11

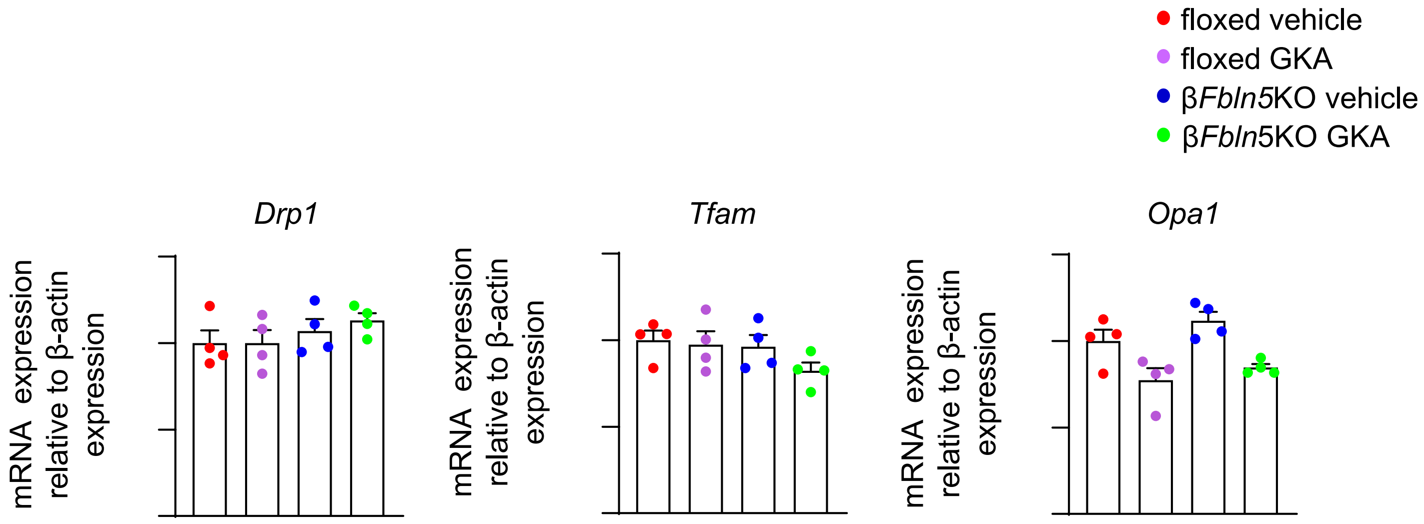

Figure S11. The expression of genes related to mitochondrial function was not altered by *Fbln5* deficiency in β-cells, related to Figure 5.

Drp1, *Tfam*, and *Opa1* mRNA expression in islets from 11-week-old β*Fbln5*KO mice and control littermates after an incubation with or without 30 μM GKA CpdA for 24 hours (n = 4 per group). The data are presented as the means ± SEMs.

Figure S12

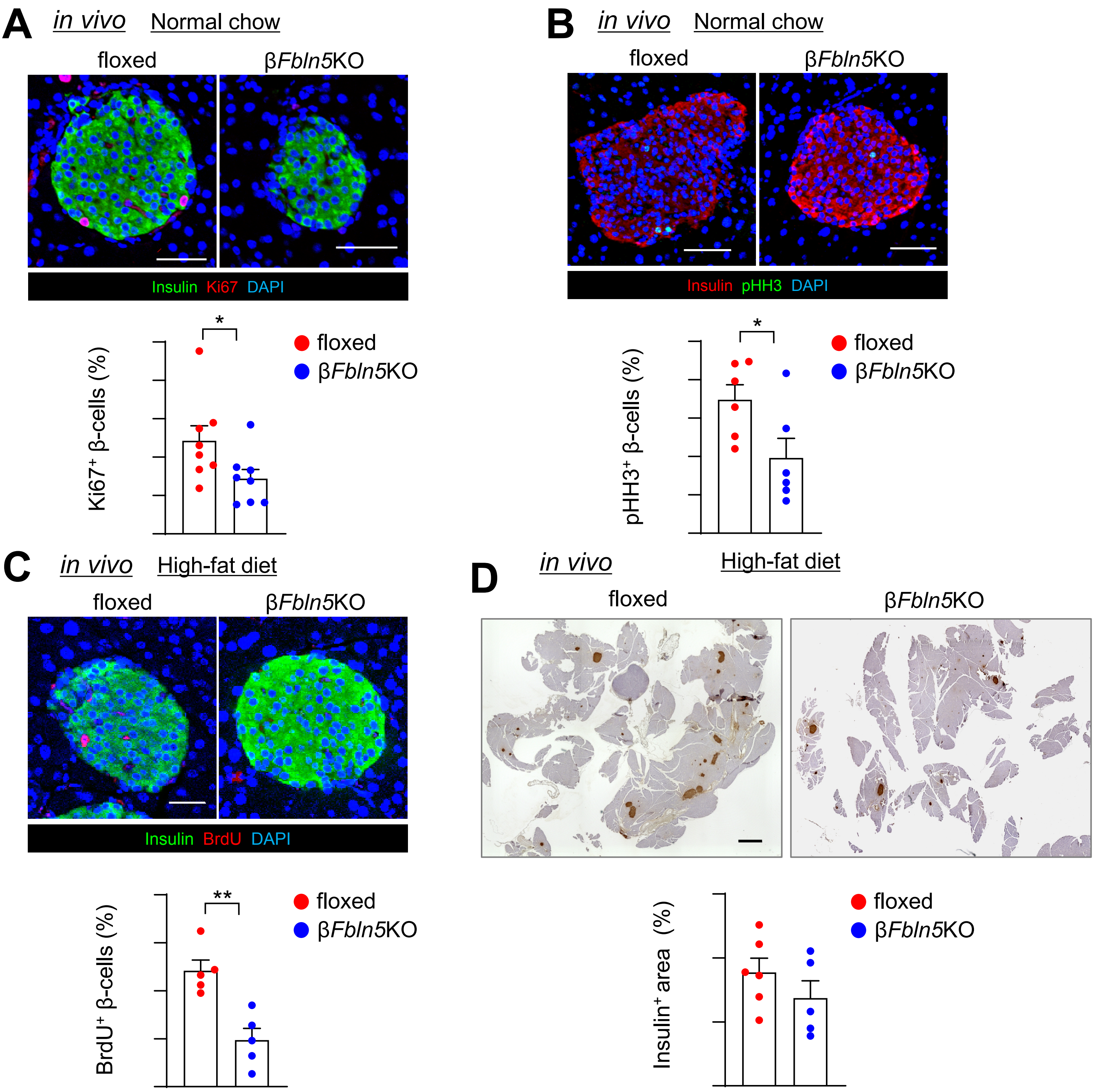

Figure S12. Proliferating β-cells in β-cell-specific *Fbln5*KO mice and high-fat diet-fed mice, related to Figure 7.
(A) Ki67 and (B) pHH3 immunostaining in pancreas sections from 11- to 12-week-old β*Fbln5*KO mice and control mice. Representative images of pancreatic islets (upper panel) and the percentage of Ki67-positive or pHH3-positive β-cells among insulin-positive β-cells (lower panel) are shown (n = 6–8 mice per group). In the Ki67 images, insulin is stained green, the cell nuclei are stained blue with DAPI, and the Ki67-positive nuclei are stained red. In the pHH3 images, insulin in β-cells is stained red, the cell nuclei are stained blue with DAPI, and the pHH3-positive nuclei are stained green. The scale bar represents 50 μm. The data are presented as the means ± SEMs. *$p < 0.05$, Student's t test. (C) BrdU incorporation in pancreas sections from β*Fbln5*KO mice and control mice fed a high-fat diet for 12 weeks. BrdU was administered 6 hours before the animals were sacrificed. Representative images of pancreatic islets (left panel) and the percentage of BrdU-positive β-cells among insulin-positive β-cells (right panel) are shown (n = 5 mice per group). Insulin is stained green, the cell nuclei are stained blue with DAPI, and the BrdU-positive nuclei are stained red. The scale bar represents 50 μm. The data are presented as the means ± SEMs. **$p < 0.01$, Student's t test. (D) β-Cell mass in β*Fbln5*KO mice and control mice fed a high-fat diet for 12 weeks (n = 5–6). Representative images of the pancreas showing brown staining for insulin and the ratio of the β-cell mass relative to the area of the whole pancreas. The scale bar represents 500 μm. The data are presented as the means ± SEMs.

Figure S13

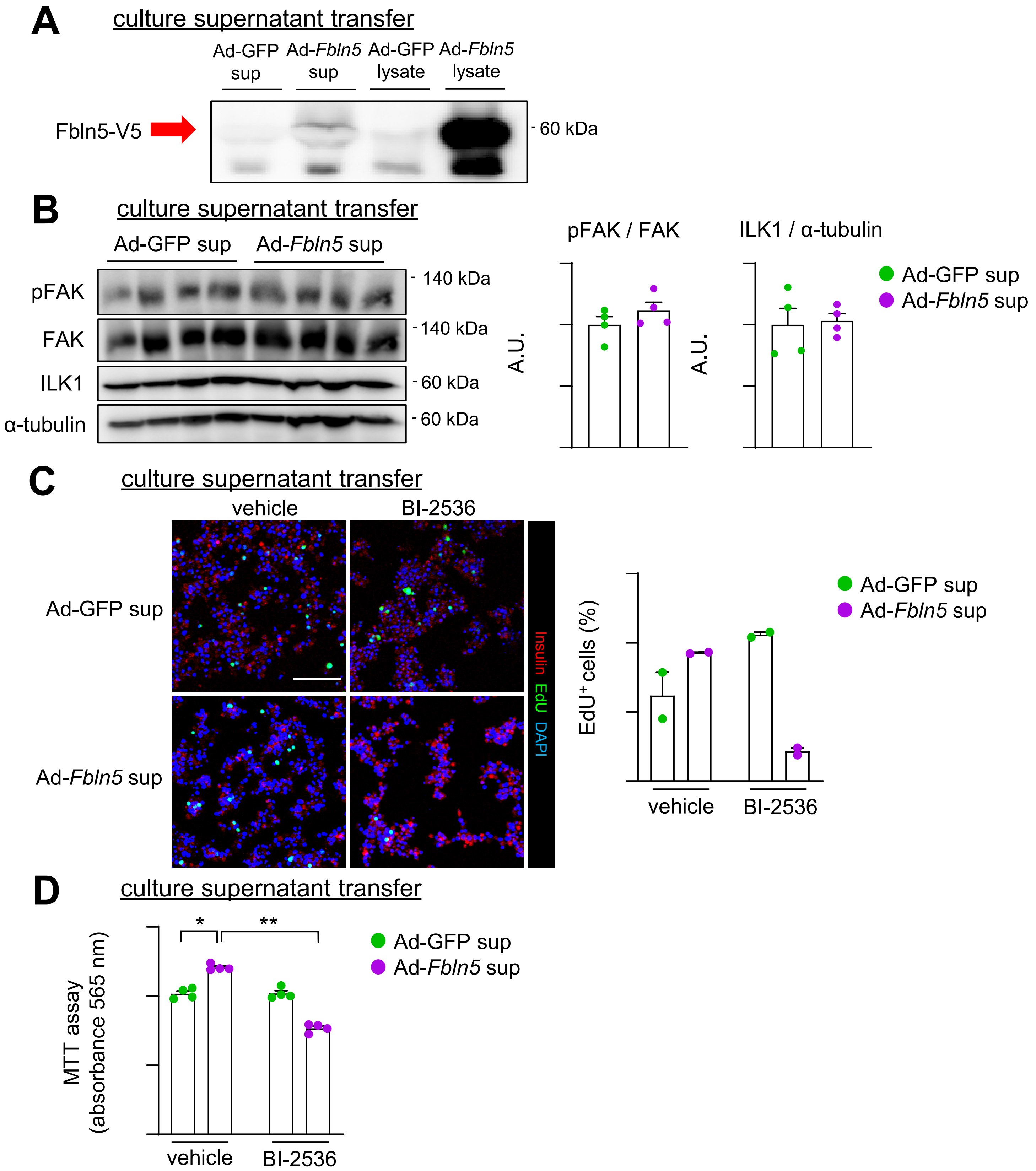

Figure S13. Effect of the PLK1 inhibitor on cell proliferation and integrin signaling in INS-1 cells treated with the culture supernatant from *Fbln5*-overexpressing INS-1 cells, related to Figure 10.

(A) Immunoblot showing the levels of the V5 tag in the culture media of V5-tagged Ad-*Fbln5*-infected islets, Ad-GFP-infected islets, and each islet lysate. The culture media were collected 72 hours after infection. (B) Immunoblots showing the levels of FAK and proteins in the ILK pathway in INS-1 cells treated with the culture supernatant from Ad-*Fbln5*- or Ad-GFP-infected INS-1 cells for 24 hours in the presence of 5.5 mM glucose (n = 4). The culture supernatant was collected 72 hours after the infection. Densitometry data are plotted in the lower graphs (n = 4). The data are presented as the means ± SEMs. (C) INS-1 cells were treated with the culture supernatant from Ad-*Fbln5*- or Ad-GFP-infected INS-1 cells for 72 hours in the presence or absence of 50 nM BI-2536. The percentages of insulin-positive INS-1 cells that were positive for EdU are shown (n = 2). The scale bar represents 200 μm. The data are presented as the means ± SEMs. (D) MTT cell proliferation assay in INS-1 cells treated with culture supernatant from Ad-*Fbln5*- or Ad-GFP-infected INS-1 cells for 72 hours in the presence or absence of 50 nM BI-2536. The data are presented as the means ± SEMs. *$p < 0.05$, **$p < 0.01$, two-way ANOVA.

Table 1.

Top 20 upregulated genes in β*Fbln5*KO islets revealed by the microarray analysis.

FC	Gene Symbol	Gene Name
58.55	Slc38a1	Solute carrier family 38, member 1
17.66	Esp36	exocrine gland secreted peptide 36
15.38	Sft2d1	SFT2 domain containing 1
15.22	Olfr390	olfactory receptor 390
15.06	Jakmip2	janus kinase and microtubule interacting protein 2
14.75	Olfr630	olfactory receptor 630
14.28	Ccdc141	coiled-coil domain containing 141
13.75	Olfr938	olfactory receptor 938
11.19	Olfr491	olfactory receptor 491
9.66	Slco1b2	solute carrier organic anion transporter family, member 1b2
9.66	Cyp4a12a	cytochrome P450, family 4, subfamily a, polypeptide 12a
9.27	Tnfrsf22	tumor necrosis factor receptor superfamily, member 22
8.19	Olfr707	olfactory receptor 707
7.67	Ugt1a10	UDP glycosyltransferase 1 family, polypeptide A10
7.49	Olfr1240	olfactory receptor 1240
7.29	Ntn5	netrin 5
6.65	Cxcl9	chemokine (C-X-C motif) ligand 9
6.53	Chrnb4	cholinergic receptor, nicotinic, beta polypeptide 4
6.44	Uroc1	urocanase domain containing 1
6.26	Vmn1r223	vomeronasal 1 receptor 223

Table 2.

Top 20 downregulated genes in β*Fbln5*KO islets revealed by the microarray analysis.

FC	Gene Symbol	Gene Name
19.04	Sema3d	sema domain, immunoglobulin domain (Ig), short basic domain, secreted, (semaphorin) 3D
16.87	Krt13	keratin 13
11.90	Gpr33	G protein-coupled receptor 33
5.22	Slc5a2	solute carrier family 5 (sodium/glucose cotransporter), member 2
4.75	Rbbp8nl	RBBP8 N-terminal like
4.67	Wdsub1	WD repeat, SAM and U-box domain containing 1
4.32	Slc23a2	solute carrier family 23 (nucleobase transporters), member 2
4.21	Slc43a1	solute carrier family 43, member 1
4.10	Onecut1	one cut domain, family member 1
3.79	Pgbd1	piggyBac transposable element derived 1
3.74	Adgb	androglobin
3.61	Zcwpw1	zinc finger, CW type with PWWP domain 1
3.48	Olfr1294	olfactory receptor 1294
3.46	Adamts10	A disintegrin and metalloproteinase with thrombospondin motifs 10
3.38	Zfp12	zinc finger protein 12
3.30	Ppp1r12b	protein phosphatase 1, regulatory (inhibitor) subunit 12B
3.08	Adcy10	adenylate cyclase 10
3.06	Naip6	NLR family, apoptosis inhibitory protein 6
2.98	Krt2	keratin 2
2.93	Nup98	nucleoporin 98

Data S1: uncropped images of immunoblots, related to Figure 1-11 and Figure S1-S13.

Figure 1E

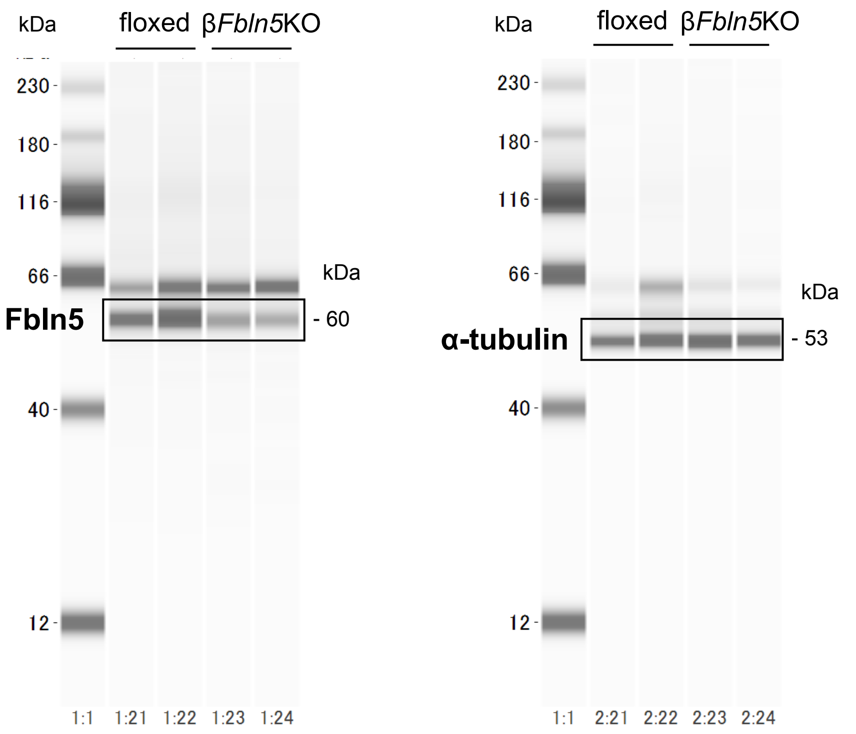

Figure 8D

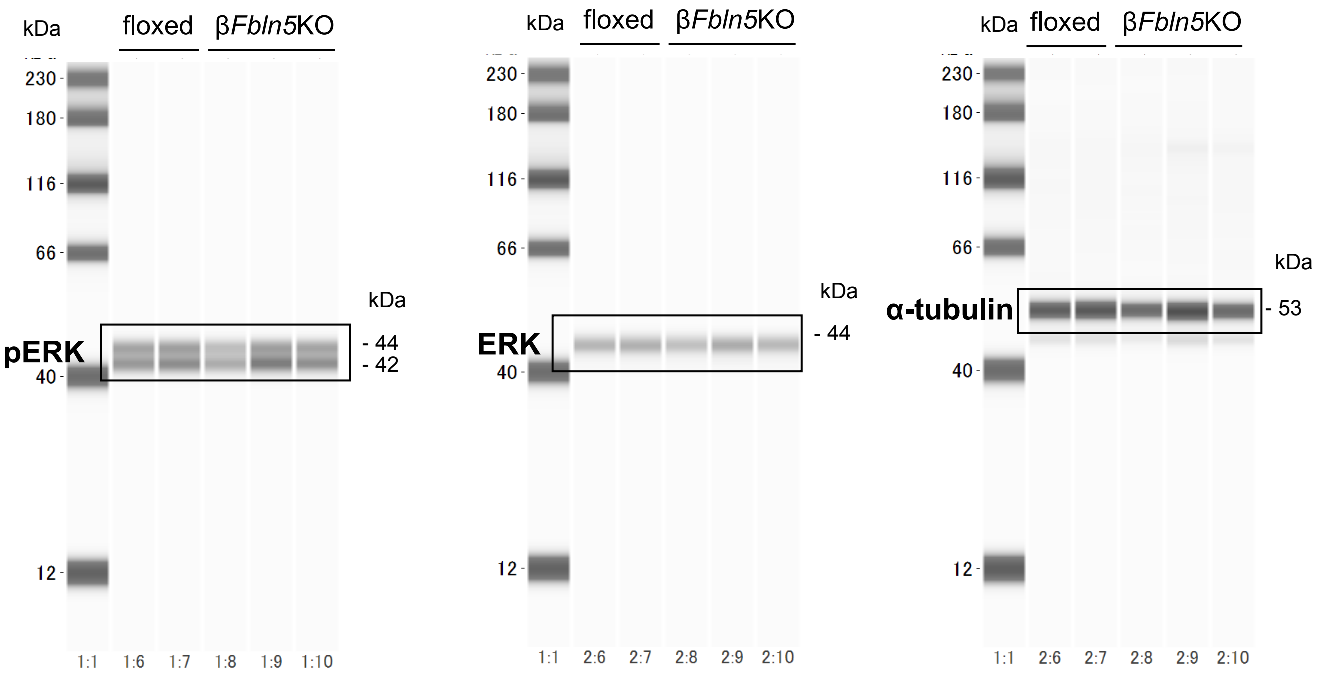

Data S1: uncropped images of immunoblots, related to Figure 1-11 and Figure S1-S13.

Figure 10C

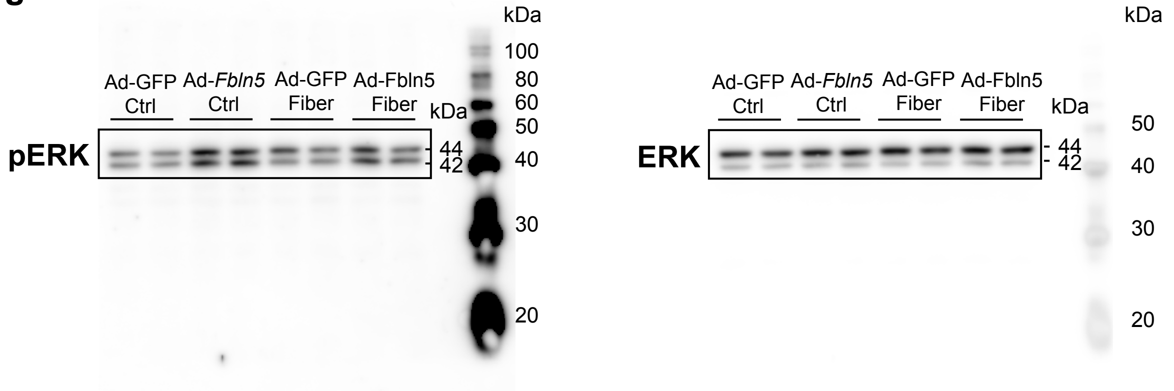

Figure S1B:same as Figure 1E

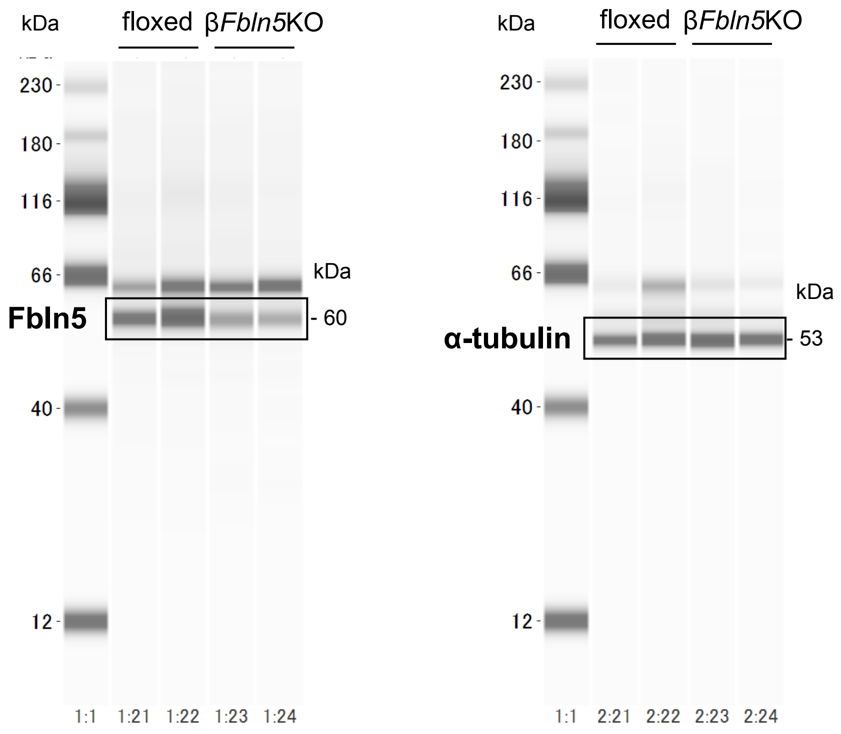

Figure S13A

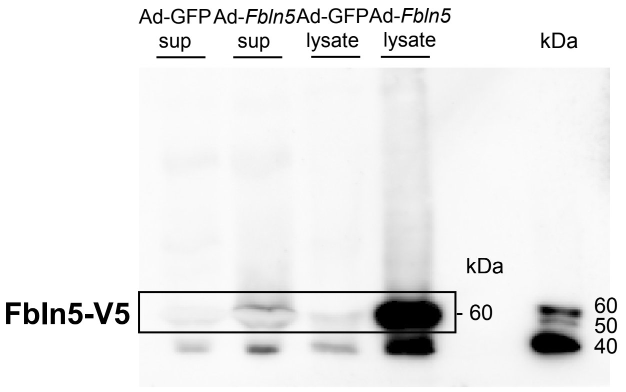

Figure S13B

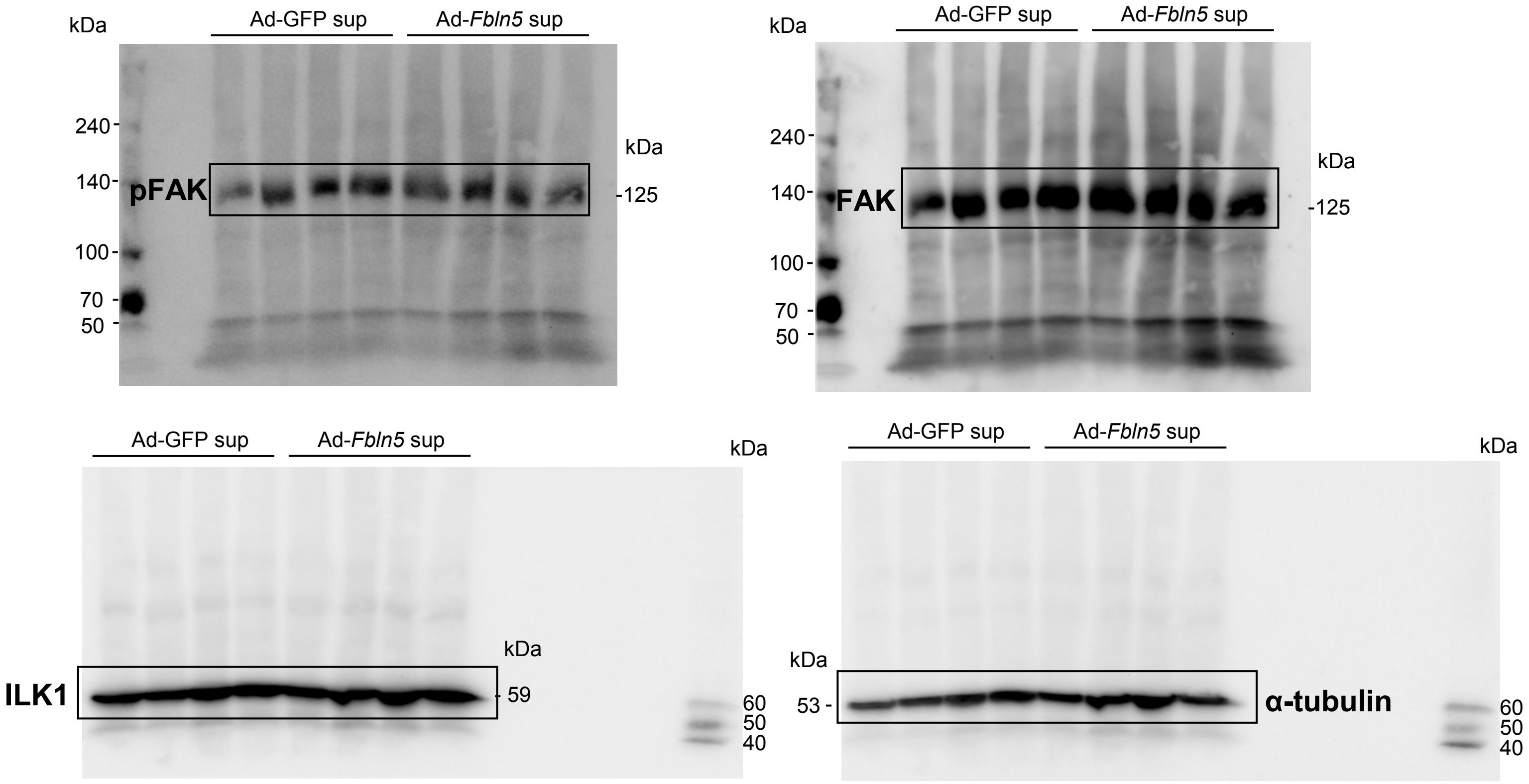

Data S2: uncropped images of immunostainings, related to Figure 1-11 and Figure S1-S13.

Figure 2A

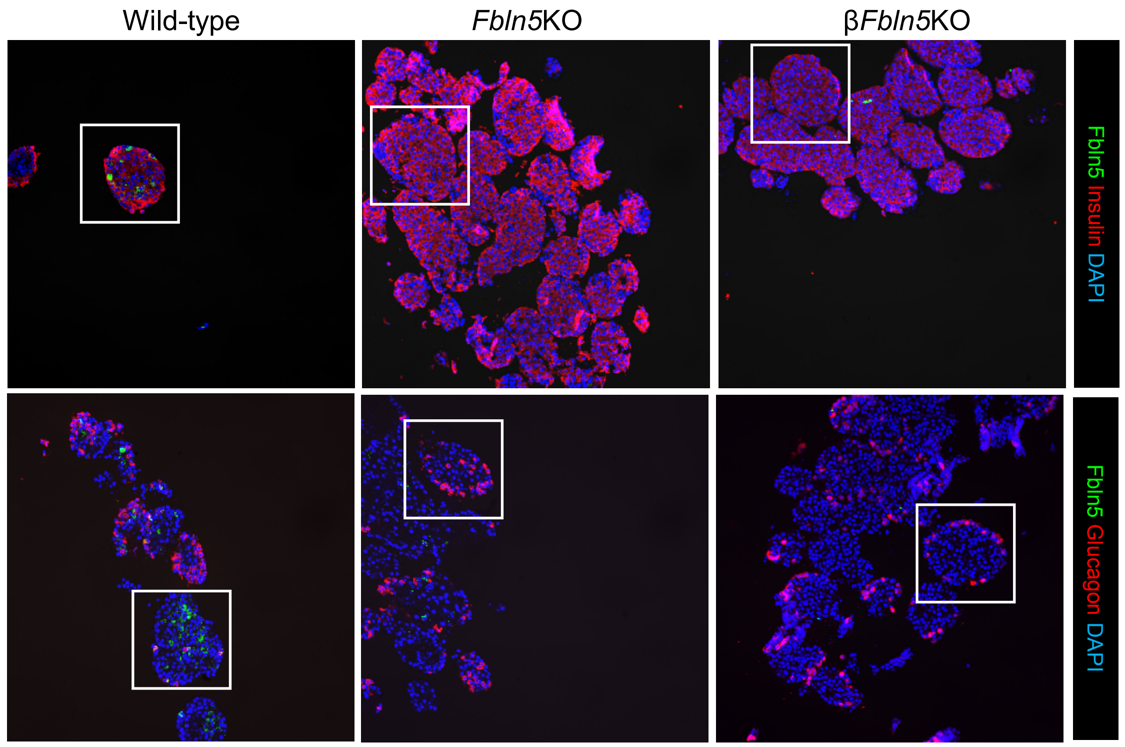

Figure 2B

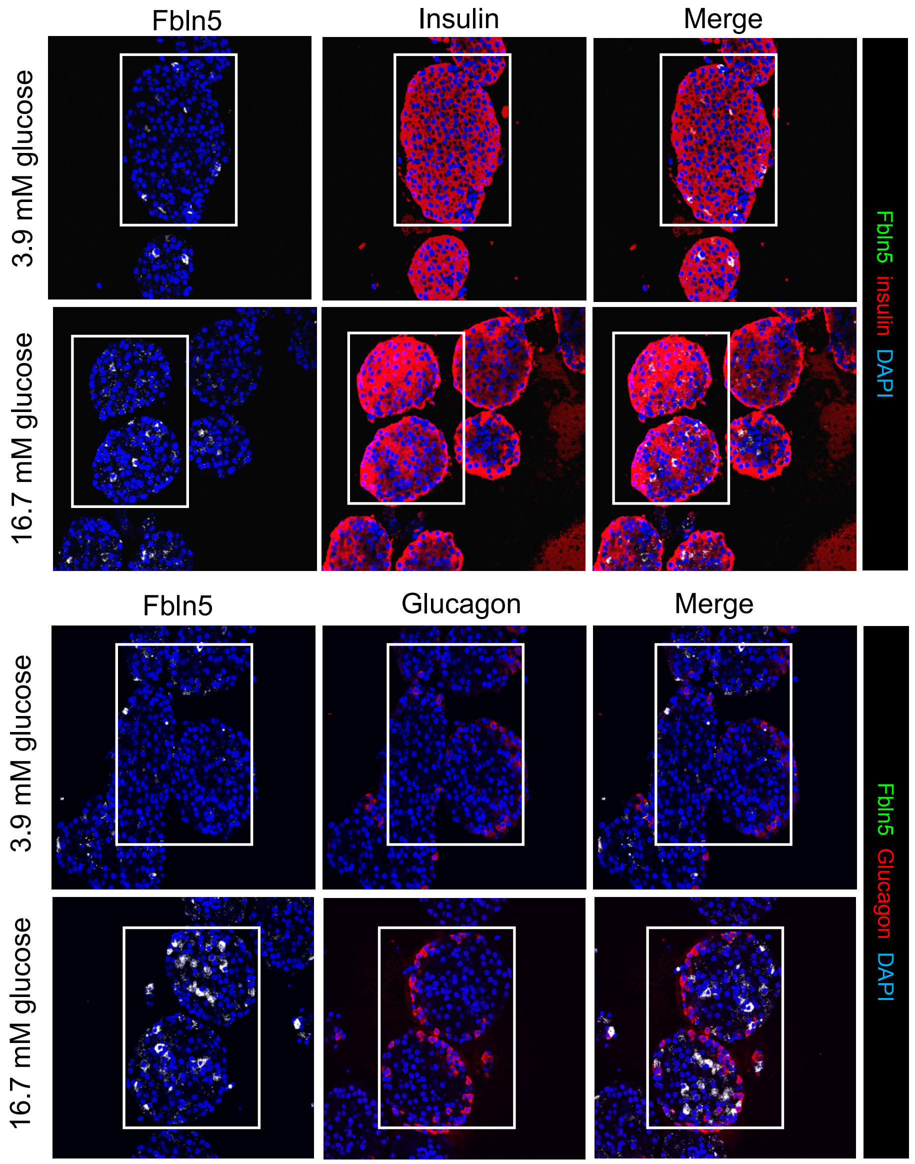

Data S2: uncropped images of immunostainings, related to Figure 1-11 and Figure S1-S13.

Figure 2C

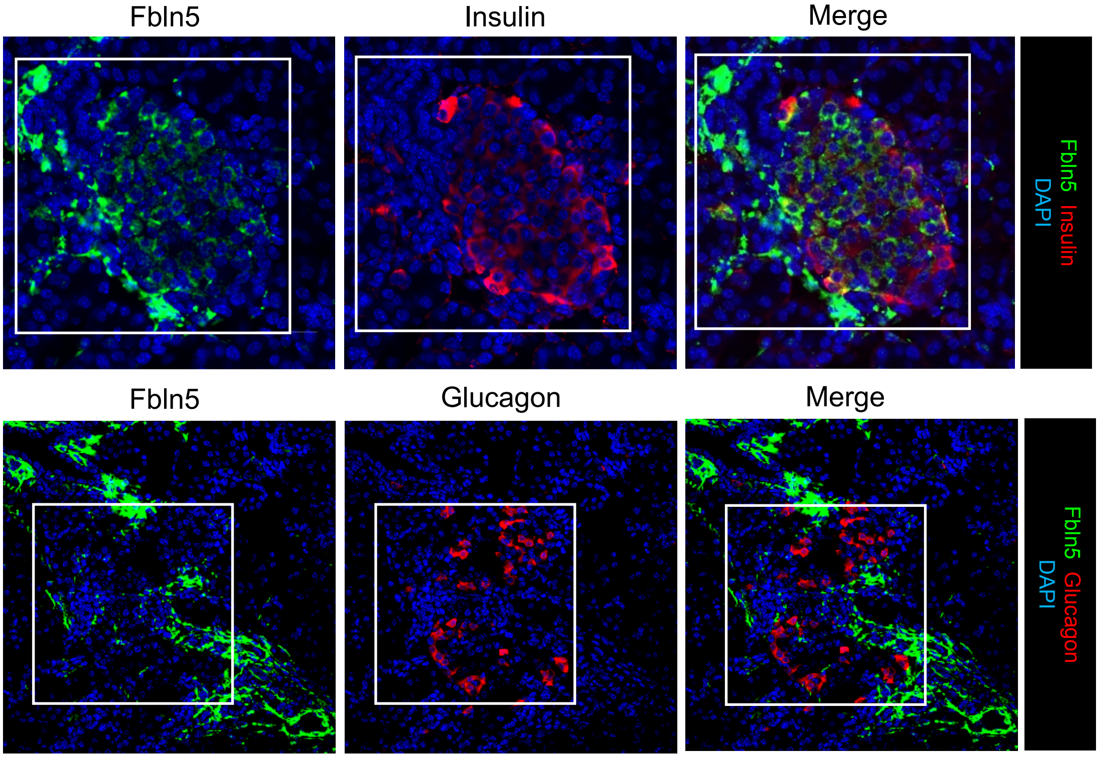

Figure 2D

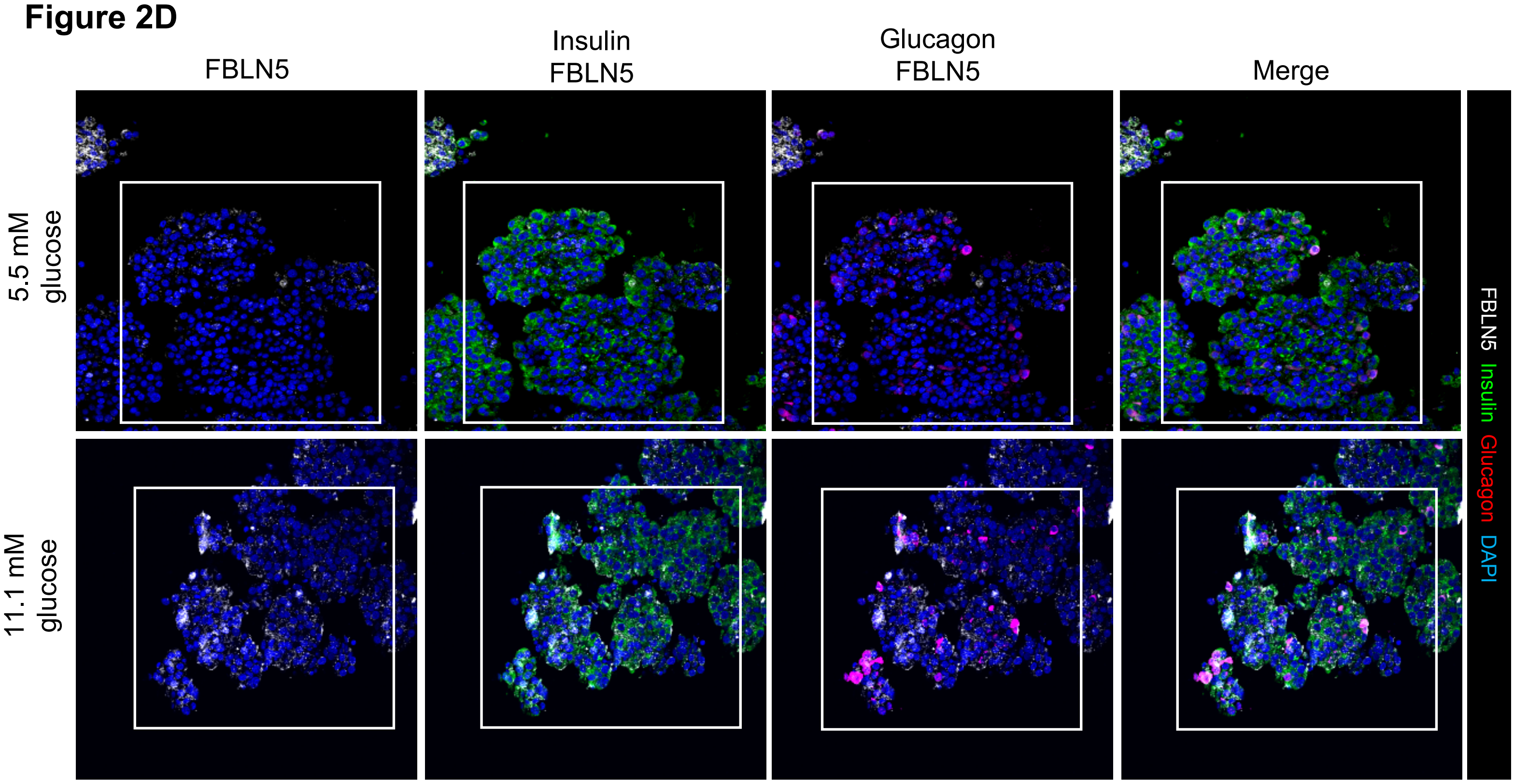

Figure 5D

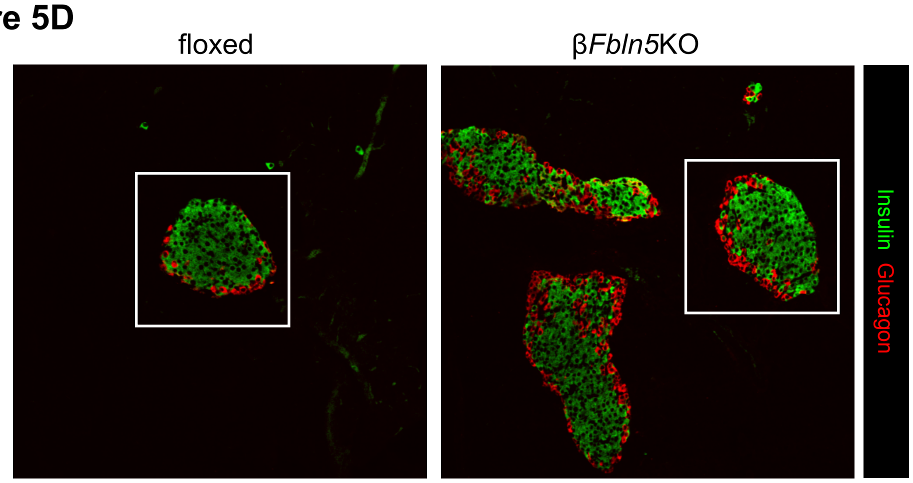

Data S2: uncropped images of immunostainings, related to Figure 1-11 and Figure S1-S13.

Figure 7A

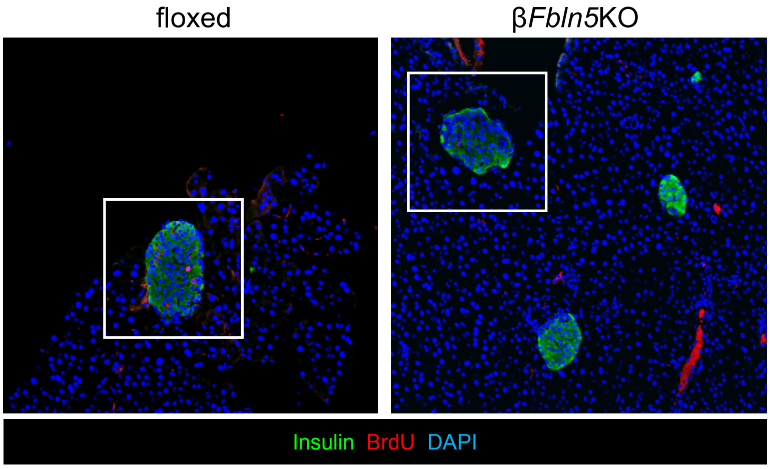

Figure 7B

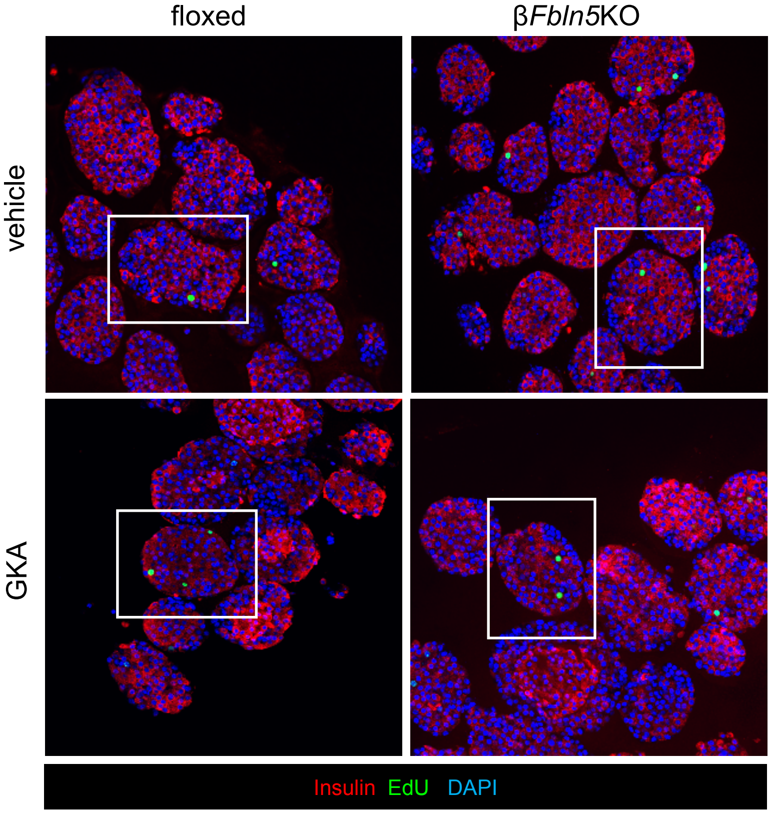

Figure 7C

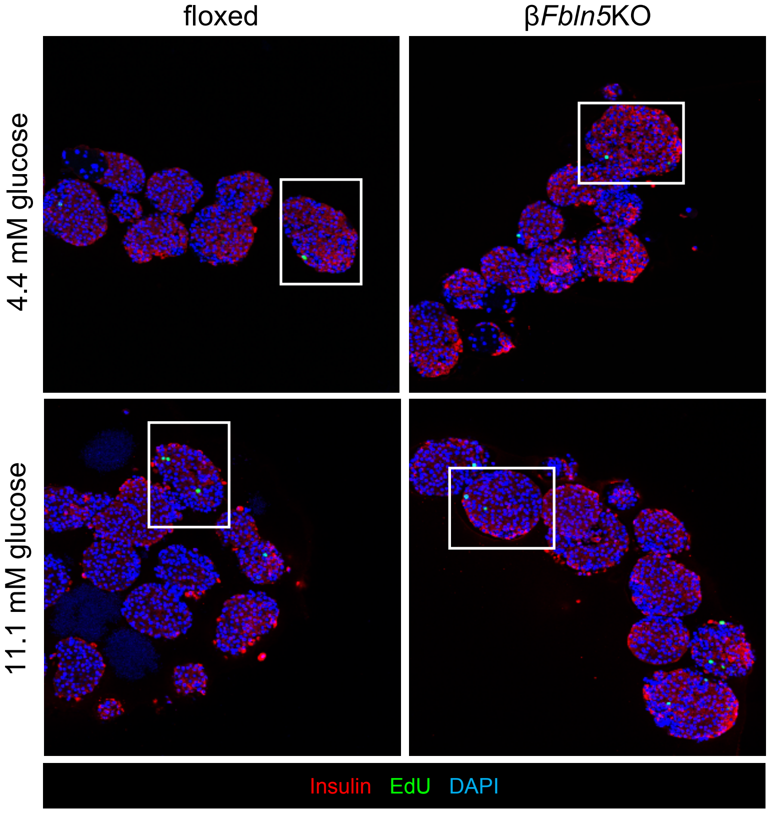

Figure 7D

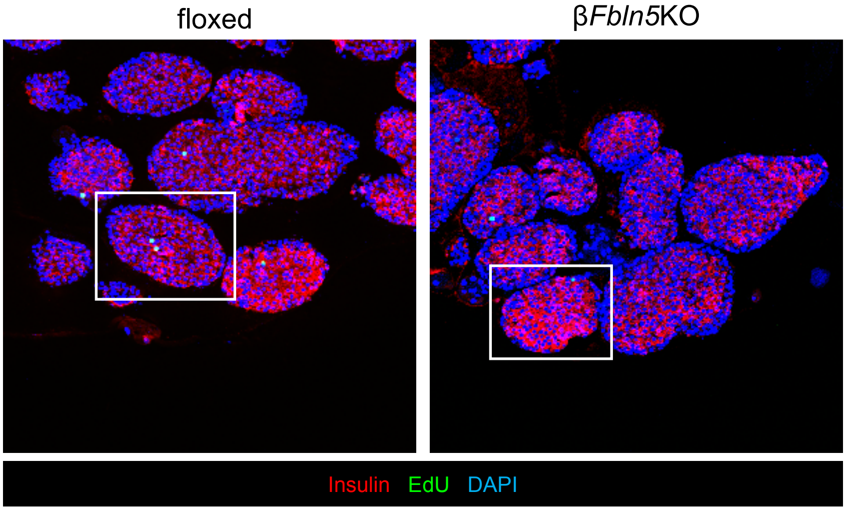

Data S2: uncropped images of immunostainings, related to Figure 1-11 and Figure S1-S13.

Figure 8A

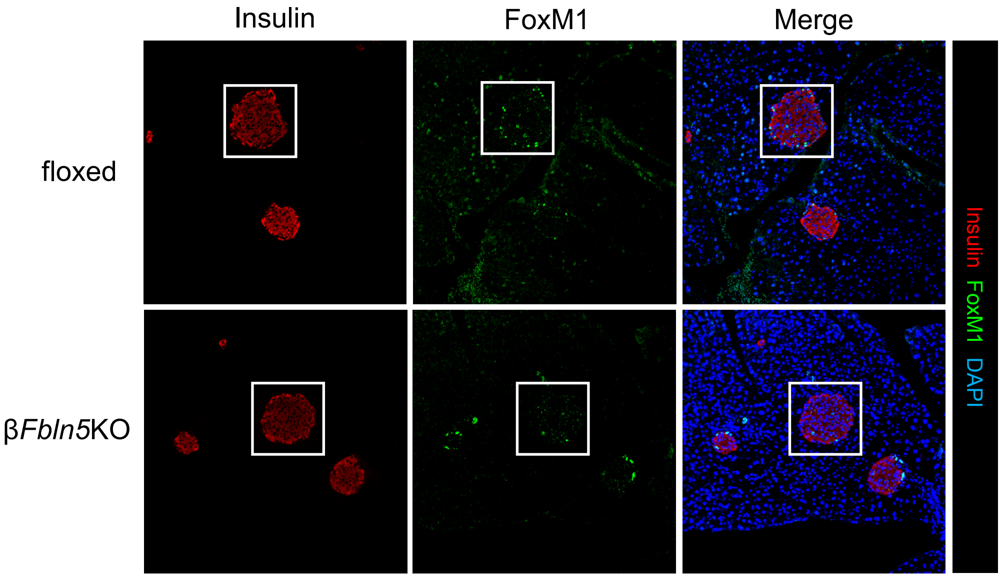

Figure 8B

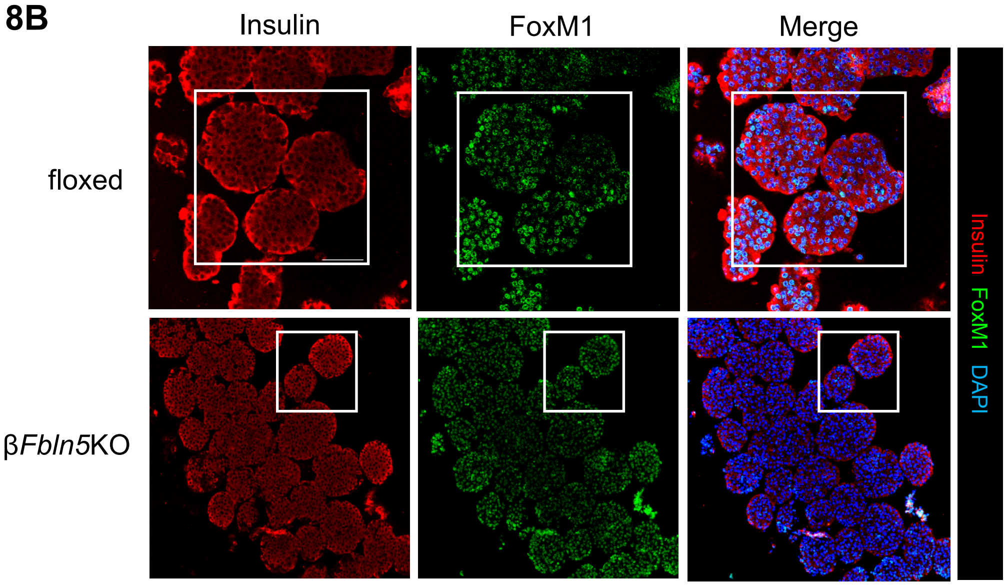

Figure 8C

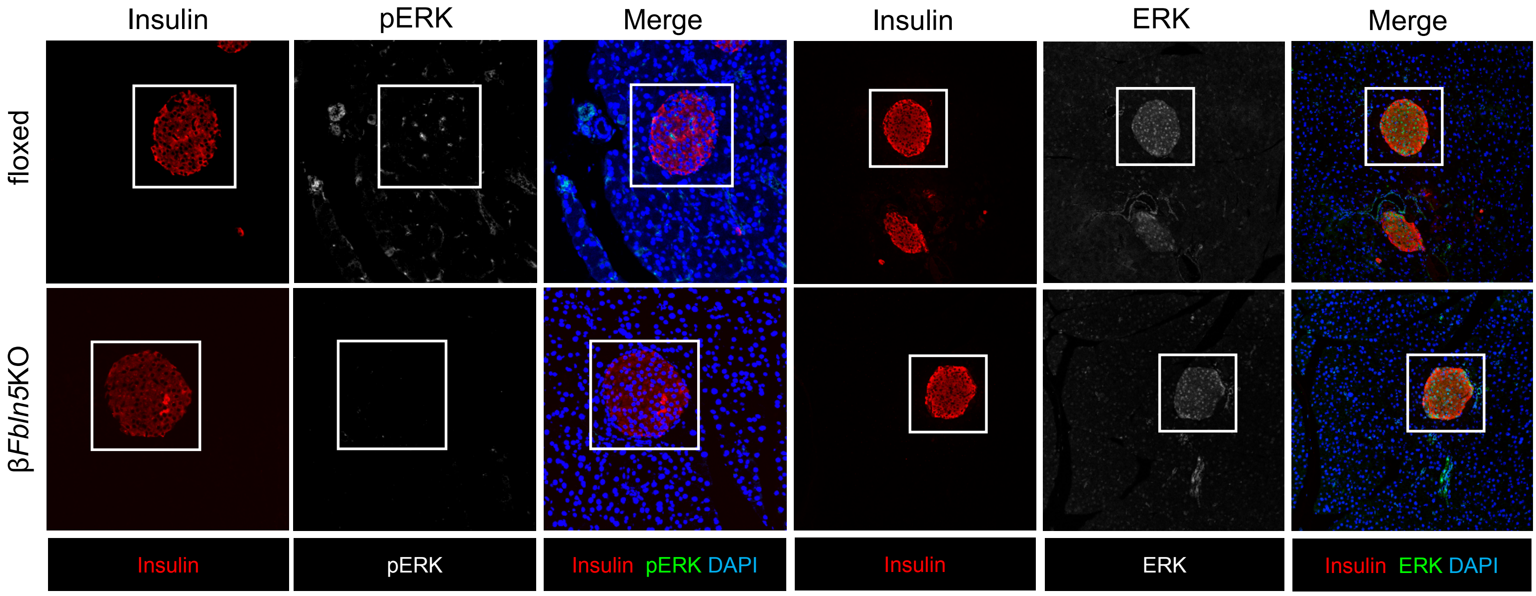

Data S2: uncropped images of immunostainings, related to Figure 1-11 and Figure S1-S13.

Figure 9A

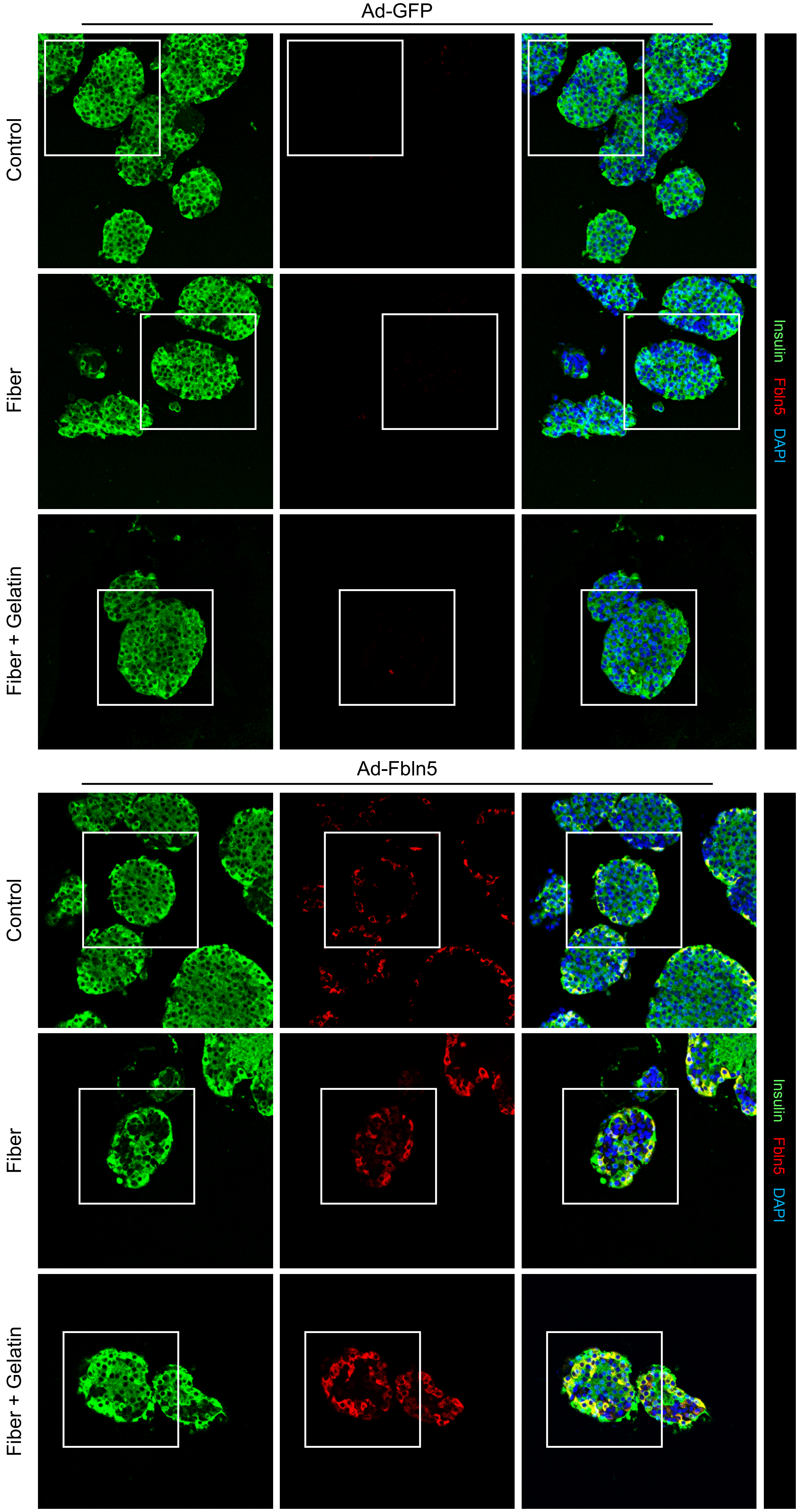

Data S2: uncropped images of immunostainings, related to Figure 1-11 and Figure S1-S13.

Figure 9B

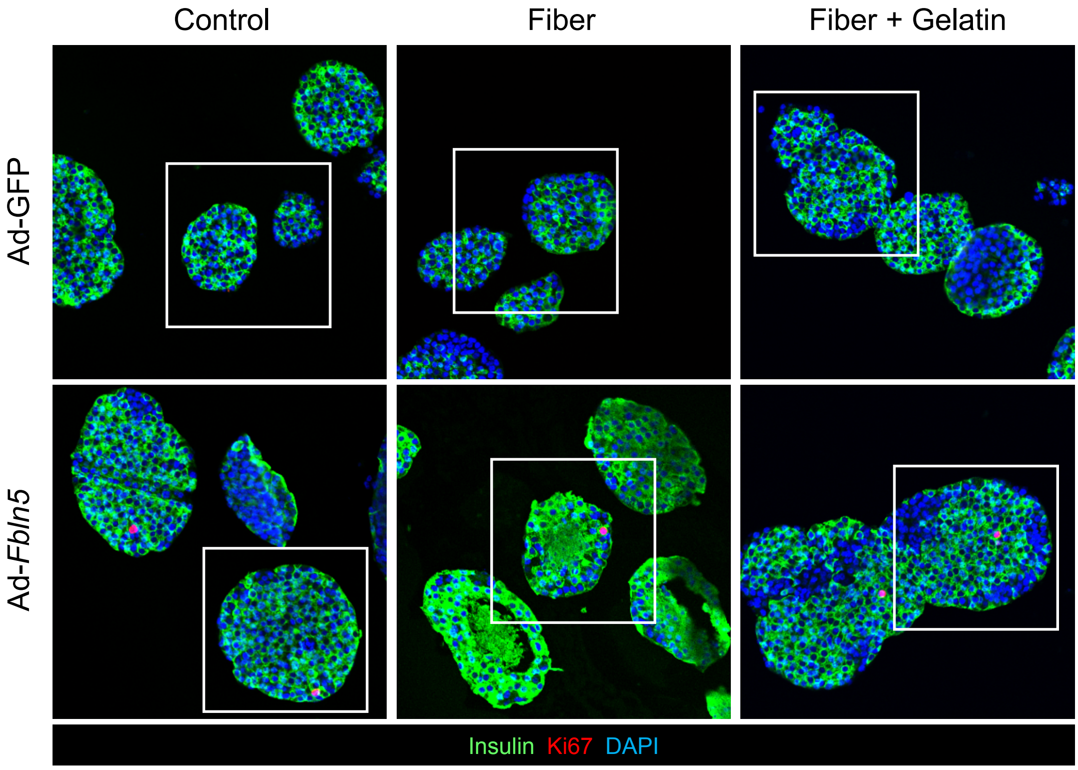

Figure 9C

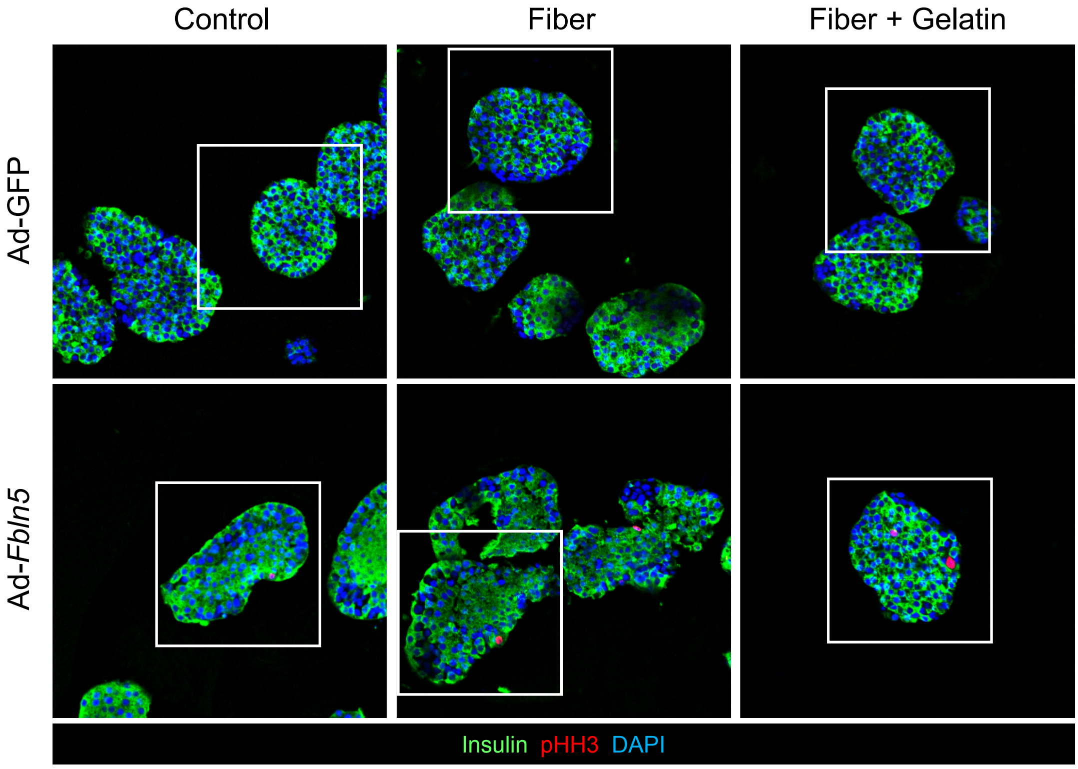

Data S2: uncropped images of immunostainings, related to Figure 1-11 and Figure S1-S13.

Figure 10A

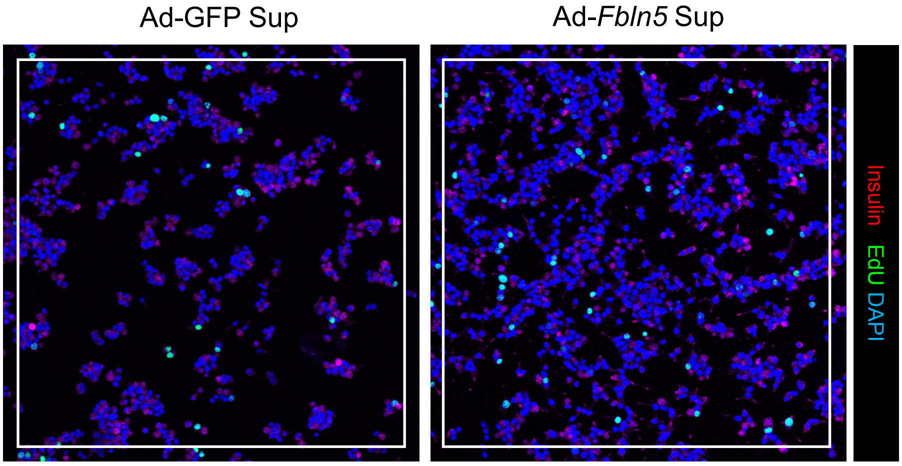

Figure 10B

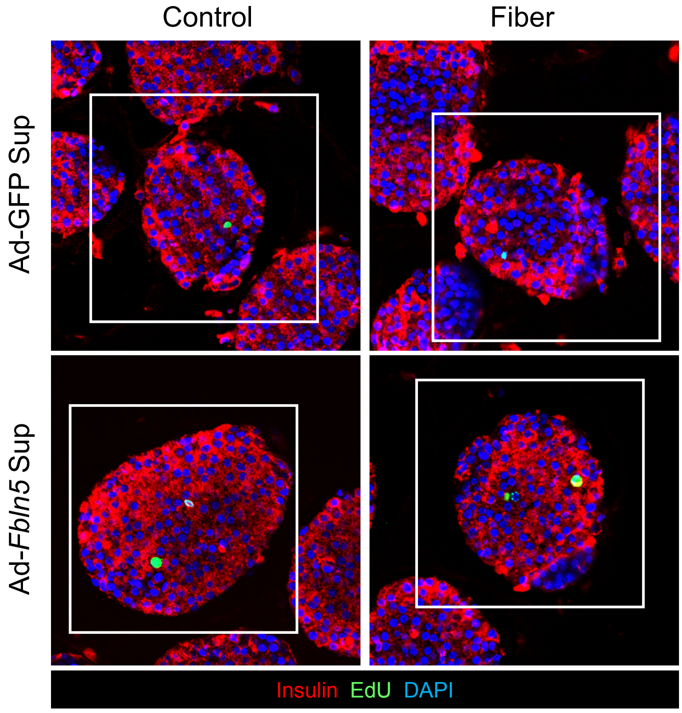

Figure 10D

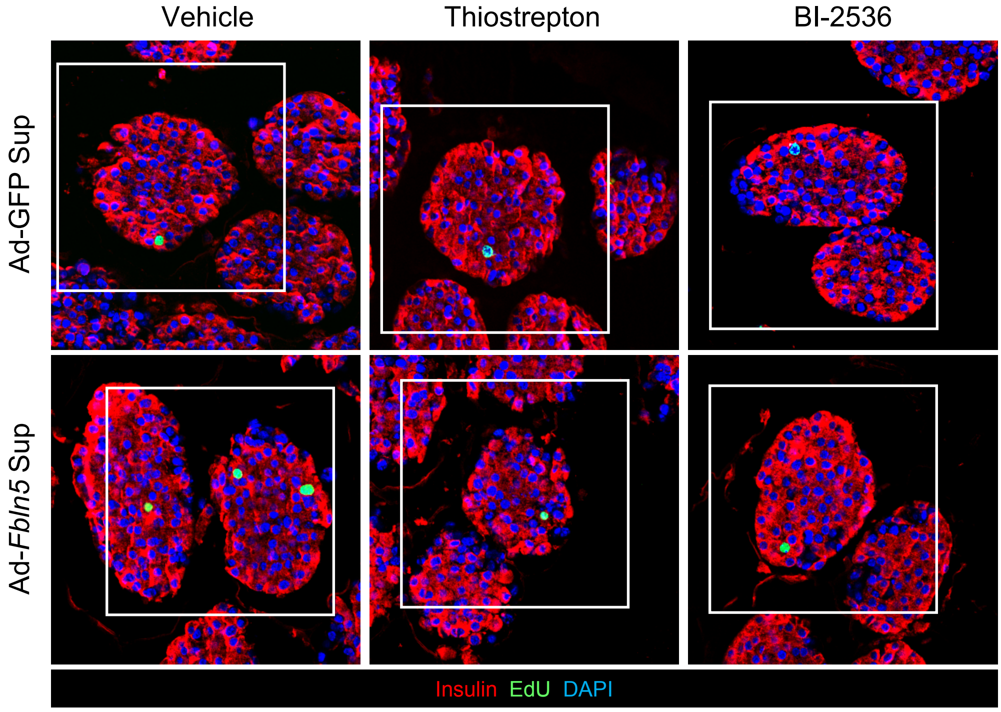

Data S2: uncropped images of immunostainings, related to Figure 1-11 and Figure S1-S13.

Figure S2

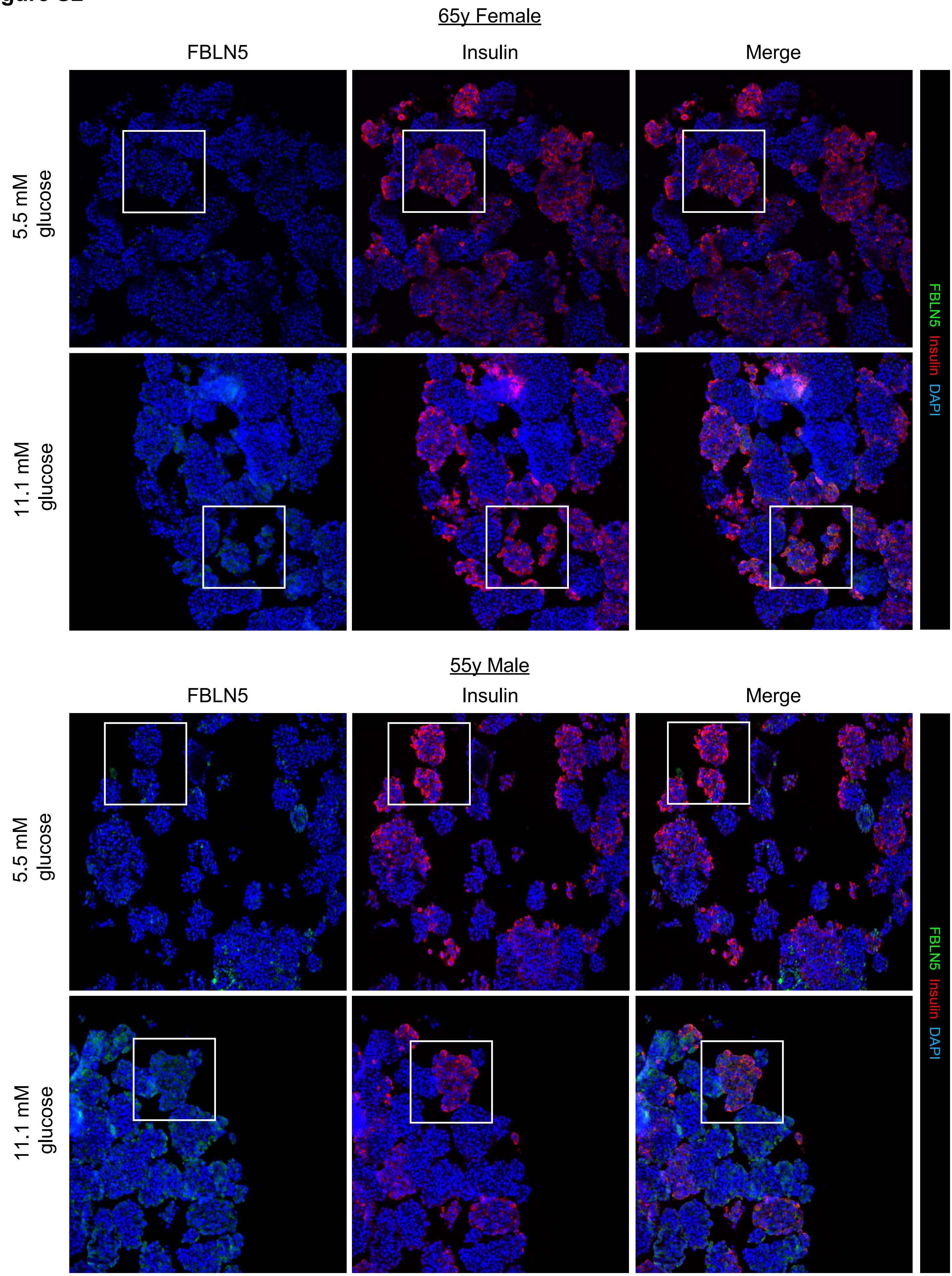

Data S2: uncropped images of immunostainings, related to Figure 1-11 and Figure S1-S13.

Figure S3

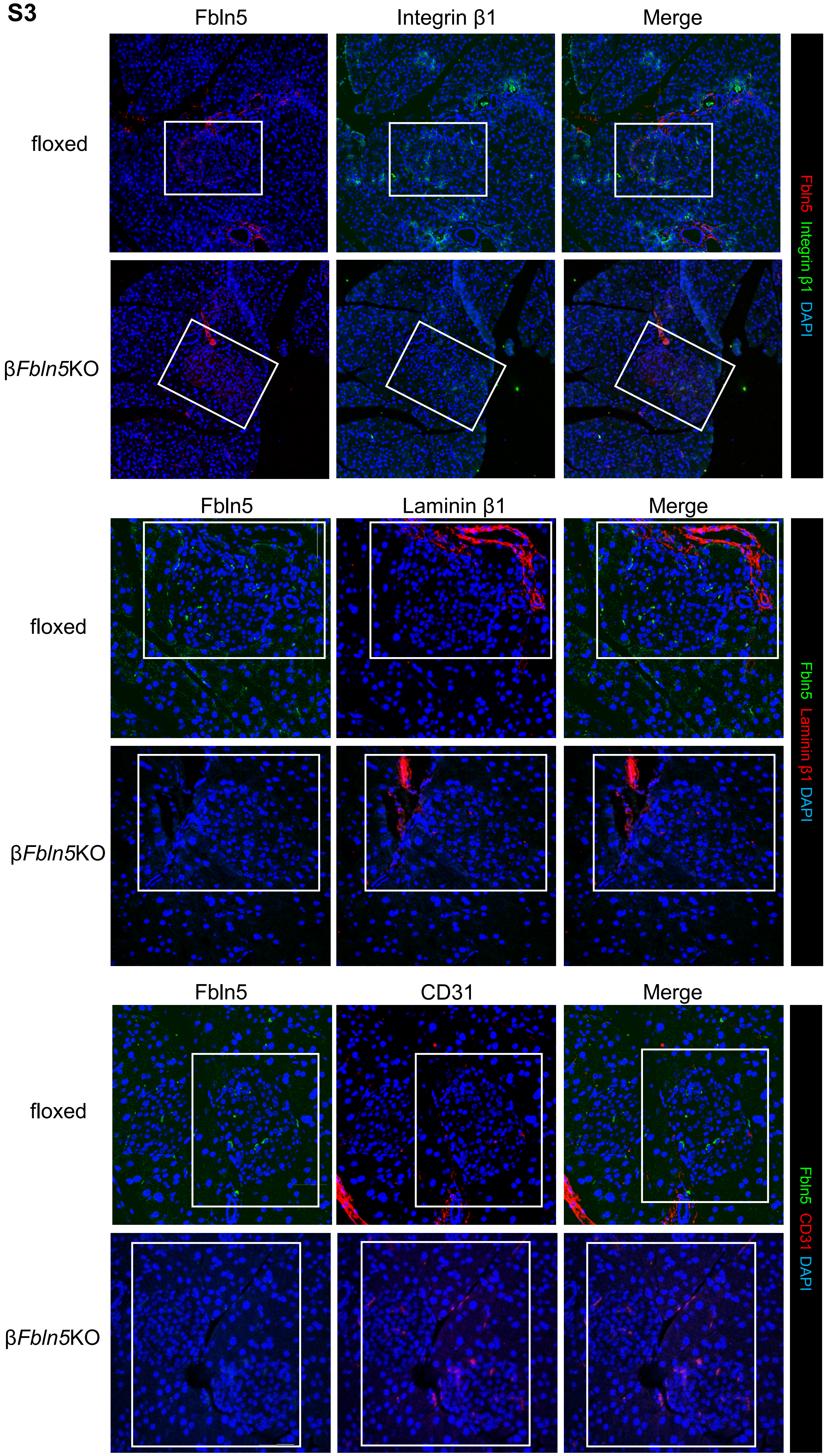

Data S2: uncropped images of immunostainings, related to Figure 1-11 and Figure S1-S13.

Figure S12A

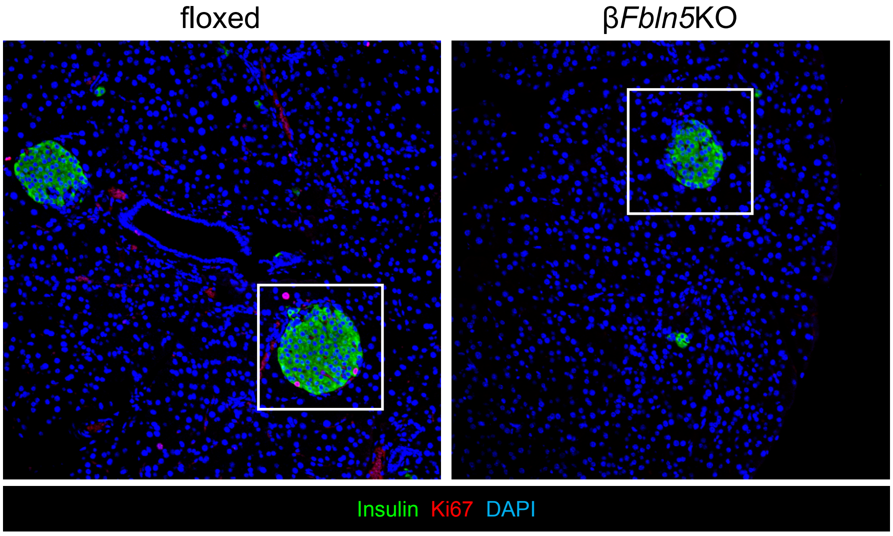

Figure S12B

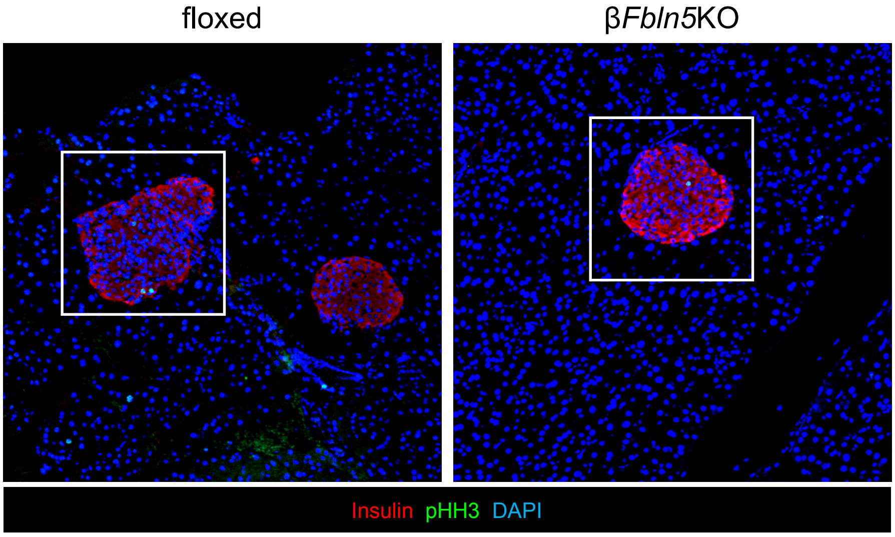

Figure S12C

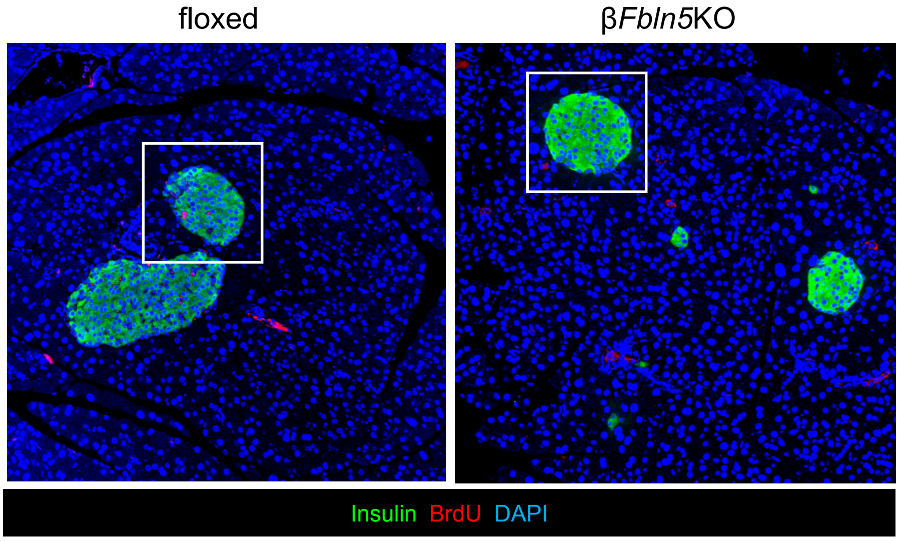

Data S2: uncropped images of immunostainings, related to Figure 1-11 and Figure S1-S13.

Figure S13C

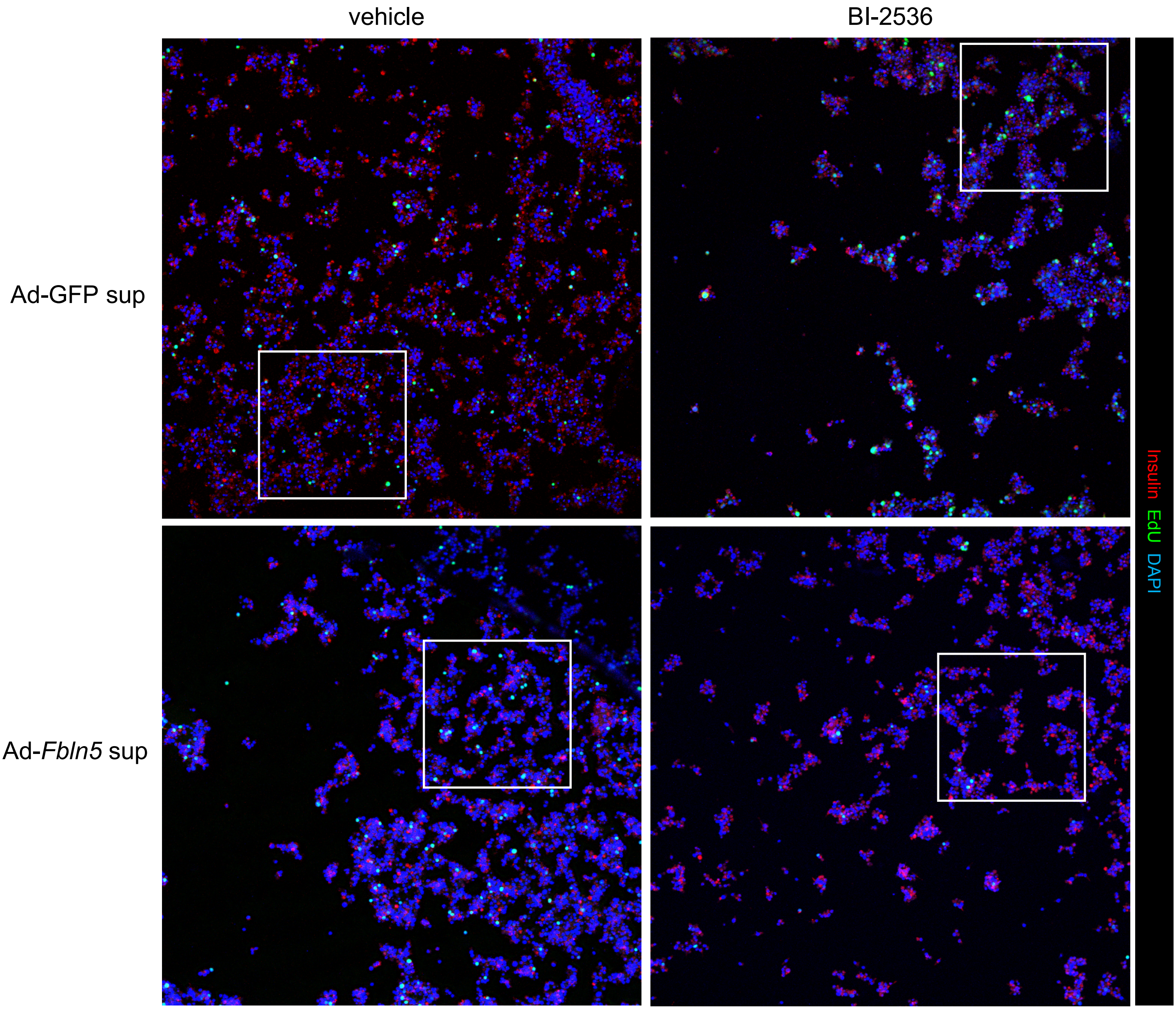

Supplement: Document S1. Figures S1–S13, Tables S1 and S2, and Data S1 and S2 [file mmc1.pdf]
